# The *prc* gene as a high-resolution proxy for *Aeromonas* genus-wide clonal genealogy, phylogenomic validation and superiority over traditional MLST

**DOI:** 10.1038/s41598-026-67534-6

**Published:** 2026-08-27

**Authors:** Bahaa Abdella, Nermeen M. Shokrak, Radi A. Mohamed, Ehab R. El-Helow

**Affiliations:** 1 Aquaculture Department, Faculty of Aquatic and Fisheries Sciences, Kafrelsheikh University, Kafrelsheikh, 33516 Egypt; 2 Department of Botany & Microbiology, Faculty of Science, Alexandria University, Alexandria, 21511 Egypt

**Keywords:** *Aeromonas*, Comparative genomics, Taxonomic misclassification, Molecular marker, Phylogenomics, Microbial evolution, Pathogen surveillance, Computational biology and bioinformatics, Evolution, Genetics, Microbiology

## Abstract

*Aeromonas* spp. are critical aquatic pathogens affecting global aquaculture and human health, yet their taxonomy is frequently confounded by persistent horizontal gene transfer and high recombination rates. While whole-genome sequencing offers definitive resolution, many laboratories remain dependent on biochemical profiling or multi-locus sequence typing (MLST) due to cost and infrastructure constraints. To address this diagnostic gap, a two-phase phylogenomic study to identify a robust single locus genetic marker was conducted. In the discovery phase, we analyzed 22 high-quality complete *Aeromonas* type strain genomes to quantify the evolutionary impact of recombination and selection of new marker candidate, followed by a validation phase involving high-quality 374 genus-wide assemblies. Our results indicate that an elevated recombination-to-mutation ratio (r/m ≈ 1.70) is associated with reduced phylogenetic congruence of standard MLST loci, including *gyrB*, *groL*, and *recA*. In contrast, the *prc* gene exhibited the highest congruence with the recombination-filtered clonal genealogy (score = 0.94). Within the 95–96% ANI species boundary, the *prc* gene identity consistently remained more than 97%. This observed *prc* stability may be associated with functional constraints related to its predicted role in the periplasm. We therefore propose that a *prc* gene phylogeny and sequence identity threshold of > 97% could serve as a rapid and cost-effective marker for preliminary species-level assignment and epidemiological surveillance of *Aeromonas* spp., particularly in settings where a whole-genome sequencing is not readily available.

**Supplementary Information:**

The online version contains supplementary material available at https://doi.org/10.1038/s41598-026-67534-6.

## Introduction

The genus *Aeromonas* comprises diverse Gram-negative bacteria that are ubiquitous in aquatic environments^1^. These oxidase-positive bacilli are widespread in aquatic systems, ranging from wastewater to intensive aquaculture environments, where they act as primary pathogens for fish species such as Nile tilapia (*Oreochromis niloticus*) and European seabass^2–4^.

Species such as *A. hydrophila*, *A. salmonicida*, and *A. veronii* are the etiological agents of motile aeromonad septicemia and furunculosis, that cause catastrophic mortality in fish populations and severe financial losses for producers worldwide^5^. The virulence of *Aeromonas* species is modulated by a complex interplay of host, environmental, and intrinsic genetic factors^3^.

Despite their impact, *Aeromonas* spp. taxonomy is extremely complex^1,6^. *Aeromonas* taxonomy has undergone extensive revision since the late 19th century. Originally, it was classified within the genus *Bacillus*, and was later established as a distinct genus following refinements in phenotypic and molecular diagnostics^4,6^. The first *Aeromonas* species were described in 1891 as *Bacillus hydrophillus fuscus* (now *A. hydrophila*) from diseased frogs. Subsequently, in 1894, *Bacillus salmonicida* (now *A. salmonicida*) was isolated from trout, and its genus was formally named by Stanier in 1943^4,7^. In the 1970s, species were classified into mesophilic (motile, non-pigmented *A. hydrophila* at 37 °C, linked to human infections) and psychrophilic (non-motile, pigmented *A. salmonicida* at 22–28 °C, fish pathogens) groups based on growth temperature, motility, and pigment production^7^.

*Aeromonas* has undergone significant taxonomic expansion, with the number of valid species growing to 14 by 1998, accompanied by the establishment of the family Aeromonadaceae^8^. Subsequent studies through 2022 confirmed this ongoing diversification, describing the genus as comprising more than 30 Gram-negative bacterial species, primarily aquatic inhabitants associated with fish, animals, and human opportunistic infections such as gastroenteritis, wound infections, and septicemia^9^.

Earlier characterizations in the 1980–2000s focused on key genomospecies like *A. hydrophila*, *A. caviae*, *A. sobria*, *A. veronii*, *A. jandaei*, *A. schubertii*, and *A. trota*, with collections of 147–268 strains revealing species-specific biochemical traits and distributions across clinical, environmental, and animal sources^10–12^.

Species identification traditionally relies on biochemical profiling; however, high phenotypic plasticity often results in overlapping the metabolic signatures, leading to frequent misidentification^13,14^. Furthermore, 16S rRNA gene sequencing lacks the resolution to differentiate closely related species. The central driver of this confusion is the high rate of horizontal gene transfer (HGT)^15^.

Comprehensive reviews up to 2010 and Bergey’s Manual entries affirm the genus’s phylogenetic stability within Gammaproteobacteria, with over 30 species noted by 2022, but highlight challenges in species-level identification via methods like MALDI-TOF MS, which succeeds at genus level (> 95%) but varies for specific taxa like *A. bestiarum* and *A. hydrophila*^16–18^.

Commonly used characterization methods for *Aeromonas* isolates from aquaculture and aquatic environments include 16S rRNA gene sequencing for initial identification, Multi-locus Sequence Typing (MLST) for genetic relatedness and strain differentiation, and Multi-Locus Phylogenetic Analysis (MLPA) for species confirmation, alongside biochemical tests and PCR-based assays^19–21^. These approaches have limitations, such as misidentification risks with phenotypic methods alone (biochemical tests failing to distinguish *A. hydrophila* accurately)^14,22^, low resolution in diverse populations, and incomplete databases hindering sequence type assignments in MLST^23^. Genome-wide approaches, including Average Nucleotide Identity (ANI), in silico DNA-DNA hybridization (*is*DDH), and Whole-Genome Sequencing (WGS), serve as gold standards for precise taxonomy and phylogenomics but each has its own drawbacks of being resource-intensive and limited by database gaps^23,24^.

MLST is a commonly employed molecular method for genotyping and phylogenetic analysis of *Aeromonas* species, particularly in studies of isolates from aquatic environments, fish farms, and clinical samples, enabling the identification of sequence types (STs) and assessment of genetic diversity^24^.

The standard housekeeping genes used as markers in *Aeromonas* MLST schemes include *gyrB*, *groL* (also called *glnA* in some contexts), *gltA*,* metG*,* ppsA*, and *recA*, with concatenated sequences submitted to databases like PubMLST for ST assignments, whereas alternative or expanded schemes incorporate *rpoD*, *dnaJ*, *gyrA*, and *dnaX* for multi-locus phylogenetic analysis (MLPA)^25–27^.

Despite its utility in delineating clonal relationships and novel STs, MLST faces limitations such as reduced discriminatory power from single housekeeping gene alignments due to mutation and recombination events, potential misidentification of ambiguous strains requiring validation by whole-genome metrics like ANI/isDDH, and incomplete databases hindering full ST resolution^26–28^. The frequency of recombination in *Aeromonas* species exhibits notable interspecies variation. In the context of MLST individual genes, congruence analysis reveals significant taxa displacement, which disagree with the clonal genealogy. This observation raises concerns about the accuracy of its identification and the susceptibility of these genes to horizontal gene transfer (HGT). Furthermore, the MLST genes exhibit a high level of conservation among species, rendering them more susceptible to homologous recombination^26–29^.

For instance, in multidrug-resistant *A. veronii* strains isolated from freshwater fish farms, recombination analyses using RDP5 and ClonalFrameML identified 42 and 49 events, respectively (with 22 validated by multiple algorithms). These findings point out high recombination levels relative to mutations that limits single gene resolution and necessitates concatenated loci for accurate genotyping^27^.

Despite these attempts and advancements, WGS remains a cost- and resource-intensive approach for many laboratories in developing countries^30^, and the lack of a standardized *cg*MLST scheme opposes effective data comparison (https://www.cgmlst.org/). Consequently, there is an urgent need for a stable genetic marker that provides a high resolution analogous to that of whole-genome analysis but remains compatible with single-locus Sanger sequencing. This compatibility ensures that high-resolution species identification remains accessible for routine screening and epidemiological surveillance, particularly in resource-limited settings or standard diagnostic laboratories where whole-genome sequencing (WGS) is logistically or financially prohibitive for large sample sizes. In this study, we provide a phylogenomic revisit to *Aeromonas* type materials, quantify recombination rates across core genomes confounding traditional taxonomy, generate a clonal genealogy based phylogenetic tree and validate a novel marker that maintains topological stability even under the high recombination pressure characteristic of the genus.

## Materials and methods

### Data acquisition and quality filtration

For the 1st discovery stage, and to ensure high-resolution taxonomic identification within the genus *Aeromonas*, we utilized near-complete genomic datasets derived exclusively from type material. A total of 74 genomic assemblies were retrieved from the NCBI RefSeq database (accessed February 2026). To mitigate redundancy while maintaining a comprehensive representation of the genus, dereplication was performed using dRep (v. 3.6.2)^31^. All assemblies underwent rigorous quality curation; only type materials genomes assemblies exhibiting a completeness threshold of > 90% and a contamination limit of < 5%, as estimated by CheckM2 (v. 1.1.3)^32^ were retained for downstream analysis. This threshold was adopted to maximize taxonomic representation of the genus while maintaining a high standard of genomic integrity, as several *Aeromonas* type strain assemblies currently available in public repositories do not meet the stricter completeness criteria. Due to the redundancy of some strains, which are at different completion levels, dereplication was performed using dRep with a secondary ANI threshold of 95%. Due to the nature of the genus and some research suggested using the 95%- 96% cutoff value concept, distinct species clustering above 95% ANI were retained to preserve taxonomic diversity and integrity. In the validation stage, all *Aeromonas* assemblies marked as complete (*N* = 384) were retrieved. Following the same CheckM quality filtration (> 90% completeness, < 5% contamination), a final set of 374 high-quality genomes was retained for genus-wide marker vs. ANI regression analysis. The complete metadata and assemblies accession of the *Aeromonas* type materials and the complete genomes are listed in supplementary Tables S1 and S2 respectively.

### Average nucleotide identity (ANI) analysis

 To resolve taxonomic ambiguities arising from differently named strains within the same genomic clusters, we calculated pairwise ANI using the pyANI (v. 0.2.7) package^33^. Given the high genomic divergence within the genus *Aeromonas*, the ANIb method was specifically selected. This approach utilizes the BLAST+ algorithm to perform fragmented genomic alignments^34^ and is effective for *Aeromonas* strains comparison. The strains with pairwise ANI values greater than 95% are considered to belong to the same species. To supplement the ANI in cases where different species are clustered together, the Genome-to-Genome Distance Calculator online server (GGDC v3) was used to generate digital DNA-DNA hybridization (dDDH) values using the recommended BLAST+ method^35^. All genome sequences were subjected to pairwise comparisons against other sequences for those who cluster in the same cluster, but it has different names. Digital DNA-DNA hybridization (dDDH) values were calculated using formula 2, which is robust against variations in genome size and assembly fragmentation. Following established standards, a dDDH threshold of 70% was utilized for species delineation^36^.

### Annotation, pangenome and ortholog clustering

To ensure reproducibility and standardized nomenclature, all assemblies were functionally annotated using Bakta (v. 1.10.4)^37^. The annotation was executed using the Bakta full database (v. 6.0) with a 16-thread profile to ensure comprehensive protein identification and alignment with the latest standards.

Following the annotation, Bakta-generated GFF3 files were converted to the required GFF format for pangenome analysis. The pangenome was reconstructed using Roary (v. 3.13.0)^38^. To accommodate the significant interspecies divergence and genomic plasticity characteristic of the *Aeromonas* genus, the minimum BLASTp identity threshold for ortholog clustering was set to 70%. This permissive threshold was strategically selected to ensure the robust recovery of the core genome—specifically to prevent the exclusion of essential housekeeping loci such as *ppsA*, which exhibit high sequence variation across distant clades—while maintaining the integrity of orthologous group assignments. This parameter ensures that the identified 1,654 core genes represent a truly genus-wide conserved backbone rather than a subset biased toward high-identity species complexes. Genes present in 100% of the isolates were defined as the core genome. A high-resolution core gene alignment was subsequently generated using MAFFT (v. 7.490)^39^ for downstream phylogenomic reconstruction.

### Recombination aware core genome phylogenomic reconstruction

To resolve the evolutionary relationships among the *Aeromonas* type strains, a core genome phylogeny was reconstructed. The concatenated core gene alignment produced by Roary was utilized by FastTreeMP (v. 2.1.11)^40^ to infer an initial maximum likelihood (ML) tree under the Generalized Time Reversible (GTR) model of nucleotide substitution with gamma-distributed rate variation (-nt -gtr). Branch support was evaluated using SH-like local support values, with all major species-level nodes exhibiting values > 0.95, confirming the robustness of the reference clonal frame.

To account for the confounding effects of horizontal gene transfer (HGT) on phylogenetic topology, we employed ClonalFrameML (v. 1.13)^41^. This approach distinguishes between point mutations occurring along the vertical phylogeny and those introduced via recombination. The analysis utilized the initial ML tree and the core gene alignment to estimate key evolutionary parameters within a maximum likelihood framework: ratio of recombination to mutation (ρ/θ), the mean length of imported DNA fragments (δ), and the average divergence of imported DNA (ν). The relative impact of recombination compared to point mutation was expressed as the r/m ratio, calculated as *r/m = (ρ/θ)* x *δ* x *ν*. This metric quantifies the relative impact of recombination versus point mutation on sequence diversification.

### Selection and extraction of phylogenetic markers

To identify the optimal marker gene reflecting the vertical clonal evolution of the genus *Aeromonas*, we performed a genome-wide congruence analysis using the recombination aware core genome phylogeny as a reference. Nucleotide sequences of the 1,654 core orthologs were extracted using SeqKit (v. 2.1.0)^42^,, guided by the intersection of Bakta functional annotations and the Roary presence/absence matrix. Each gene was individually aligned using MAFFT (v. 7.490)^39^ with the --auto parameter to select the most appropriate alignment strategy based on sequence length and divergence. Following alignment, individual maximum likelihood (ML) gene trees were constructed using FastTreeMP (v. 2.1.11)^40^ under the GTR model of nucleotide substitution (-nt -gtr). Branch support was evaluated using SH-like local support values, with all major species-level nodes exhibiting values > 0.95, confirming the robustness of the reference clonal frame.

Phylogenetic congruence between each individual gene tree and the ClonalFrameML-derived reference tree was quantified using the Symmetric Difference (Robinson-Foulds, RF) distance. The analysis was implemented in R (v. 4.2.3) using the phytools (v. 2.5-2)^43^ and ape (v. 5.8)^44^ packages. To enable direct comparisons across datasets, the RF distance was normalized to a congruence score ranging from 0.0 to 1.0 (where 1.0 indicates perfect topological identity) using the formula:

$\:Congruence=1-\frac{RF\left({T}_{\mathrm{gene}},{T}_{\mathrm{ref}}\right)}{2n-6}$Where $\:RF\left({T}_{\mathrm{gene}},{T}_{\mathrm{ref}}\right)$ represents the Robinson-Foulds distance between individual core gene tree (*T*_*gene*_)and the ClonalFrameML reference tree (T_ref_), and * n* is the total number of taxa (*n* = 22). The resulting scores were ranked in descending order to identify candidate markers that precisely recapitulated the vertical species phylogeny while exhibiting the lowest sensitivity to horizontal genetic exchange.

### Phylogenetic congruence and topological stability

To evaluate the diagnostic reliability of the candidate markers, a congruence analysis performed by comparing individual gene trees against the recombination-filtered reference phylogeny was used. Global topological congruence for the top marker and the concatenated MLST scheme was quantified using the Symmetric Difference (Robinson-Foulds, RF) distance using the same tools used in previous section^45^. To ensure statistical validity, all trees were unrooted and transformed into strictly bifurcating topologies before deriving a congruence score, where 1.0 represents perfect topological identity. To compare the branch lengths and to resolve localized phylogenetic instability within the genus, per-taxon Cophenetic Correlation Coefficients were calculated. For each strain, a patristic distance matrix was generated from the gene-specific tree and correlated against the corresponding matrix from the reference clonal frame. The branch length and lineage stability were defined using a stringent correlation threshold of 0.85, where strains maintaining a coefficient ≥ 0.85 were classified as stable, representing conserved vertical inheritance, while those falling below the threshold were identified as phylogenetically unstable^46^. The resulting phylogenies were visualized as tanglegrams with a dual-color mapping strategy.

### Codon-aware sequence alignment and evolutionary selection analysis

To accurately compare the evolutionary trajectories of the *prc* marker against traditional MLST housekeeping genes, a codon-aware alignment was performed for all 22 *Aeromonas* type strains. Nucleotide sequences were first aligned based on their corresponding amino acid translations using TranslatorX (v. 1.1)^47^. This approach ensures the maintenance of the triplet codon structure and prevents the introduction of gap-driven frameshift artifacts, which are critical for accurate downstream analyses of selection pressure. The evolutionary selection pressure acting on each locus was quantified by calculating the ratio of non-synonymous (dN) to synonymous (dS) substitution rates (ω = dN/dS). Global and site-specific ω values were estimated using the Single-Likelihood Ancestor Counting (SLAC) method implemented in the HyPhy (v. 2.0) software package^48^.

A GTR nucleotide substitution model was fitted to the data to derive branch lengths and substitution biases. Site-specific selection was determined using a binomial-based statistical test with a significance threshold of *p* < 0.1. To ensure robustness against ancestral state reconstruction uncertainty, 100 ancestral sequence samples were generated to obtain confidence intervals and median values for site-level inferences. Loci with ω < 1 were classified as being under purifying (negative) selection, indicating functional conservation, while ω > 1 indicated diversifying (positive) selection.

### Evaluation of *prc* gene as a species-level marker

To evaluate the taxonomic resolution of the *prc* gene, comparative analysis against whole-genome Average Nucleotide Identity (ANI) was performed. Genus-wide assemblies *Aeromonas* were subjected to the same quality criteria used previously. Total of 374 Genomic assemblies for fulfilled the quality check and processed using skani (v. 0.3.1)^49^ to generate an all-vs-all ANI matrix in a reasonable time compared to pyANI used with the limited number of type strains at the discovery stage. Simultaneously, the *prc* sequences were extracted from the 374 assemblies using local BLASTn (v. 2.12.0+) with the *A. hydrophila* ATCC 7966 *prc* locus (2,534,701–2,536,713, length = 2012 bp) as the query with default parameter against of custom database created using makeblastdb function using the tabulated output format (-outfmt “6 sseqid sstart send”) and the sequences were extracted into multi-fasta format. All sequences were aligned using Clustal Omega (clustalo --threads = 8 --force) and manually inspected/visualized to guarantee that no truncated genes or assembly artifacts corrupted the alignment. The pairwise genetic distances for the *prc* gene were calculated using clustalo (v. 1.2.4), and distances (d) were converted to percentage identity using the formula % Identity = (1 - d) x 100. Data integration and visualization were performed in R using the tidyverse (v. 2.0.0) and ggpubr (v. 0.6.0) packages. To ensure the integrity of the regression model, we applied a Residual Interquartile Range (IQR) filtering method. Initial linear regression was performed to calculate residuals; data points with residuals exceeding 1.5 IQR were classified as outliers and removed.

## Results

### Data acquisition and quality filtration

A total of 74 genomic assemblies, representing 35 *Aeromonas* species, were initially retrieved. The dataset was dominated by *A. salmonicida* (*N* = 8) and *A. hydrophila* (*N* = 6), followed by *A. caviae* and *A. media* (*N* = 5 each). Twenty other species were involved, each was represented by a single genome, including *A. allosaccharophila*,* A. simiae*, and *A. aquatica*. The dataset encompassed varying assembly levels, spanning from fragmented draft contigs and scaffolds to complete genome. Most genomes were at the scaffold level (*N* = 36), followed by contigs (*N* = 24), complete genomes (*N* = 12), and chromosome-level assemblies (*N* = 2). Genomic architecture varied significantly across the genus; the mean genome size was 4.54 ± 0.26 Mbp, ranging from 3.89 Mbp *(A. cavernicola* DSM 24474) to 5.25 Mbp (*A. rivipollensis* DSM 24593). Correspondingly, the mean gene count was 4,269 ± 238, with a range of 3,725 to 4,972 genes (Supplementary Table S1). The curation process involved a two-stage filtration strategy. Initial dereplication via dRep identified 26 representative genomic clusters. Seven of these assemblies were subsequently excluded for failing to meet the priori CheckM quality thresholds (Completeness > 90%, Contamination < 5%). To ensure a comprehensive representation of the genus diversity, three additional genomes *A. mytilicola* subsp. aquatica A8 and, *A. ichthyocola* A5, were manually retained. Although these strains clustered with other species at a FastANI similarity > 95%, they met all quality criteria and were preserved. Similarly, *A. oralensis* AB005 was included to maintain taxonomic integrity despite its proximity to *A. hydrophila*. The final curated set comprised 22 high-quality *Aeromonas* type strains (Table 1), providing a diverse and robust framework for subsequent functional and comparative genomic profiling. Full results for the dRep secondary clusters are shown in Supplementary Material Fig. S1. Full quality control of assemblies and judgment are noted in the additional material Table S3.

**Table 1:** List of dereplicated and quality checked passed genomes along with the completeness and contamination levels.

Type strain	Genome completeness	Genome contamination	Accession number
*A australiensis* CECT 8023^T^	94.17	0	GCF_000819725.1
*A bestiarum* CCUG 41,859 ^T^	90.15*	0	GCF_042656725.1
*A caviae* NCTC 12,244 ^T^	99.97	0	GCF_900476005.1
*A dhakensis* CIP 107,500 ^T^	95.38	0	GCF_000820305.1
*A diversa* CDC 2478-85 ^T^	90.85*	0	GCF_000819805.1
*A encheleia* NCTC12917 ^T^	99.91	0	GCF_900637545.1
*A enteropelogenes* FDAARGOS 1509 ^T^	100	0	GCF_020097315.1
*A eucrenophila* CCUG 30,340 ^T^	97.81	0	GCF_042656765.1
*A hydrophila* sub *hydrophila* ATCC 7966 ^T^	99.97	0	GCF_000014805.1
*A jandaei* FDAARGOS 986 ^T^	100	0.29	GCF_016127195.1
*A lusitana* MDC 2473 ^T^	91.61	0	GCF_002812985.1
*A media* CCM 3653 ^T^	99.97	1.17	GCF_042647745.1
*A rivipollensis* DSM 24,593 ^T^	99.97	0	GCF_046226845.1
*A rivuli* DSM 22,539 ^T^	90.05*	0	GCF_000820045.1
*A salmonicida* NCIMB1102 ^T^	100	0	GCF_963425675.1
*A schubertii* CECT 4240 ^T^	94.07	0	GCF_000820105.1
*A sobria* CIP 74.33 ^T^	95.09	0	GCF_018359945.1
*A taiwanensis* LMG 24,683 ^T^	92.01	1.72	GCF_000699185.1
*A veronii* CECT 4486 ^T^	92.32	0	GCF_000820365.1
*A mytilicola* sub *aquatica* A8 ^T^	99.97	0.58	GCF_044336385.1
*A ichthyocola* A5 ^T^	93.96	0.63	GCF_046087985.1
*A oralensis* AB005 ^T^	98.89	0	GCF_049665575.1

*Strains near the 90% threshold were retained as they represent essential type materials for distinct species clades that would otherwise be missing from the analysis. ^T^ = Type strains.

### Average nucleotide identity (ANI) analysis

**Fig. 1:**
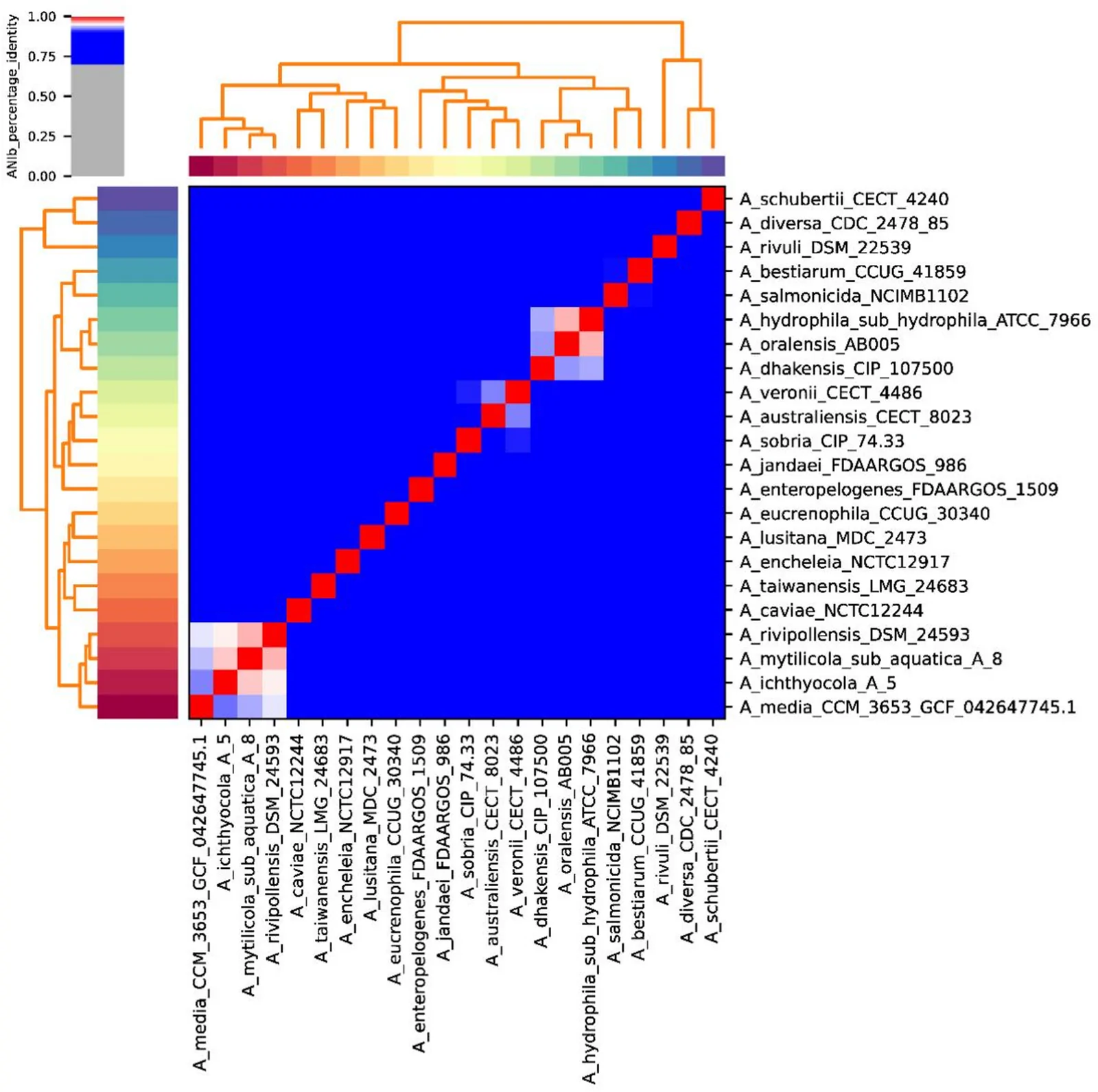
Heatmap and hierarchical clustering of Average Nucleotide Identity (ANIb) values among 22 *Aeromonas* type strains. The matrix represents the pairwise identity percentages calculated using the pyANI package with the BLAST+ algorithm. The color scale indicates the level of genomic relatedness, ranging from blue (lower identity) to red (100% identity). The hierarchical clustering (dendrograms) illustrates the phylogenetic relationships within the genus.

Pairwise genomic relatedness was assessed using the ANIb method to define species boundaries across the 22 *Aeromonas* type strains (Fig. 1). While an ANI threshold of 95–96% is traditionally used to denote conspecificity, our analysis revealed several instances of taxonomic ambiguity. The majority of the matrix consists of blue-to-purple regions, confirming that most strains in the dataset are distinct species sharing < 95% ANI. However, specific clusters exhibited values exceeding this threshold. Notably, *A. oralensis* AB005 and *A. hydrophila* subsp. *hydrophila* ATCC 7966 shared an ANI value of 96.24%, which would typically suggest that they belong to the same species. Nevertheless, the calculated dDDH value was 68.9%, falling just below the 70% threshold required for species delineation. A similar conflict was observed within the *A. ichthyocola* A5, *A. mytilicola* A8, and *A. rivipollensis* cluster; while ANI values consistently exceeded 95%, the interspecies dDDH values remained below 70%. The complete list of conflicting values is listed in Table 2. These discrepancies highlight the fuzzy species borders within *Aeromonas* previously characterized by Diop et al.^50^. In contrast, species such as *A. rivuli*, *A. schubertii*, and *A. diversa* appeared significantly more genetically divergent, forming distinct outgroups in the hierarchical clustering. The dendrogram associated with the ANIb heatmap further reinforced the close genomic relationships within the *A. media* group. Collectively, these results emphasize that while ANIb provides a broad taxonomic overview, the presence of high-identity interspecies necessitates the use of high-resolution phylogenomic markers for precise identification, specifically in aquaculture-related pathogens.

**Table 2:** Genomic resolving power in *Aeromonas* species complexes.

Query strain	Reference strain	ANIb (%)	dDDH (Formula 2, %)
*A. oralensis* AB005 ^T^	*A. hydrophila* ATCC 7966 ^T^	96.24	68.9
*A. ichthyocola* A5 ^T^	*A. mytilicola* A8 ^T^	95.73	64.2
*A. ichthyocola* A5 ^T^	*A. rivipollensis* DSM 24,593 ^T^	95.12	62.8
*A. mytilicola* A8 ^T^	*A. rivipollensis* DSM 24,593 ^T^	95.45	63.5

### Annotation, pangenome and ortholog clustering

Pangenome analysis of the 22 *Aeromonas* type strains revealed a remarkably expansive and fluid genomic architecture. Utilizing a 70% sequence identity threshold for ortholog clustering, a total of 14,316 distinct orthologous groups were identified. The hard-core genome (genes present in 100% of isolates) consisted of 1,654 genes, representing approximately 38.7% of the average individual *Aeromonas* genome (mean gene count = 4,269).

The soft core contained 229 genes, The accessory genome—comprising the shell (15% to 99% occupancy) and cloud (present in < 15% of isolates) components—accounted for the vast majority of the pangenome, with 12,433 identified gene clusters (86.8% of total orthologous groups). Specifically, the cloud genome was the most expansive fraction, containing 9,125 genes, many of which were unique to single species. This significant accessory component, coupled with the relatively small core-to-pangenome ratio, underscores the significant genetic plasticity and the open nature of the *Aeromonas* pangenome. Such a high level of variation is likely driven by persistent HGT and specialized adaptations to the diverse ecological niches occupied by members of this genus.

### Recombination aware core genome phylogenomic reconstruction

ClonalFrameML analysis estimated recombination frequency relative to mutation (ρ/θ) of 0.142 and a mean DNA import length (δ) of 214 bp. The average divergence of these imported DNA fragments (ν) was 0.0558. The resulting r/m ratio of ≈ 1.70 demonstrates that recombination is the dominant driver of polymorphism in the *Aeromonas* core genome. The distribution of recombination events was not uniform across the genus; for instance, *A. rivuli* DSM 22,539 showed the highest intensity (0.167), followed by *A. sobria* CIP 74.33 (0.115), and *A*. *enteropelogenes* FDAARGOS 1509^T^ (0.110) representing highly recombinogenic lineages and the respective species appear to be hotspots for genetic exchange within the genus. Conversely, species like *A. rivipollensis*, *A. mytilicola* and *A. ichthyocola* exhibited the lowest recombination intensities which were as low as 0.005. Moreover, *A. oralensis* AB005 (0.009) and *A. diversa* CDC 2478-85 (0.015) exhibited low recombination intensities, suggesting all these lineages have evolved more clonally within the genus. These findings suggest that while the *Aeromonas* core genome remains stable, HGT events significantly influence the evolutionary trajectory and diversification of individual species lineages.

### Selection and extraction of phylogenetic markers

The genome-wide comparative analysis of 1654 core genes revealed substantial variation in the ability of individual loci to recapitulate the reference clonal genealogy. While many core genes exhibited topological distortions due to the high genus-wide recombination rate (r/m = 1.70), a subset of markers demonstrated exceptional stability. Table 3 summarizes the top 10 core genes ranked by their phylogenetic congruence. The genes *prc* (encoding a C-terminal processing protease) and *sunT* (an ABC transporter/maturation protease) emerged as the most reliable markers. Both loci achieved a congruence score of 0.9473 and the lowest observed Symmetric Difference (SD) distance of 2 across the 22 *Aeromonas* type strains analyzed. All top 10 candidates exhibited high congruence (> 0.89), indicating that the evolutionary signal within these specific core genes remains remarkably resilient against horizontal genetic exchange. The high performance of these loci, particularly *prc*, uncovers them as promising alternatives to traditional housekeeping genes for multi-locus Sequence Analysis (MLSA) or as standalone diagnostic markers for resolving complex *Aeromonas* species boundaries.

**Table 3:** Phylogenetic congruence metrics for the top 10 core genes with core genome phylogeny. The ranking is based on the highest Congruence values and lowest SD Distance to the reference species tree across 22 *Aeromonas* strains.

No#	Gene	Symmetric difference	Congruence score
1.	prc	2	0.947
2.	*sunT*	2	0.947
3.	*bamC*	4	0.895
4.	*corC*	4	0.895
5.	*fliF*	4	0.895
6.	*hpt*	4	0.895
7.	*lptF*	4	0.895
8.	*mltC*	4	0.895
9.	*panF*	4	0.895
10.	*ppiD*	4	0.895

In 19 out of the 22 analyzed strains, the *prc* phylogeny provided a better fit to the reference phylogenetic tree than MLST. Notably, for *A. enteropelogenes* FDAARGOS 1509^T^, the alternative markers achieved a stability score of 0.908, compared to 0.715 for the MLST set.

### Phylogenetic congruence and topological stability

**Fig. 2:**
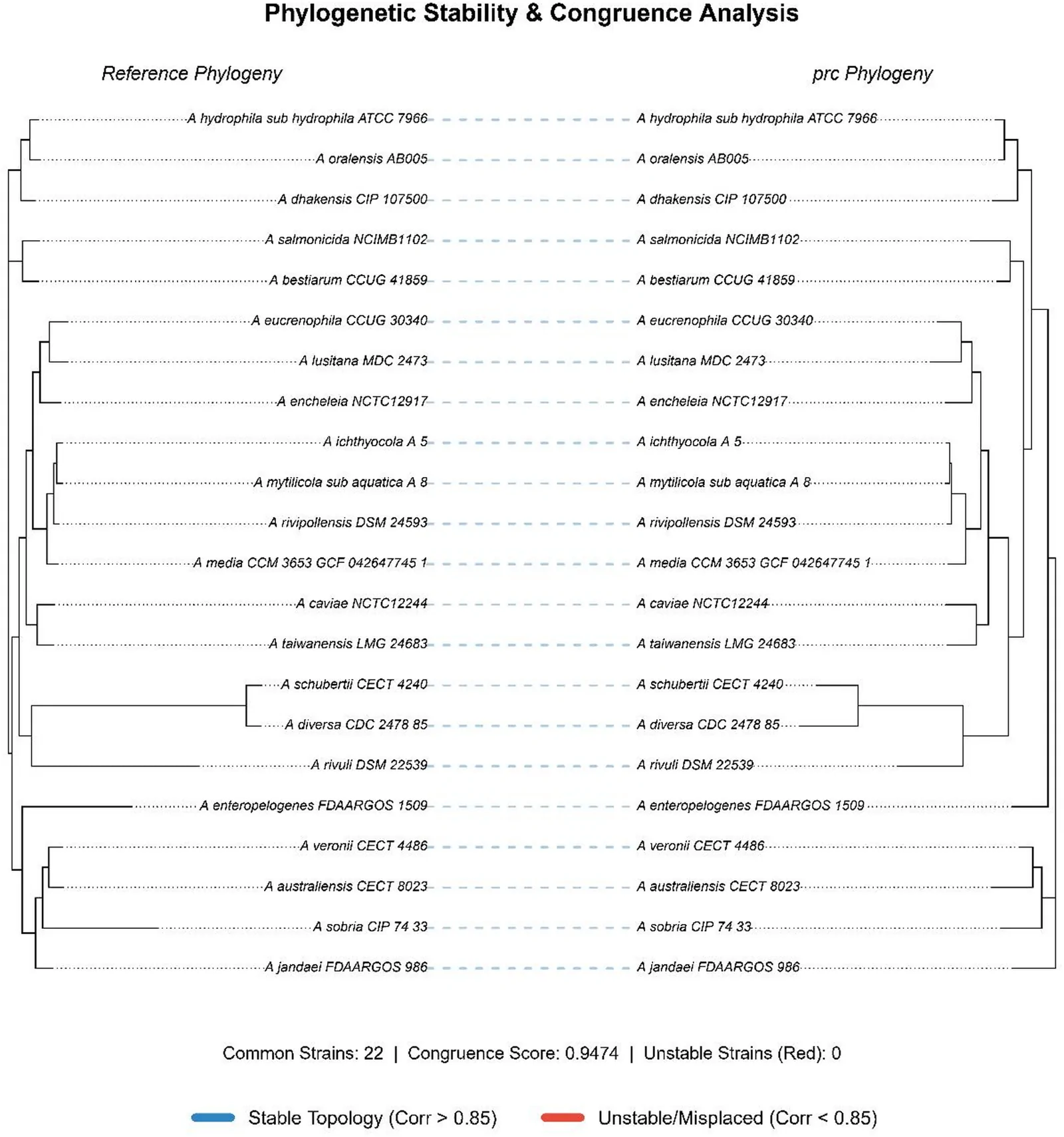
Comparative phylogenetic congruence between the clonal genealogy and the suggested novel *prc* marker. Tanglegram analysis illustrates the near-perfect congruence between the recombination-filtered core genome reference tree (left) and the *prc* phylogeny (right). Straight blue lines denote high congruence, confirming that the *prc* marker accurately reflects the vertical evolutionary history of the 22 *Aeromonas* strains without the distorting influence of recombination.

**Fig. 3:**
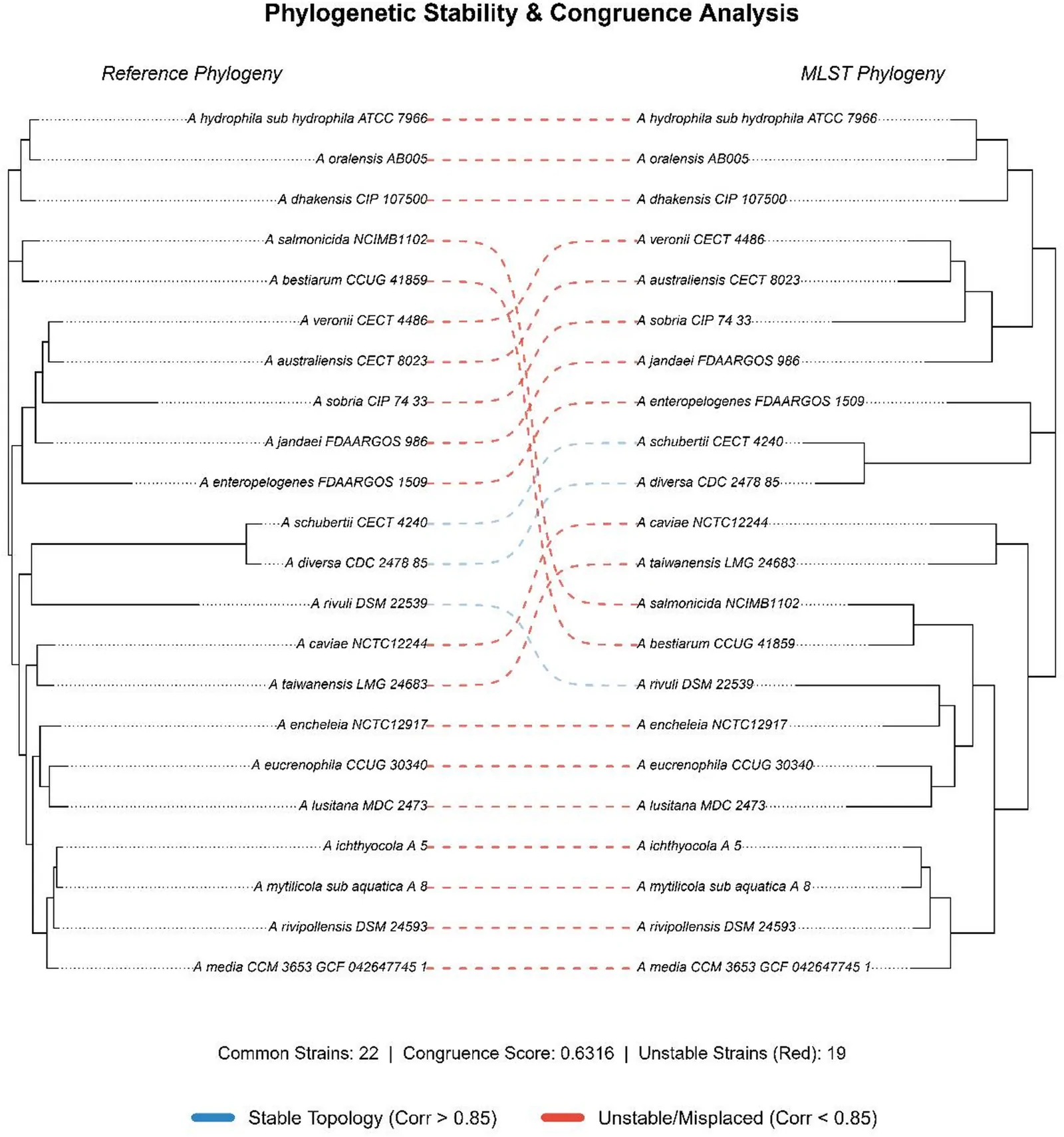
Comparative phylogenetic congruence between the clonal genealogy and the traditional MLST scheme. Tanglegram analysis illustrates the significant topological instability between the recombination-filtered core genome reference tree (left) and the 6-gene MLST phylogeny (right). The extensive crossing of trajectories (red lines) indicates an incongruence rate of 86%, highlighting the limitations of standard MLST in capturing the vertical evolutionary history of *Aeromonas* under high recombination pressure.

The topological robustness of the suggested novel *prc* marker (region: 2,534,701–2,536,713 of accession CP000462) was evaluated via tanglegram analysis against the recombination-filtered clonal genealogy (Fig. 2). The analysis revealed an exceptional degree of phylogenetic congruence, with the *prc*-based phylogeny achieving near-perfect congruence (RF = 2) across all the 22 *Aeromonas* type strains. As illustrated in Fig. 2, every individual lineage is connected by parallel blue trajectories, indicating that the branching patterns and clade memberships of the *prc* tree perfectly mirror the vertical evolutionary history of the core genome. The total absence of intersecting red lines confirms that the *prc* locus is highly resistant to the confounding effects of HGT and homologous recombination, remaining strictly aligned with the clonal frame of the genus. In contrast, the traditional MLST scheme exhibited profound topological instability when measured against the same clonal genealogy (Fig. 3). Tanglegram analysis revealed extensive phylogenetic conflict, with 19 out of 22 *Aeromonas* strains (86%) displaying unstable trajectories. As shown in Fig. 3, the numerous crossed red lines indicate significant shuffling of taxa between the reference standard and the MLST-based phylogeny. The 3-way comparison between *prc*-based trees and traditional MLST and the reference tree is shown in Table 4.

This high degree of incongruence suggests that the concatenated signal from the six housekeeping genes (*gyrB*,* recA*,* groL*,* gltA*,* metG*, and *ppsA*) fails to accurately represent the vertical evolutionary history of the genus. These results demonstrate that while traditional markers are subject to phylogenetic incongruence driven by high recombination rates, the *prc* gene provides a stable and highly congruent phylogenetic signal. Individual congruence scores for each MLST gene are summarized in Table 5, with corresponding tanglegrams provided in Supplementary Figure S2.

**Table 4:** Three-way comparison of *prc*, MLST and reference phylogeny.

Pair	Congruence
MLST vs. Reference tree	0.632
*prc*-based tree vs. Reference tree	0.947
*prc*-based tree vs. MLST	0.579

**Table 5:** Comparative selection pressure and phylogenetic congruence of *prc* and MLST individual marker genes against the reference phylogeny.

Marker	Global dN/dS (ω)	Congruence score	Selection type (*p* < 0.1)
*groL*	0.0473	0.5789	Negative
*ppsA*	0.0610	0.7368	Negative
*gltA*	0.0637	0.5789	Negative
*gyrB*	0.0699	0.3684	Negative
*recA*	0.0775	0.4737	Negative
*metG*	0.0874	0.3684	Negative
*prc*	0.0876	0.9474	Negative

### Codon-aware sequence alignment and evolutionary analysis

The comparative analysis of evolutionary selection pressures revealed that the *prc* marker and all traditional MLST loci are under strong purifying (negative) selection (Table 5). The global dN/dS ratios for all markers were significantly below the 0.1 threshold, ranging from 0.0473 (*groL*) to 0.0876 (*prc*). Among the standard MLST markers, *groL* exhibited the highest level of conservation (ω = 0.0473), followed by *ppsA* (0.0610) and *gltA* (0.0637). The *prc* marker demonstrated a global ω of 0.0876, which, although slightly higher than the MLST set, still indicates strong purifying selection. Notably, the extent of conservation did not correlate with phylogenetic reliability. Despite the extreme purifying selection acting on *groL* and *gyrB*, these genes exhibited the lowest congruence scores (0.5789 and 0.3684, respectively), reflecting a high susceptibility to recombination-driven topological distortion. Conversely, the *prc* gene effectively decoupled sequence conservation from phylogenetic stability, maintaining a congruence score of 0.9474 while retaining selective constraint sufficient to serve as a stable molecular clock.

### Evaluation of *prc* gene as potential species-level marker

**Fig. 4:**
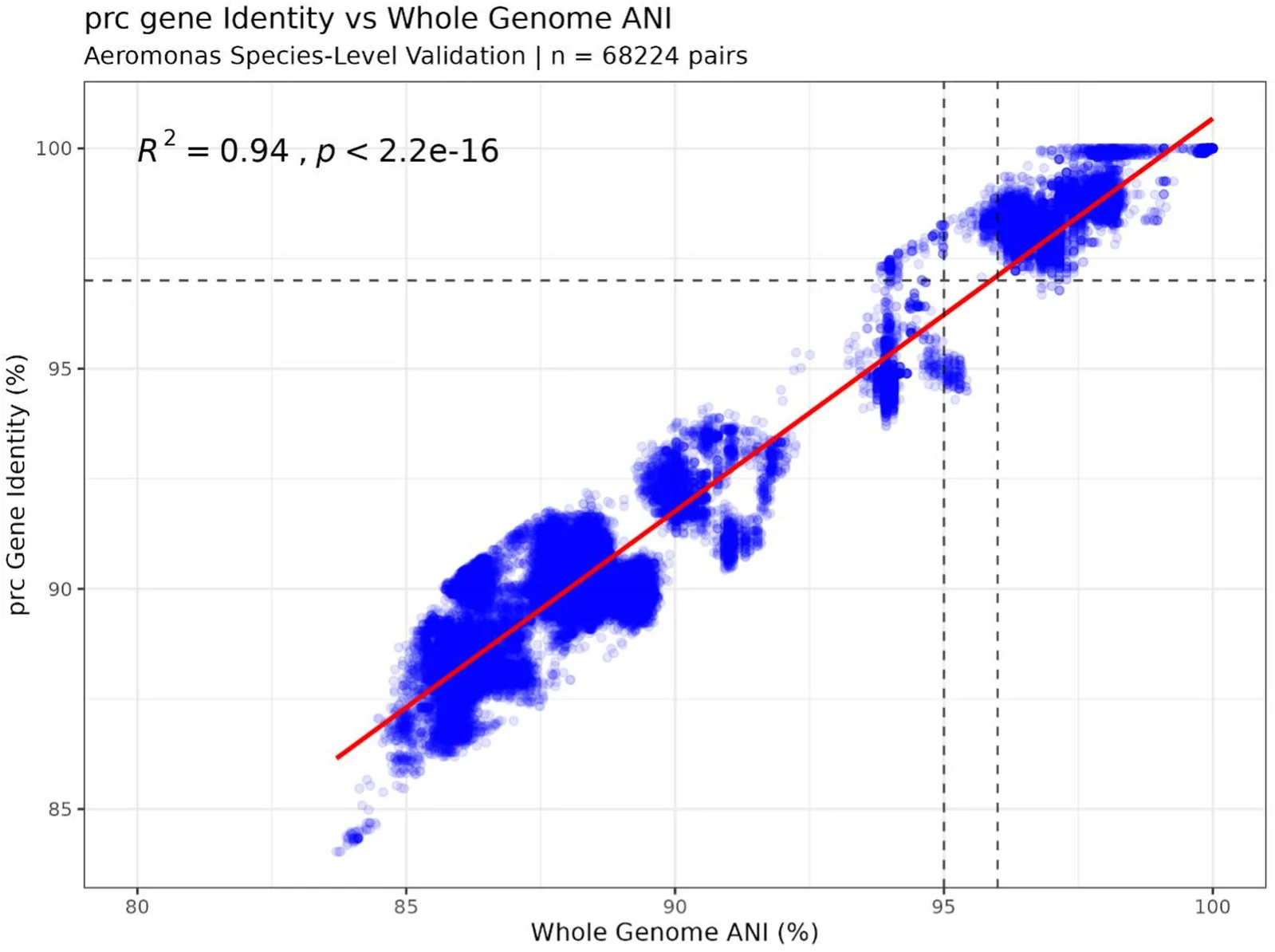
Genomic Congruence of the *prc* Taxonomic Marker: Regression analysis of *prc* gene identity versus whole-genome ANI for 374 *Aeromonas* genomes. Following the removal of 1524 statistical outliers, the dataset shows a strong linear correlation (R^2^ = 0.94), confirming the gene’s utility as a proxy for the core genome and a reliable tool for species-level identification. The two vertical lines represent the fuzzy species boundaries in *Aeromonas* while the horizontal line represents the 97% identity of the *prc* gene.

A total of 69,748 pairwise comparisons were initially generated. After applying the residual filtering, 1,524 outliers (approximately 2.1%) were removed, resulting in a cleaned dataset of 68,224 pairs. The Linear regression analysis result shown in Fig. 4 confirmed a robust correlation between *prc* sequence identity and whole-genome ANI (*R*^2^ = 0.94, *P* < 2.2 × 10^− 16^). The distribution of data points showed high density at both the species-level (ANI ≥ 95%) and the genus level (ANI 85–95%). Within the 95–96% ANI species boundary, the *prc* gene identity steadily remained above 97%, demonstrating its reliability as a high-resolution taxonomic marker. The regression trend line suggests that the *prc* gene evolves at a rate nearly proportional to the total core genome, with minimal saturation at higher genetic distances.

Despite the robust global correlation, 1,524 pairwise comparisons (2.1%) were identified as statistical outliers. These deviations were not stochastic but were concentrated within specific, highly recombinogenic lineages, most notably the *A. veronii* and *A. dhakensis* complexes. For instance, the significant positive residual observed between *A. veronii* ANYA_15694 and *A. sanarellii* NS1 suggests a localized introgression event of the *prc* locus. These findings serve as a critical cautionary note: while *prc* remains an exceptionally stable proxy for the genus, its application in known recombination hotspots like *A. veronii* should be interpreted alongside ecological or clinical metadata to account for rare horizontal exchange events. Importantly, a biostatistical audit of these 1,524 residual outliers ( 2.1% of 69,748 total pairs) revealed that these data points do not represent widespread, independent gene failures distributed across diverse taxa. Rather, they represent an inherent artifact of matrix compounding within an all-against-all comparison matrix (374 × 374) where a single aberrant assembly generates hundreds of pairwise outlier coordinates. Specifically, just two anomalous assemblies—*Aeromonas rivuli* 20_VB00005 (driving 313 outlier pairs; 20.5%) and *Aeromonas veronii* ANYA_15694 (driving 199 outlier pairs; 13.1%)—single-handedly account for over one-third (33.6%) of the entire outlier dataset. The remaining 97.9% of genus-wide genome pairs ($\:n=\mathrm{68,224}$) strictly conform to the linear model ($\:{R}^{2}=0.94,P<2.2\times\:{10}^{-16}$).

## Discussion

Although the number of type strain sequences in the database reached 74, most of these did not meet the quality criteria. In microbial genomics, the presence of low quality or highly contaminated genomes may result in overestimation of the accessory genome and false-positive HGT signals. By giving priority to chromosomal and high-quality assemblies, we have created and used high-quality datasets for *Aeromonas* type materials^50^.

The dereplication phase revealed a significant taxonomic challenge represented by the clustering of distinct species, such as *A. mytilicola* A8, *A. ichthyocola* A5, and *A. rivipollensis* at an ANI > 95%. The 95–96% ANI threshold is traditionally considered for species delimitation^51,52^. However, the preservation of these strains in our dataset, alongside the clustering of *A. oralensis* AB005 with *A. hydrophila*, highlights that a fixed ANI threshold may be insufficient for resolving species boundaries within the genus *Aeromonas*. This reinforces the recommendation that ANI values should be interpreted as a mathematical gradient reflecting ongoing evolutionary dynamics rather than a fixed, rigid cutoff. This observation supports the hypothesis of recombination-driven blurring within *Aeromonas*, wherein high rates of interspecies genetic exchange obscure the phylogenetic signals of standard housekeeping genes, leading to indistinct species boundaries.

When gene flow occurs between the core genomes of distinct bacterial species- a phenomenon known as introgression - blurred species boundaries can be created^50^. In the same study, researchers had to adjust the ANI thresholds (to be between 94% and 96%) for *Aeromonas* among other species, such as *Klebsiella*,* Pseudomonas*,* Vibrio and Yersinia* based on empirical data because they found that using strict 95% cutoffs may result in incorrectly over-splitting or merging distinct species within the genus. For *A. mytilicola* A8^T^ and *A. ichthyocola* A5^T^, which were classified as novel species based on the dDDH conducted by the authors where the values for dDDH were below the 70% threshold for species delineation^53^. However, the reported ANI values were 95.73% and 94.29% (using BLAST) and 95.95% and 95.11% (using MUMmer)^53^. This example clearly illustrates the taxonomic ambiguity and blurred species boundaries described by Diop et al.^50^. For *A. oralensis* AB005, it was identified initially as *A. hydrophila* based on the 16S rRNA and *gyrB* gene sequences by Mashzhan et al. (2025). The biochemical profile of this strain showed that it does not produce indole and tests negative for ornithine decarboxylase and D-xylose fermentation.

Despite the limitations of using 16S rRNA and *gyrB* as molecular markers for species identification, the OrthoANI value based on the WGS against the *A. hydrophila* type strain was 96.38% above the upper boundary of the fuzzy interval proposed by Diop et al.^50^ for species like *Aeromonas*. Despite these ANI values in both cases, the dDDH showed values less than 70% which is also rechecked in this work. These cases underscore the inherent limitations of traditional MLST and ANI cutoffs in *Aeromonas* and emphasize the urgent need for a more reliable, stable marker that can accurately reflect vertical evolution amidst high recombination such as *prc* proposed by this work.

The dominance of the accessory genome, specifically the large cloud fraction (9,125 genes) is a hallmark of an open pangenome^54^. In *Aeromonas*, this fluidity is likely driven by the genus’s high capacity for natural transformation and conjugation, allowing for the rapid acquisition of virulence factors, antibiotic resistance genes, and metabolic pathways essential for survival in aquatic environments^55,14^.

The quantification of the r/m ratio as 1.70 indicates that recombination is the dominant evolutionary force over mutation within the *Aeromonas* genus, with a likelihood of introducing nucleotide substitutions that exceed the rate of point mutation. An r/m > 1 suggests that the vertical inheritance of the genome is under constant modification by the horizontal acquisition of genetic material^41^. The high intensity observed in lineages such as *A. rivuli* (0.167) and *A. sobria* (0.115) identifies these species as genomic hotspots for genetic exchange. These species often occupy highly dynamic aquatic interfaces, where HGT is maybe a requisite for survival against shifting environmental stressors, such as fluctuating salinity or antibiotic pressure^56^. From a systematic perspective, an r/m ratio of 1.70 poses a substantial challenge to traditional taxonomy. When recombination beats mutation, the phylogenetic signal preserved in individual housekeeping genes can become unclear, leading to topological incongruence.

Our findings regarding the r/m ratio of 1.70 are directly corroborated by the specific distribution of outliers. The high recombination intensity previously noted in the *A. rivuli* and *A. sobria* complexes manifests as a high frequency of topological noise in our regression analysis. We identified *A. rivuli* 20_VB00005 as a significant recombination hotspot, as it accounted for over 20% of the total outliers detected. Furthermore, the case of *A. veronii* ANYA_15694 illustrates a divergent allele, where the *prc* locus has been replaced by a sequence from a distant relative, leading to a negative residual of -11.3 despite a high genomic ANI (98.39%). Similar cases were reported in in *Phaeobacter inhibens* for antibiotic synthesis and detoxification, *Alteromonas macleodii* for exopolysaccharide production, and *Marinobacter hydrocarbonoclasticus* for hydrocarbon degradation, and also for cold adaptation in *Pseudoalteromonas haloplanktis*^57^.

The concentration of outliers in specific lineages like *A. rivuli* and *A. veronii* reinforces the status of these taxa as genomic ‘hotspots’ for genetic exchange. However, the fact that these events affect only 2.1% of genus-wide comparisons—compared to the 86% incongruence observed in traditional MLST—highlights that the *prc* gene is uniquely resilient. These rare introgression events likely represent the upper limit of HGT tolerance for a gene so deeply integrated into the periplasmic network, further supporting its role as a high-fidelity anchor for the vertical clonal frame.

The genome-wide congruence analysis provides a quantitative shift in our understanding of *Aeromonas* systematics. While the majority of the 1,654 core genes exhibit significant topological conflict, which is likely due to the high interspecies recombination rate (r/m = 1.70), the *prc* and *sunT* genes demonstrate near-perfect congruence (0.947) with clonal genealogy. An SD distance of 2 in a 22-taxon tree is statistically remarkable; it implies that these loci have remained virtually untouched by the HGT events that have impacted the phylogenetic signal in other core regions.

The striking contrast in topological stability between the novel *prc* marker and the traditional MLST scheme provides definitive evidence of the phylogenetic erosion affecting standard *Aeromonas* diagnostics. While the *prc*-based phylogeny achieved 100% lineage consistency with the recombination-filtered clonal genealogy, the concatenated MLST framework failed to accurately represent the vertical history of 86% of the analyzed strains. This profound instability suggests that the current reliance on housekeeping genes like *gyrB*, *recA*, and *groL* is fundamentally compromised by the high rates of HGT.

The tangling observed in the MLST tanglegram is a direct consequence of the genus-wide r/m ratio of 1.70. In highly recombinogenic taxa, housekeeping genes often serve as hotspots for localized genomic introgression, where alleles are frequently swapped between distinct species during environmental or clinical co-habitation^50,58^. When these mixed signals are concatenated into an MLST scheme, the resulting phylogeny does not represent a real evolutionary lineage but rather a mosaic of conflicting histories. This explains why traditional MLST often struggles to resolve the fuzzy species boundaries of the *A. hydrophila* and *A. media* complexes, frequently leading to the misidentification of serious aquaculture pathogens.

In contrast, the perfect topological stability of the *prc* gene indicates that it is protected from the widespread recombination noise of the *Aeromonas* pangenome. From an evolutionary perspective, the *prc* gene (encoding a C-terminal processing protease) is predicted to be involved in critical cell envelope maintenance and protein maturation. Genes involved in such essential cellular processes are often subject to intense purifying selection, as any non-synonymous change could disrupt essential protein-protein interactions within the periplasm, potentially imposing substantial fitness costs^59–61^. This functional constraint may contribute to preserving the vertical signal of the *prc* locus, allowing its use as a marker that protects the phylogenetic signal and tracks the true species phylogeny (the clonal frame) even when the rest of the genome is severely affected by a high recombination rate.

Although both the traditional MLST loci and the *prc* marker were evolutionarily conserved, *prc* showed substantially greater congruence with the recombination-filtered clonal genealogy. This observation suggests that factors other than sequence conservation, such as differential susceptibility to homologous recombination, play an important role in determining the phylogenetic utility of a marker. The failure of the most conserved housekeeping genes (*groL*, *ppsA*, *gltA*) to recapitulate the clonal frame can be explained by the mechanics of homologous recombination. Highly conserved sequences act as a suitable candidate for the cell’s recombination machinery^62^. This makes gold standard markers like *gyrB* and *recA* highly susceptible to interspecies swapping, effectively mixing their phylogenetic signals in highly recombinogenic environments.

The observed stability of the *prc* gene is consistent with a relatively constrained evolutionary role. With a dN/dS of 0.0876, it is under enough purifying selection to maintain its functional integrity and structural stability. However, its slightly higher dN/dS compared to *groL* suggests that it has accumulated a higher density of synonymous substitutions. This subtle sequence divergence may act as a recombination barrier, making the locus less likely to be recognized and swapped by the homologous recombination machinery of distantly related *Aeromonas* species. Consequently, the *prc* locus appears to be less susceptible to homologous recombination and therefore better retains the vertical phylogenetic signal, allowing it to track the true speciation history. However, other more conserved genes are susceptible to horizontal gene transfer. These findings challenge a central tenet of microbial systematics - that maximal sequence conservation equates to optimal phylogenetic utility - by demonstrating that highly conserved genes can be more susceptible to homologous recombination.

While a comprehensive evaluation of local genomic synteny and structural biochemistry is beyond the scope of this study, functional association mapping via the STRING database reveals that the protein product of the *prc* gene (Prc) operates as a high-connectivity hub within the periplasmic network. The predicted Prc interacts critically with the NlpI lipoprotein adapter and murein synthases (MrcA/PBP1a and MrcB/PBP1b) to maintain cell wall envelope integrity. In accordance with the Complexity Hypothesis^59,60^, we hypothesize that the evolutionary stability of the *prc* locus is maintained by severe co-adaptation constraints. An incoming horizontal variant would likely lack the precise molecular fit required to interface with multiple native protein partners simultaneously, imposing a substantial metabolic or structural fitness penalty. This functional constraint creates a biological barrier to horizontal gene transfer, effectively preserving the vertical phylogenetic signal across the genus.

Although the initial tanglegram analysis was based on 22 carefully curated, dereplicated type strains, *prc* maintained a strong linear relationship with whole-genome ANI across the complete 374-genome dataset (69,748 pairwise comparisons; R² = 0.94), indicating that its phylogenetic signal extends well beyond the discovery set. Moreover, among all loci examined individually, *prc* exhibited the highest congruence with the clonal-frame reference phylogeny, exceeding the congruence scores of all MLST housekeeping genes. These findings suggest that *prc* is a promising proxy for core-genome relatedness within the genus *Aeromonas* and is consistent with predominantly vertical inheritance. The results highlight the potential of *prc* for species-level identification when the urgency of identification is a must. Specifically, at the 95–96% ANI threshold the *prc* gene provides clear separation between taxa. While WGS remains the current gold standard for accurate species identification, via this research, the *prc* marker stands out as a promising candidate for rapid, preliminary species identification where genomic infrastructure is limited or not available.

Although *prc* demonstrated high phylogenetic congruence and high taxonomic potential in this study, it may not resolve all *Aeromonas* lineages or rare recombination events. Further empirical validation using diverse biological isolates from different environments and geographical locations is therefore needed to assess its potential for accurate isolate discrimination. Therefore, ambiguous cases should be confirmed using whole-genome analyses or additional genetic markers.

## Conclusion

The evolutionary history of the genus *Aeromonas* has long been obscured by the phylogenetic noise caused by HGT. The current study demonstrates that the current 6-gene MLST standard remains useful for typing, however, fails to resolve the true vertical inheritance of type strains, largely due to the high recombination to mutation ratio (r/m = 1.70) affecting the core genome. By applying a recombination aware phylogenomic framework, this study identified the *prc* gene as a promising phylogenetic marker with a near-perfect genus genealogy congruence (score ~ 0.95). Compared to conventional MLST, *prc* provides a higher-resolution frame into the clonal evolution of *Aeromonas*, while a sequence identity threshold of > 97% emerged as a reliable proxy for the 95% ANI species boundary. The greater phylogenetic stability of *prc* relative to commonly used housekeeping genes such as *gyrB* and *recA* suggests that *prc* may better preserve the vertical evolutionary signal.

Finally, these findings support the use of *prc* as a practical single-locus marker for species-level identification and surveillance of *Aeromonas* spp., potentially reducing the computational and financial demands of large-scale genomic monitoring while maintaining close concordance with whole-genome phylogeny.

## Supplementary Information

Below is the link to the electronic supplementary material.


Supplementary Material 1.


## Data Availability

The datasets used in the study are accessible from the NCBI, and complete information, including the accession number, BioProject, BioSample and Assembly Accession, is included in the supplementary Table S1. Additionally, to ensure scientific reproducibility, the data and codes generated during this study have been made publicly available in the GitHub repository at: https://github.com/baabdella/Aeromonas-prc-marker. Any additional information requested is available upon request from the corresponding author.
