# Supplementary material for: The prc gene as a high-resolution proxy for Aeromonas genus-wide clonal genealogy, phylogenomic validation and superiority over traditional MLST
Source: Sci Rep. 2026 Aug 27;16:26960. doi: 10.1038/s41598-026-67534-6 (PMC13522338; doi:10.1038/s41598-026-67534-6)
Supplement: Supplementary file 1 [file 41598_2026_67534_MOESM1_ESM.pdf]

Figure S1

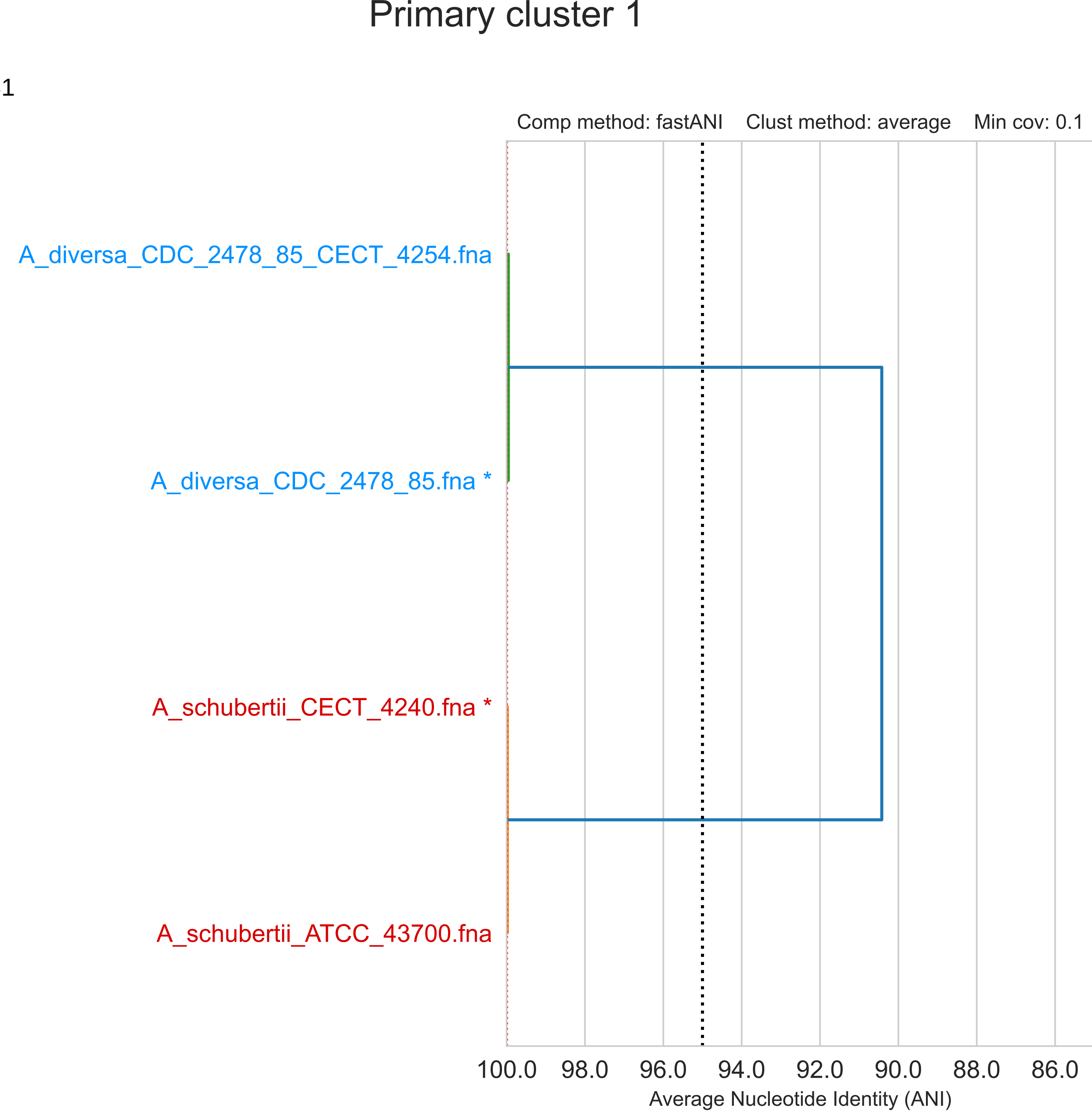
Primary cluster 1
Comp method: fastANI
Clust method: average
Min cov: 0.1
A_diversa_CDC_2478_85_CECT_4254.fna
A_diversa_CDC_2478_85.fna *
A_schubertii_CECT_4240.fna *
A_schubertii_ATCC_43700.fna
100.0
98.0
96.0
94.0
92.0
90.0
88.0
86.0
Average Nucleotide Identity (ANI)

Primary cluster 4

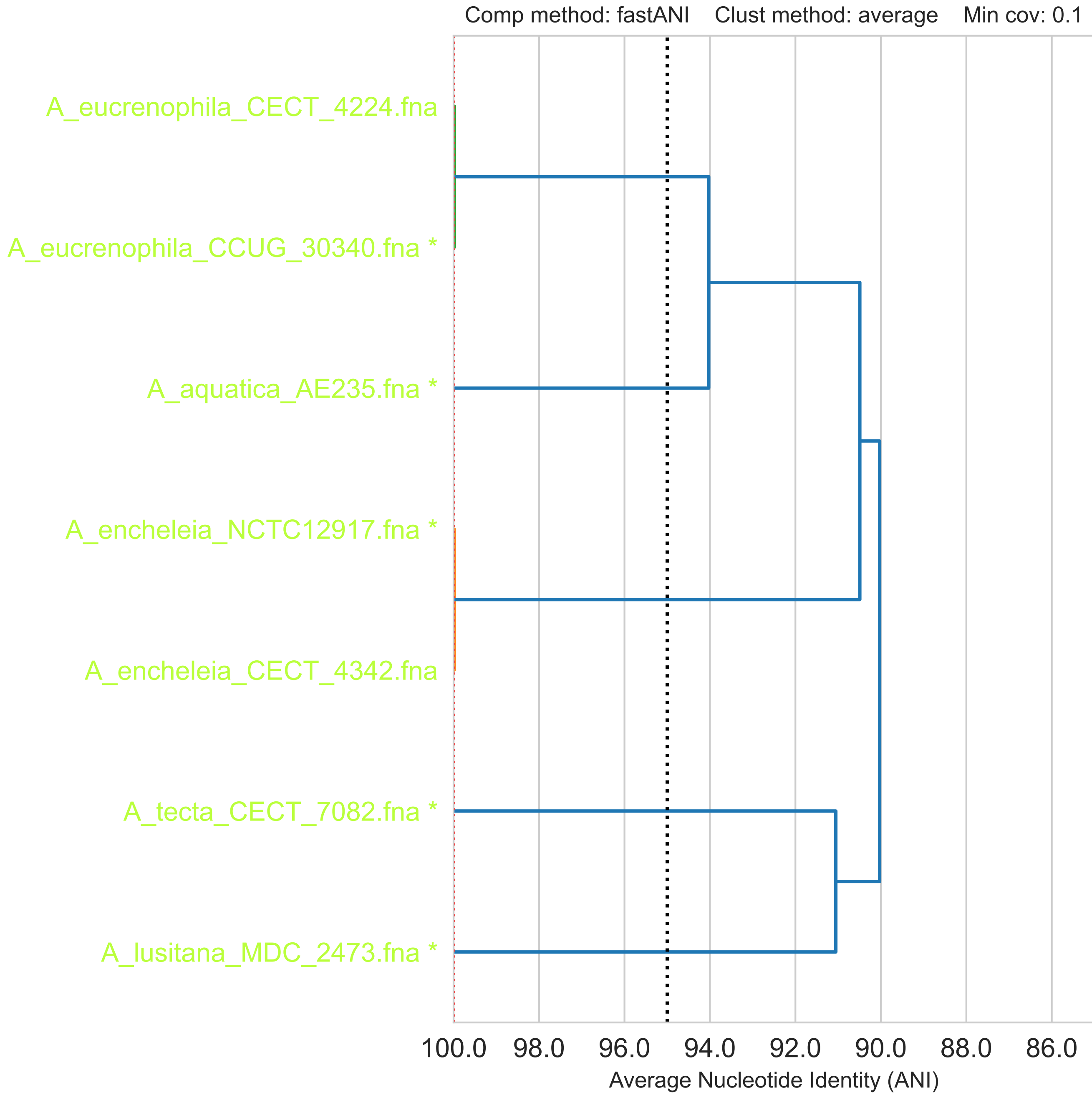

Primary cluster 5

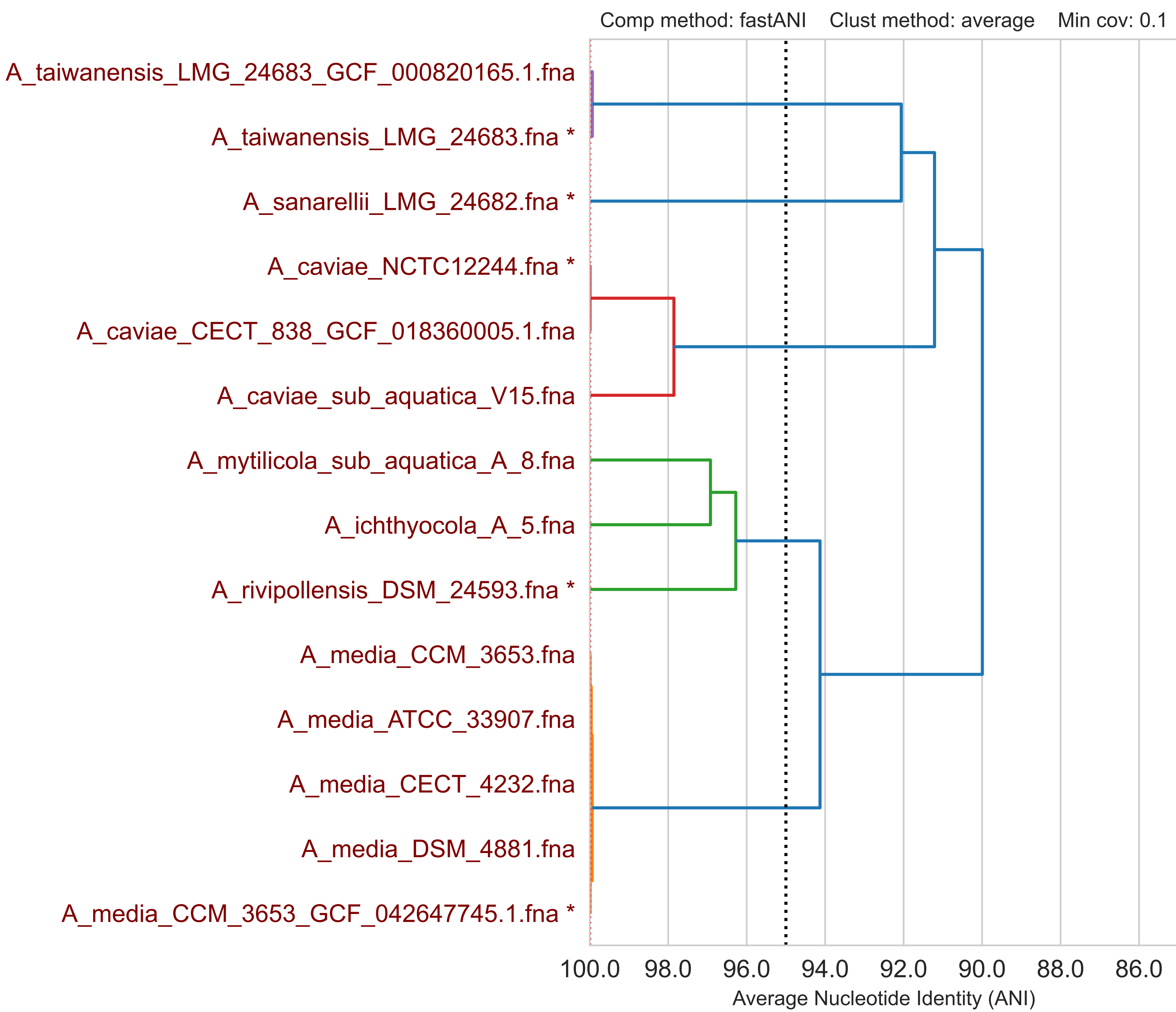

Primary cluster 6

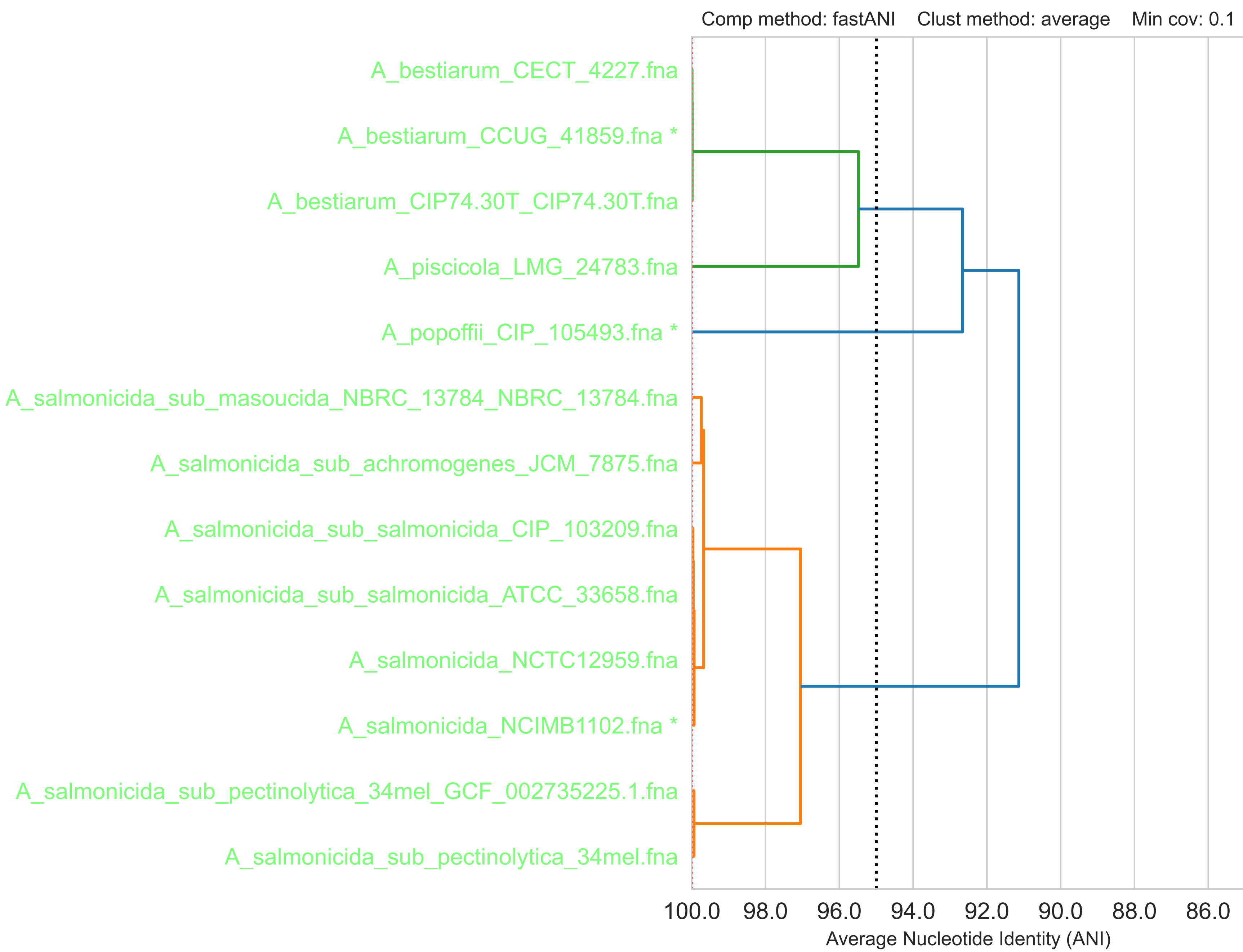

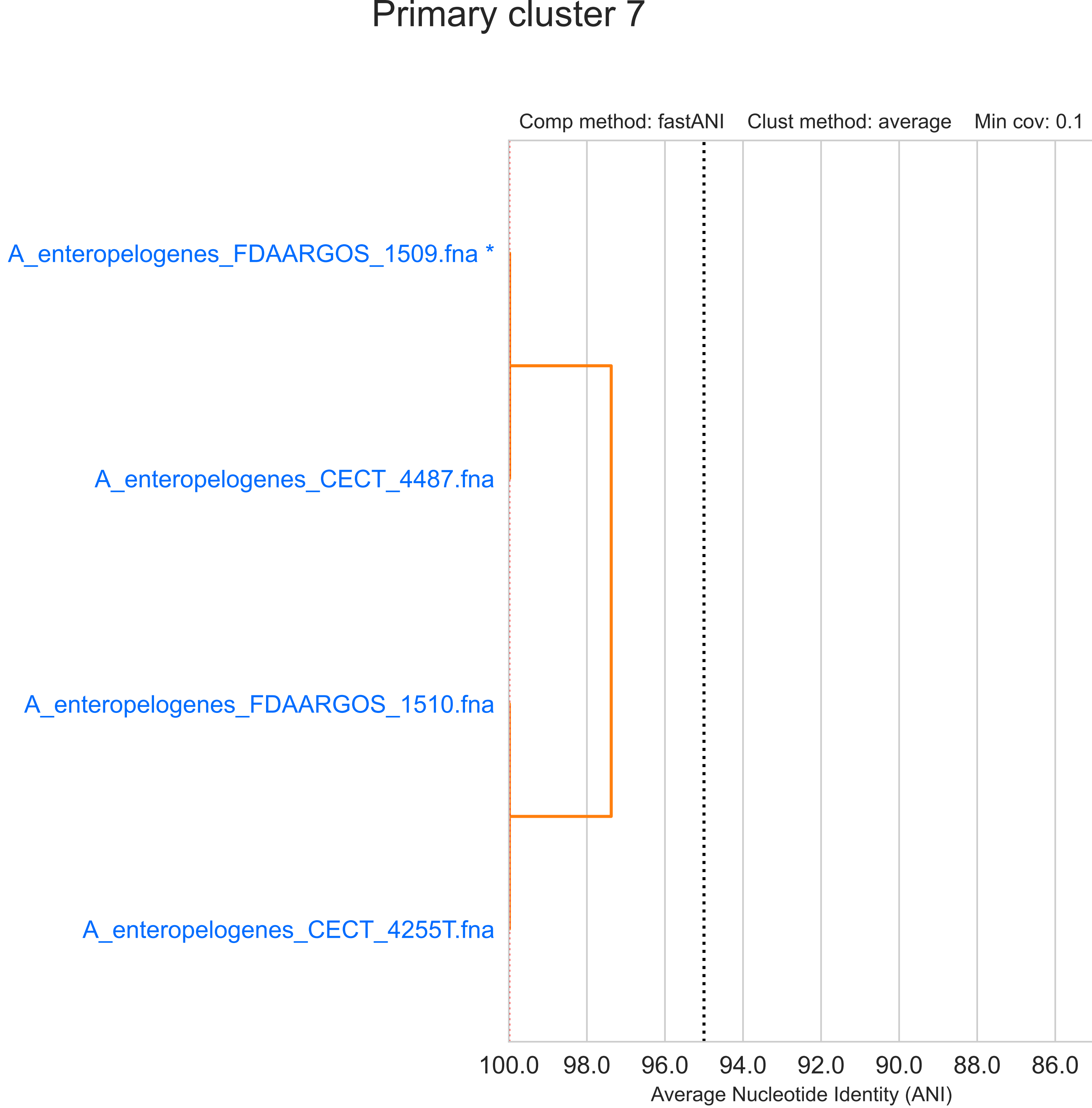

Primary cluster 7
Comp method: fastANI
Clust method: average
Min cov: 0.1
A_enteropelogenes_FDAARGOS_1509.fna *
A_enteropelogenes_CECT_4487.fna
A_enteropelogenes_FDAARGOS_1510.fna
A_enteropelogenes_CECT_4255T.fna
100.0
98.0
96.0
94.0
92.0
90.0
88.0
86.0
Average Nucleotide Identity (ANI)

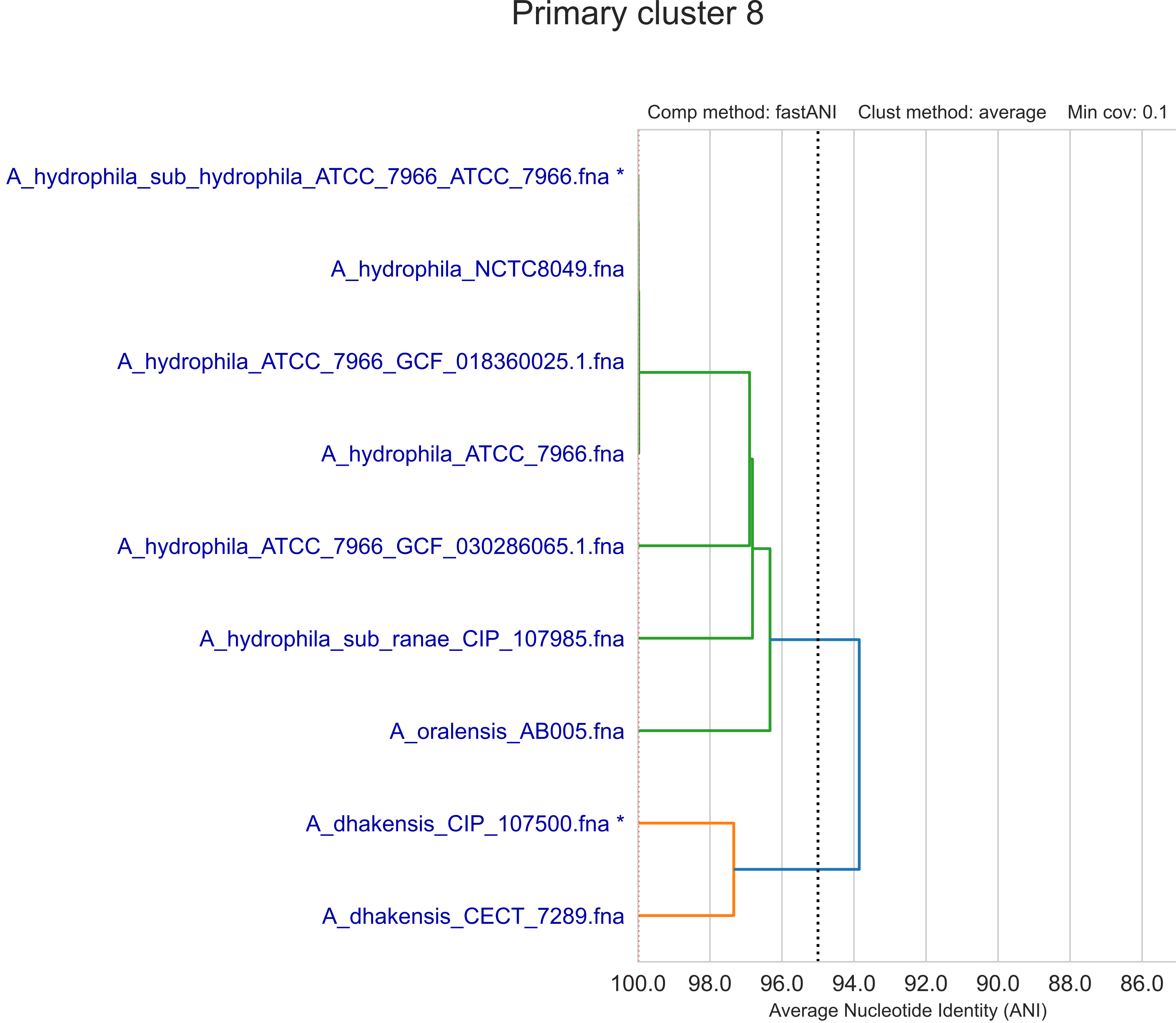
Primary cluster 8
Comp method: fastANI
Clust method: average
Min cov: 0.1
A_hydrophila_sub_hydrophila_ATCC_7966_ATCC_7966.fna *
A_hydrophila_NCTC8049.fna
A_hydrophila_ATCC_7966_GCF_018360025.1.fna
A_hydrophila_ATCC_7966.fna
A_hydrophila_ATCC_7966_GCF_030286065.1.fna
A_hydrophila_sub_ranae_CIP_107985.fna
A_oralensis_AB005.fna
A_dhakensis_CIP_107500.fna *
A_dhakensis_CECT_7289.fna
100.0
98.0
96.0
94.0
92.0
90.0
88.0
86.0
Average Nucleotide Identity (ANI)

Primary cluster 9

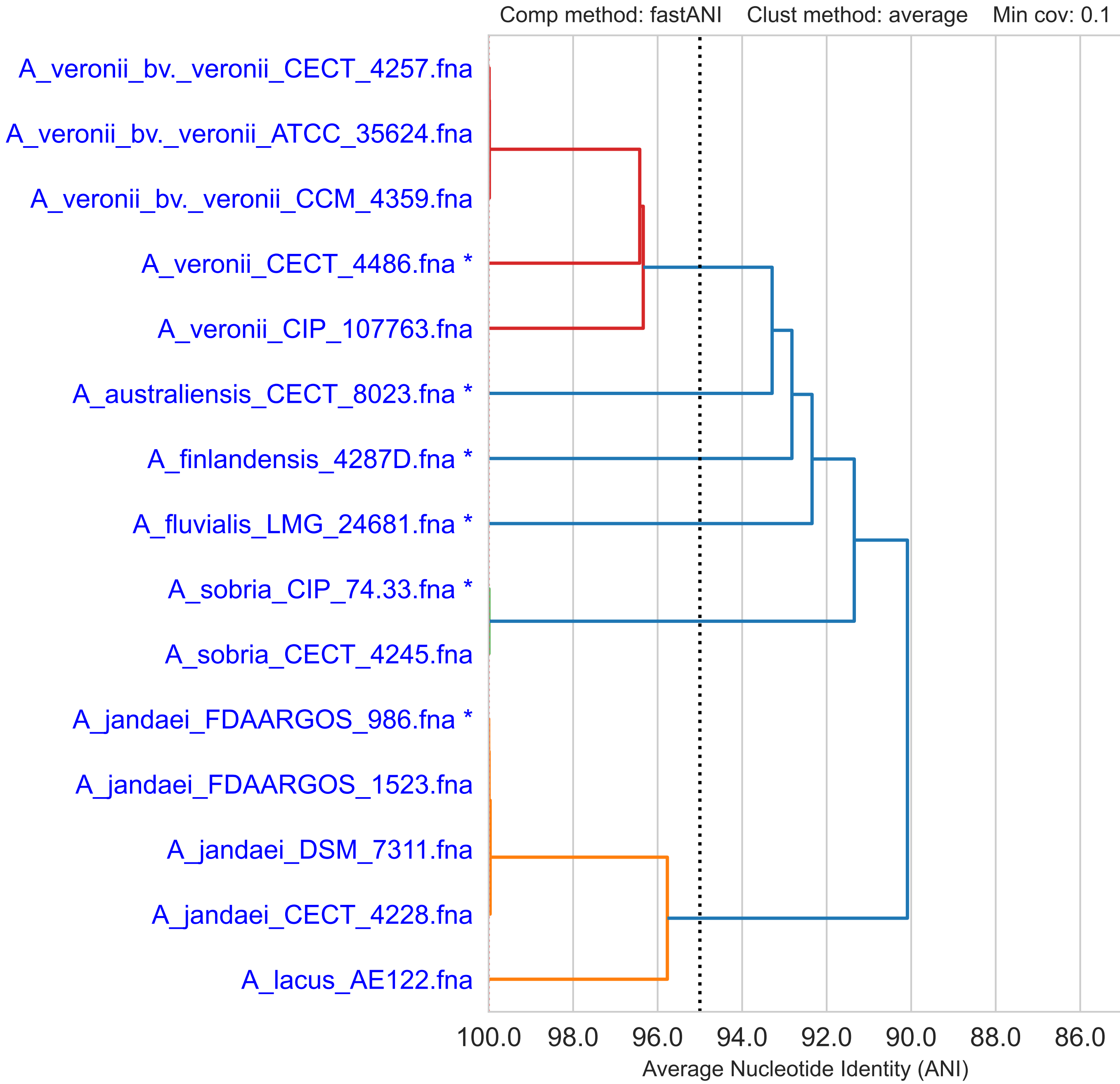

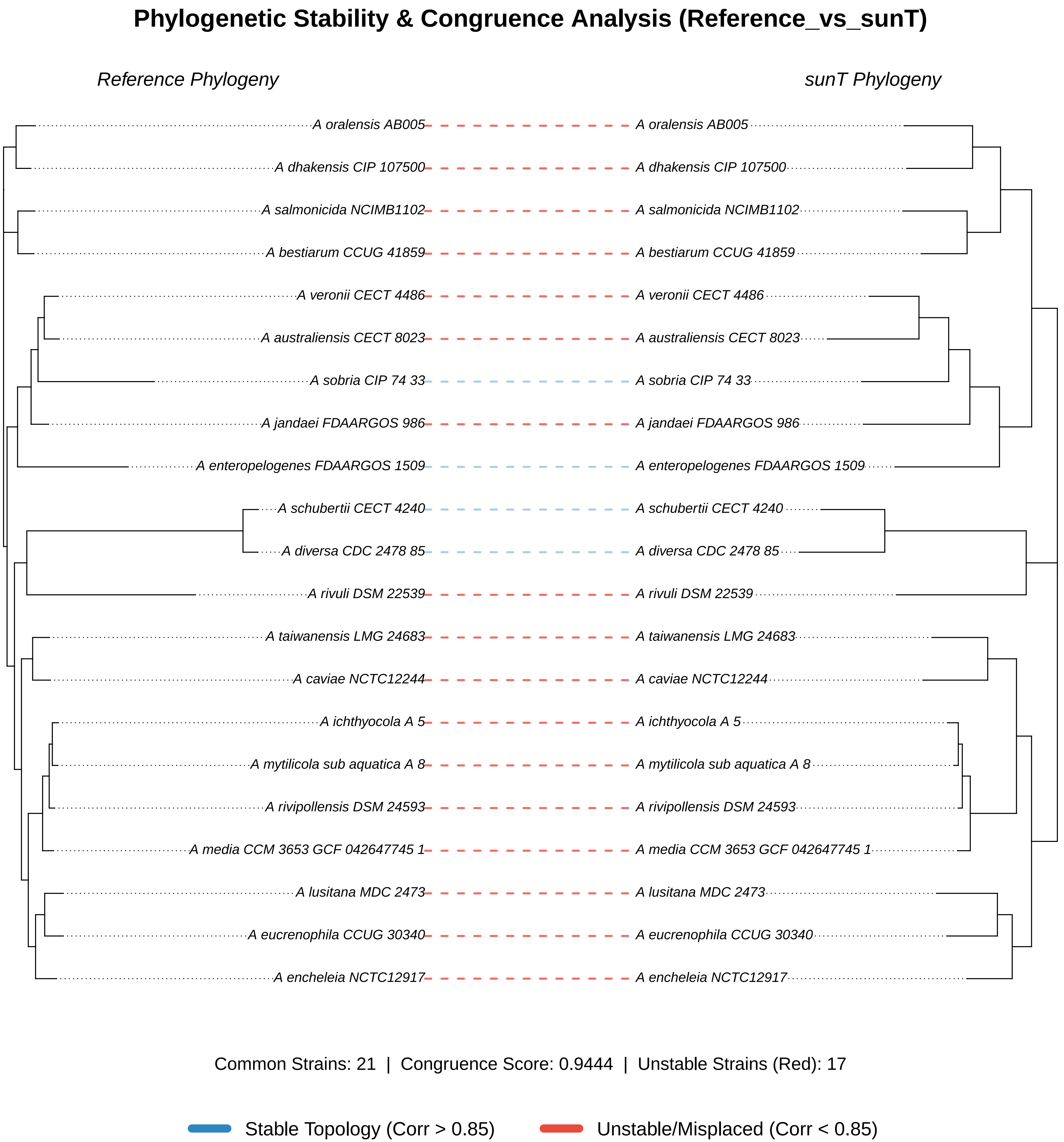
Phylogenetic Stability & Congruence Analysis (Reference_vs_sunT)
Reference Phylogeny
sunT Phylogeny
A oralensis AB005
A dhakensis CIP 107500
A salmonicida NCIMB1102
A bestiarum CCUG 41859
A veronii CECT 4486
A australiensis CECT 8023
A sobria CIP 74 33
A jandaei FDAARGOS 986
A enteropelogenes FDAARGOS 1509
A schubertii CECT 4240
A diversa CDC 2478 85
A rivuli DSM 22539
A taiwanensis LMG 24683
A caviae NCTC12244
A ichthyocola A 5
A mytilicola sub aquatica A 8
A rivipollensis DSM 24593
A media CCM 3653 GCF 042647745 1
A lusitana MDC 2473
A eucrenophila CCUG 30340
A encheleia NCTC12917
Common Strains: 21 | Congruence Score: 0.9444 | Unstable Strains (Red): 17
Stable Topology (Corr > 0.85)
Unstable/Misplaced (Corr < 0.85)

Phylogenetic Stability & Congruence Analysis (prc_vs_sunT)

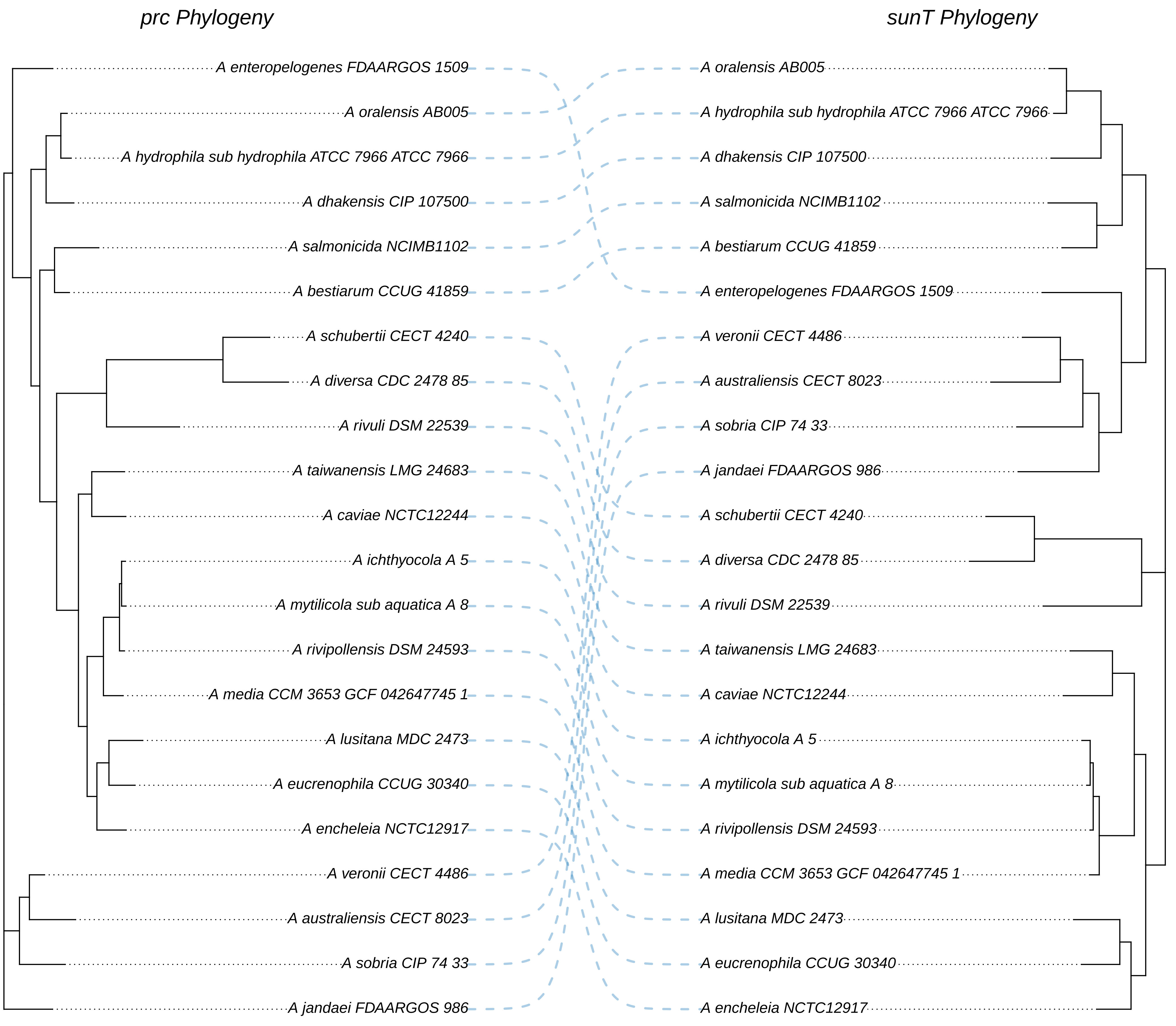

Common Strains: 22 | Congruence Score: 0.8947 | Unstable Strains (Red): 0

Stable Topology (Corr > 0.85)   Unstable/Misplaced (Corr < 0.85)

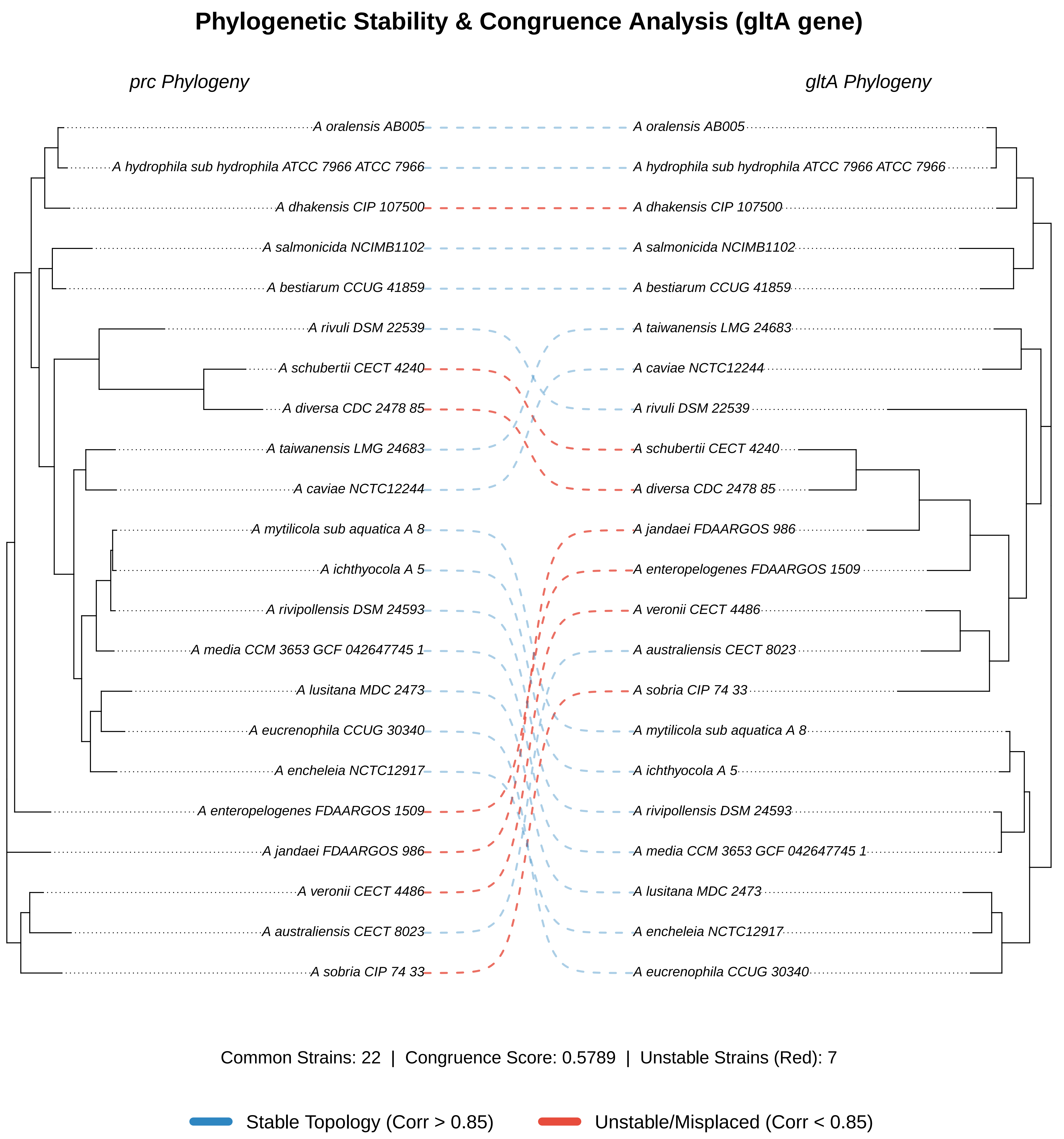
Phylogenetic Stability & Congruence Analysis (gltA gene)
prc Phylogeny
gltA Phylogeny
A oralensis AB005
A hydrophila sub hydrophila ATCC 7966 ATCC 7966
A dhakensis CIP 107500
A salmonicida NCIMB1102
A bestiarum CCUG 41859
A rivuli DSM 22539
A schubertii CECT 4240
A diversa CDC 2478 85
A taiwanensis LMG 24683
A caviae NCTC12244
A mytilicola sub aquatica A 8
A ichthyocola A 5
A rivipollensis DSM 24593
A media CCM 3653 GCF 042647745 1
A lusitana MDC 2473
A eucrenophila CCUG 30340
A encheleia NCTC12917
A enteropelogenes FDAARGOS 1509
A jandaei FDAARGOS 986
A veronii CECT 4486
A australiensis CECT 8023
A sobria CIP 74 33
Common Strains: 22 | Congruence Score: 0.5789 | Unstable Strains (Red): 7
Stable Topology (Corr > 0.85)
Unstable/Misplaced (Corr < 0.85)

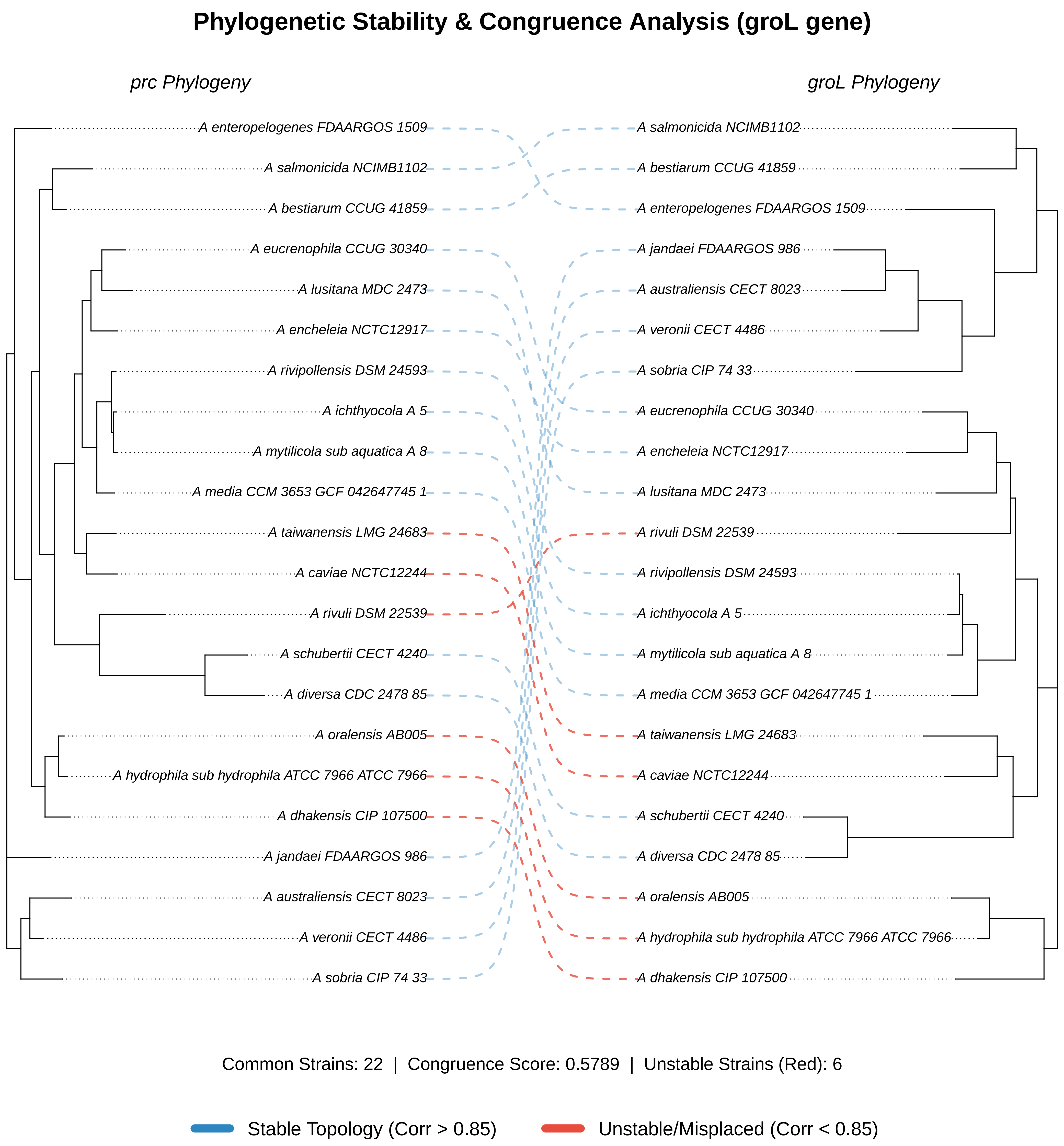

Phylogenetic Stability & Congruence Analysis (groL gene)
prc Phylogeny
groL Phylogeny
A enteropelogenes FDAARGOS 1509
A salmonicida NCIMB1102
A bestiarum CCUG 41859
A eucrenophila CCUG 30340
A lusitana MDC 2473
A encheleia NCTC12917
A rivipollensis DSM 24593
A ichthyocola A 5
A mytilicola sub aquatica A 8
A media CCM 3653 GCF 042647745 1
A taiwanensis LMG 24683
A caviae NCTC12244
A rivuli DSM 22539
A schubertii CECT 4240
A diversa CDC 2478 85
A oralensis AB005
A hydrophila sub hydrophila ATCC 7966 ATCC 7966
A dhakensis CIP 107500
A jandaei FDAARGOS 986
A australiensis CECT 8023
A veronii CECT 4486
A sobria CIP 74 33
A salmonicida NCIMB1102
A bestiarum CCUG 41859
A enteropelogenes FDAARGOS 1509
A jandaei FDAARGOS 986
A australiensis CECT 8023
A veronii CECT 4486
A sobria CIP 74 33
A eucrenophila CCUG 30340
A encheleia NCTC12917
A lusitana MDC 2473
A rivuli DSM 22539
A rivipollensis DSM 24593
A ichthyocola A 5
A mytilicola sub aquatica A 8
A media CCM 3653 GCF 042647745 1
A taiwanensis LMG 24683
A caviae NCTC12244
A schubertii CECT 4240
A diversa CDC 2478 85
A oralensis AB005
A hydrophila sub hydrophila ATCC 7966 ATCC 7966
A dhakensis CIP 107500
Common Strains: 22 | Congruence Score: 0.5789 | Unstable Strains (Red): 6
Stable Topology (Corr > 0.85)
Unstable/Misplaced (Corr < 0.85)

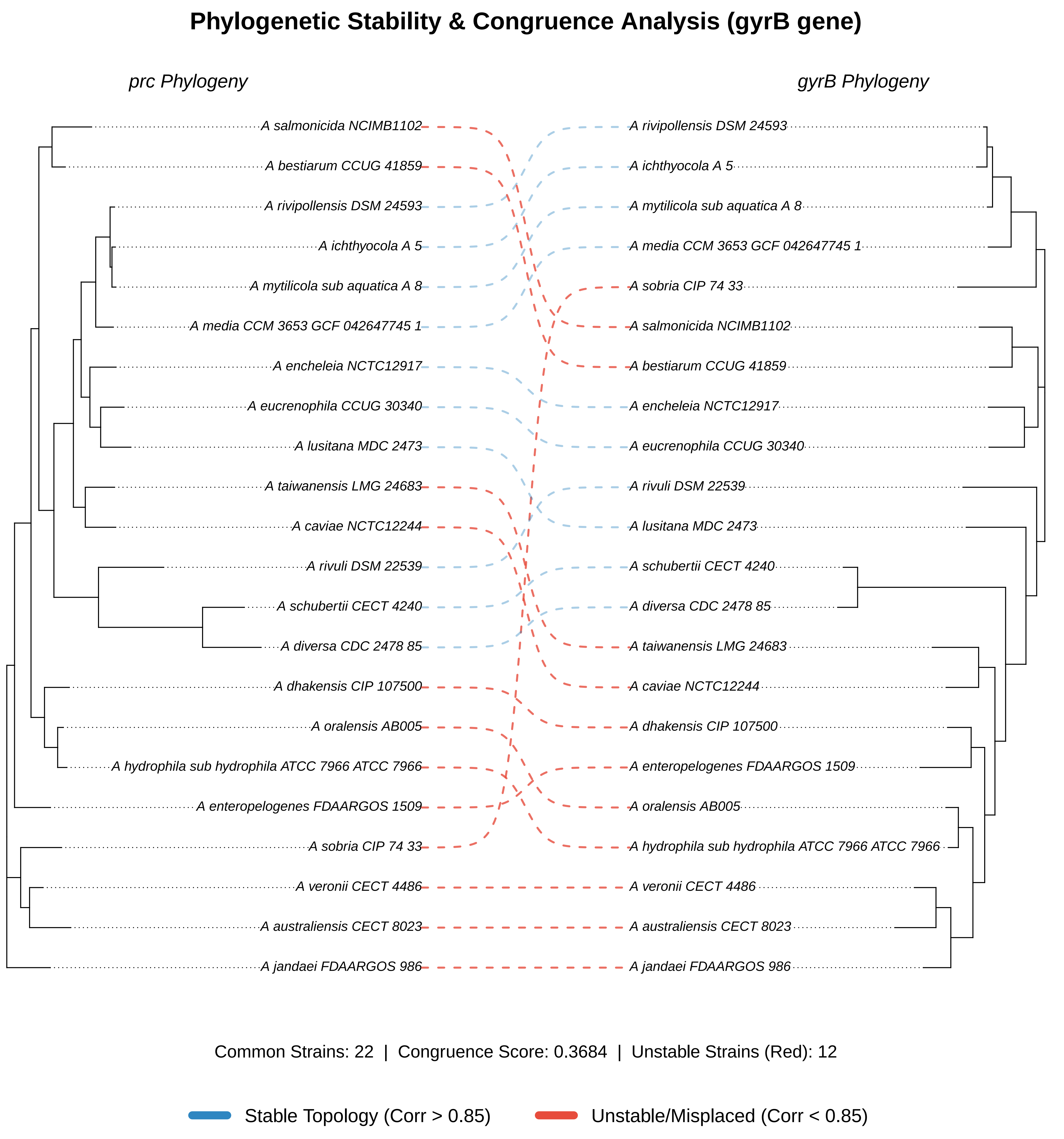
Phylogenetic Stability & Congruence Analysis (gyrB gene)
prc Phylogeny
gyrB Phylogeny
A salmonicida NCIMB1102
A bestiarum CCUG 41859
A rivipollensis DSM 24593
A ichthyocola A 5
A mytilicola sub aquatica A 8
A media CCM 3653 GCF 042647745 1
A encheleia NCTC12917
A eucrenophila CCUG 30340
A lusitana MDC 2473
A taiwanensis LMG 24683
A caviae NCTC12244
A rivuli DSM 22539
A schubertii CECT 4240
A diversa CDC 2478 85
A dhakensis CIP 107500
A oralensis AB005
A hydrophila sub hydrophila ATCC 7966 ATCC 7966
A enteropelogenes FDAARGOS 1509
A sobria CIP 74 33
A veronii CECT 4486
A australiensis CECT 8023
A jandaei FDAARGOS 986
A rivipollensis DSM 24593
A ichthyocola A 5
A mytilicola sub aquatica A 8
A media CCM 3653 GCF 042647745 1
A sobria CIP 74 33
A salmonicida NCIMB1102
A bestiarum CCUG 41859
A encheleia NCTC12917
A eucrenophila CCUG 30340
A rivuli DSM 22539
A lusitana MDC 2473
A schubertii CECT 4240
A diversa CDC 2478 85
A taiwanensis LMG 24683
A caviae NCTC12244
A dhakensis CIP 107500
A enteropelogenes FDAARGOS 1509
A oralensis AB005
A hydrophila sub hydrophila ATCC 7966 ATCC 7966
A veronii CECT 4486
A australiensis CECT 8023
A jandaei FDAARGOS 986
Common Strains: 22 | Congruence Score: 0.3684 | Unstable Strains (Red): 12
Stable Topology (Corr > 0.85)
Unstable/Misplaced (Corr < 0.85)

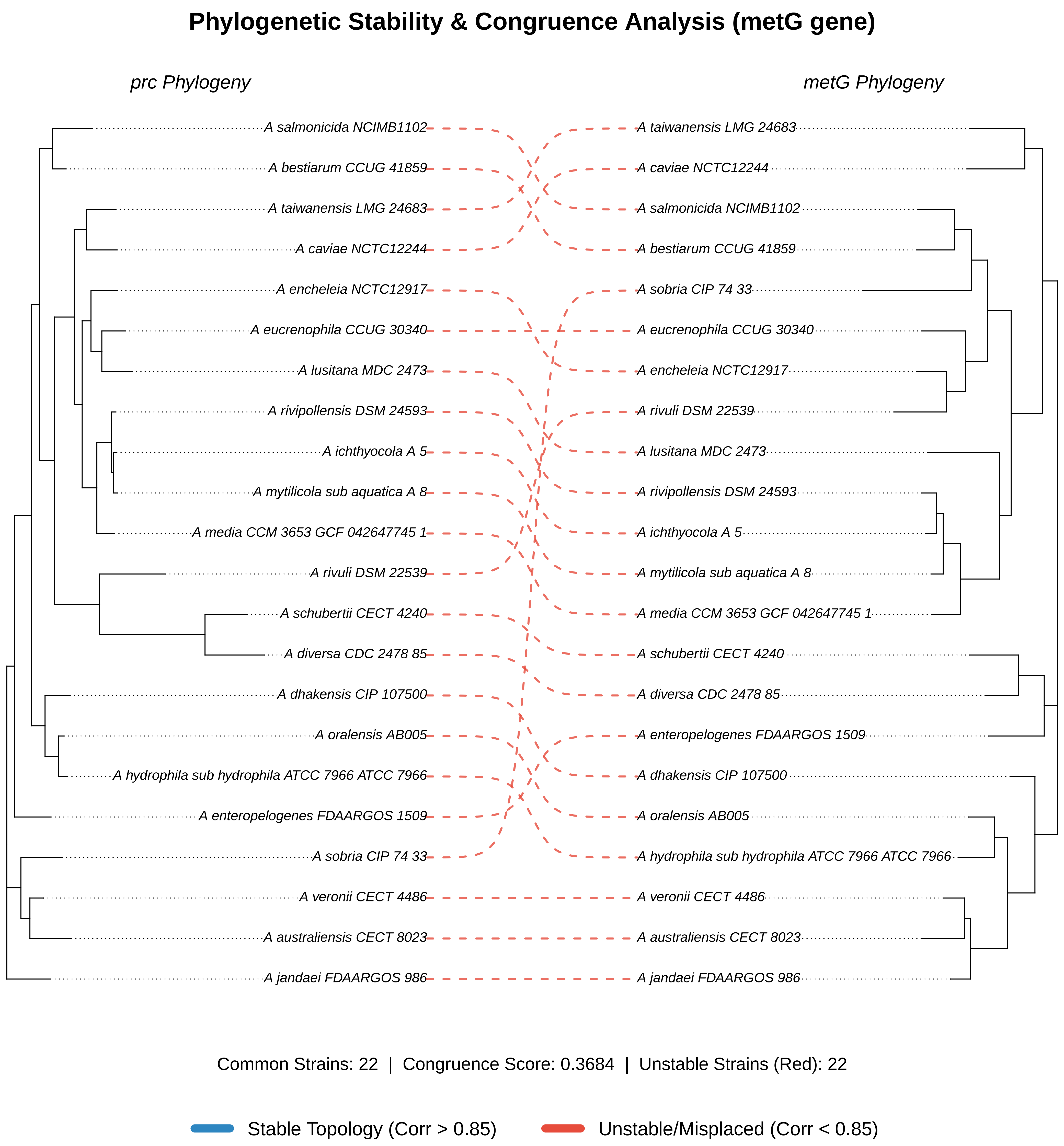
Phylogenetic Stability & Congruence Analysis (metG gene)
prc Phylogeny
metG Phylogeny
A salmonicida NCIMB1102
A bestiarum CCUG 41859
A taiwanensis LMG 24683
A caviae NCTC12244
A encheleia NCTC12917
A eucrenophila CCUG 30340
A lusitana MDC 2473
A rivipollensis DSM 24593
A ichthyocola A 5
A mytilicola sub aquatica A 8
A media CCM 3653 GCF 042647745 1
A rivuli DSM 22539
A schubertii CECT 4240
A diversa CDC 2478 85
A dhakensis CIP 107500
A oralensis AB005
A hydrophila sub hydrophila ATCC 7966 ATCC 7966
A enteropelogenes FDAARGOS 1509
A sobria CIP 74 33
A veronii CECT 4486
A australiensis CECT 8023
A jandaei FDAARGOS 986
Common Strains: 22 | Congruence Score: 0.3684 | Unstable Strains (Red): 22
Stable Topology (Corr > 0.85)
Unstable/Misplaced (Corr < 0.85)

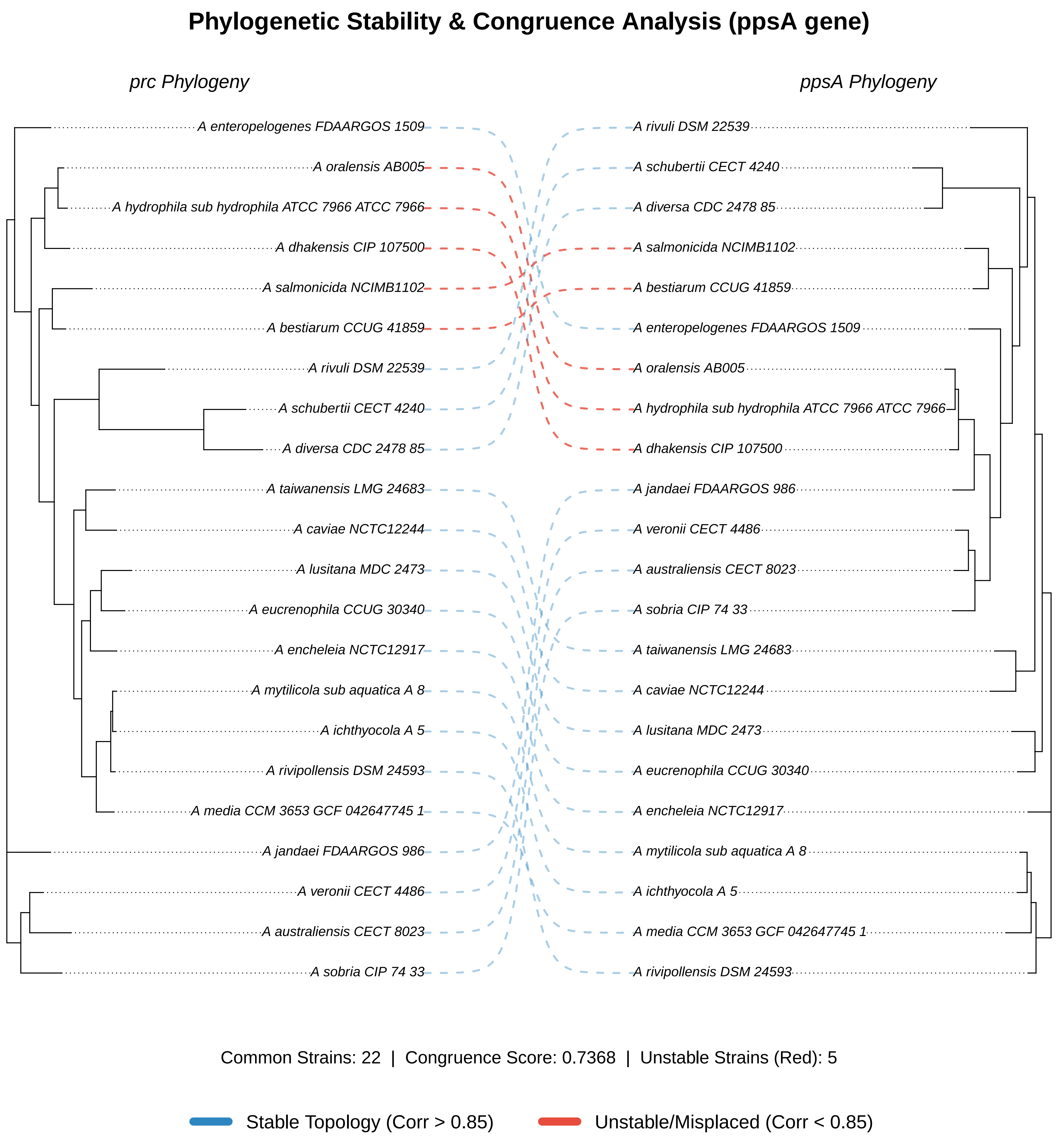

Phylogenetic Stability & Congruence Analysis (ppsA gene)
prc Phylogeny
ppsA Phylogeny
A enteropelogenes FDAARGOS 1509
A oralensis AB005
A hydrophila sub hydrophila ATCC 7966 ATCC 7966
A dhakensis CIP 107500
A salmonicida NCIMB1102
A bestiarum CCUG 41859
A rivuli DSM 22539
A schubertii CECT 4240
A diversa CDC 2478 85
A taiwanensis LMG 24683
A caviae NCTC12244
A lusitana MDC 2473
A eucrenophila CCUG 30340
A encheleia NCTC12917
A mytilicola sub aquatica A 8
A ichthyocola A 5
A rivipollensis DSM 24593
A media CCM 3653 GCF 042647745 1
A jandaei FDAARGOS 986
A veronii CECT 4486
A australiensis CECT 8023
A sobria CIP 74 33
A rivuli DSM 22539
A schubertii CECT 4240
A diversa CDC 2478 85
A salmonicida NCIMB1102
A bestiarum CCUG 41859
A enteropelogenes FDAARGOS 1509
A oralensis AB005
A hydrophila sub hydrophila ATCC 7966 ATCC 7966
A dhakensis CIP 107500
A jandaei FDAARGOS 986
A veronii CECT 4486
A australiensis CECT 8023
A sobria CIP 74 33
A taiwanensis LMG 24683
A caviae NCTC12244
A lusitana MDC 2473
A eucrenophila CCUG 30340
A encheleia NCTC12917
A mytilicola sub aquatica A 8
A ichthyocola A 5
A media CCM 3653 GCF 042647745 1
A rivipollensis DSM 24593
Common Strains: 22 | Congruence Score: 0.7368 | Unstable Strains (Red): 5
Stable Topology (Corr > 0.85)
Unstable/Misplaced (Corr < 0.85)

Phylogenetic Stability & Congruence Analysis (recA gene)

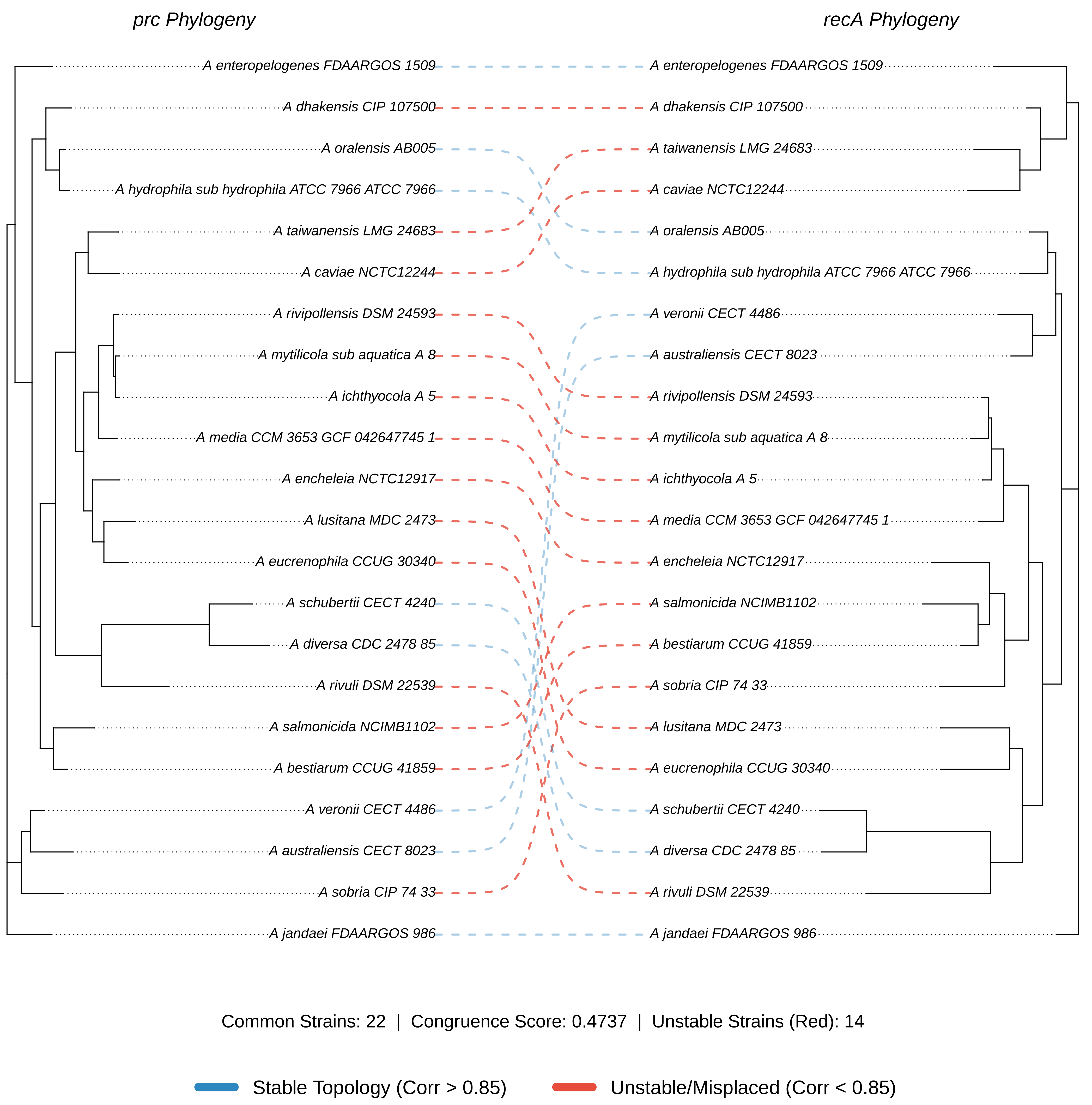

Table S1: The complete metadate of Aeromonas spp. types materials

Organism Scientific Name	Organism Qualifier	Taxonomy ic	Assembly Name	Assembly Accession	Source	Annotation	Level	Contig N50	Size	Submission D	Gene Coun	BioProject	BioSample
Aeromonas hydrophila subsp. hydrophila ATCC 7966	strain: ATCC 7966	380703	ASM1480v1	GCF_000014805.1	RefSeq	GCF_000014805.1-RS_2025_12_11	Complete (	4744448	4744448	11/6/2006	4357	PRJNA1669	SAMN02604052
Aeromonas diversa CDC 2478-85	strain: 2478-85	1268237	Adiv1.0	GCF_000367845.1	RefSeq	GCF_000367845.1-RS_2026_01_23	Contig	64780	4026421	4/19/2013	3870	PRJNA1836	SAMN02471398
Aeromonas molluscorum 848	strain: 848	1268236	Amol1.0	GCF_000388115.1	RefSeq	GCF_000388115.1-RS_2025_12_11	Contig	21565	4236171	5/3/2013	4146	PRJNA1836	SAMN02471397
Aeromonas salmonicida subsp. pectinolytica 34mel	strain: 34mel	1324960	ASM44743v2	GCF_000447435.2	RefSeq	GCF_000447435.2-RS_2025_03_27	Contig	47147	4767351	4/23/2015	4502	PRJNA1984	SAMN03322533
Aeromonas salmonicida subsp. masoucida NBRC 13784	strain: NBRC 13784	1216980	ASM64795v1	GCF_000647955.1	RefSeq	GCF_000647955.1-RS_2024_09_03	Contig	43858	4502258	4/15/2014	4277	PRJDB242	SAMD00000014
Aeromonas taiwanensis	strain: LMG 24683	633417	ASM69918v1	GCF_000699185.1	RefSeq	GCF_000699185.1-RS_2025_08_09	Contig	75335	4230588	5/22/2014	3974	PRJDB1639	SAMD00000007
Aeromonas finlandensis	strain: 4287D	1543375	ASM76464v1	GCF_000764645.1	RefSeq	GCF_000764645.1-RS_2025_04_02	Contig	31035	4717507	10/8/2014	4549	PRJNA2604	SAMN03023686
Aeromonas aquatica	strain: AE235	558964	ASM76465v1	GCF_000764655.1	RefSeq	GCF_000764655.1-RS_2025_03_27	Contig	67531	4582304	10/8/2014	4292	PRJNA2604	SAMN03023875
Aeromonas lacus	strain: AE122	558884	ASM76466v1	GCF_000764665.1	RefSeq	GCF_000764665.1-RS_2025_03_27	Contig	79727	4394373	10/8/2014	4144	PRJNA2604	SAMN03023876
Aeromonas allosaccharophila	strain: CECT 4199	656	PRJEB7019	GCF_000819685.1	RefSeq	GCF_000819685.1-RS_2025_04_21	Scaffold	114541	4663841	11/4/2014	4364	PRJEB7019	SAMEA2752428
Aeromonas dhakensis	strain: CECT 7289	196024	PRJEB7020	GCF_000819705.1	RefSeq	GCF_000819705.1-RS_2026_02_10	Scaffold	111283	4692336	12/1/2014	4432	PRJEB7020	SAMEA2752427
Aeromonas australiensis	strain: CECT 8023	1114880	PRJEB7021	GCF_000819725.1	RefSeq	GCF_000819725.1-RS_2025_04_24	Contig	95095	4110035	12/1/2014	3813	PRJEB7021	SAMEA2752426
Aeromonas bestiarum	strain: CECT 4227	105751	PRJEB7022	GCF_000819745.1	RefSeq	GCF_000819745.1-RS_2025_04_24	Scaffold	135742	4690820	12/1/2014	4287	PRJEB7022	SAMEA2752425
Aeromonas bivalvium	strain: CECT 7113	440079	PRJEB7023	GCF_000819765.1	RefSeq	GCF_000819765.1-RS_2025_12_22	Scaffold	70248	4302589	12/1/2014	4027	PRJEB7023	SAMEA2752424
Aeromonas caviae	strain: CECT 838	648	PRJEB7024	GCF_000819785.1	RefSeq	GCF_000819785.1-RS_2026_02_02	Scaffold	71441	4471402	12/1/2014	4202	PRJEB7024	SAMEA2752423
Aeromonas diversa CDC 2478-85	strain: CECT 4254	1268237	PRJEB7026	GCF_000819805.1	RefSeq	GCF_000819805.1-RS_2025_08_25	Scaffold	140915	4062507	12/1/2014	3865	PRJEB7026	SAMEA2752422
Aeromonas encheleia	strain: CECT 4342	73010	PRJEB7027	GCF_000819825.1	RefSeq	GCF_000819825.1-RS_2026_02_15	Scaffold	170289	4472527	12/1/2014	4181	PRJEB7027	SAMEA2752421
Aeromonas enteropelogenes	strain: CECT 4487	29489	PRJEB7028	GCF_000819845.1	RefSeq	GCF_000819845.1-RS_2026_01_24	Scaffold	119583	4474738	12/1/2014	4205	PRJEB7028	SAMEA2752420
Aeromonas eucrenophila	strain: CECT 4224	649	PRJEB7029	GCF_000819865.1	RefSeq	GCF_000819865.1-RS_2025_12_11	Scaffold	221169	4540059	12/1/2014	4189	PRJEB7029	SAMEA2752419
Aeromonas fluvialis	strain: LMG 24681	591962	PRJEB7030	GCF_000819885.1	RefSeq	GCF_000819885.1-RS_2025_04_24	Scaffold	96092	3904228	12/1/2014	3725	PRJEB7030	SAMEA2752418
Aeromonas jandaei	strain: CECT 4228	650	PRJEB7031	GCF_000819955.1	RefSeq	GCF_000819955.1-RS_2025_04_24	Scaffold	90736	4500350	12/1/2014	4274	PRJEB7031	SAMEA2752417
Aeromonas media	strain: CECT 4232	651	PRJEB7032	GCF_000819985.1	RefSeq	GCF_000819985.1-RS_2026_02_10	Scaffold	33307	4484338	12/1/2014	4307	PRJEB7032	SAMEA2752416
Aeromonas piscicola	strain: LMG 24783	600645	PRJEB7033	GCF_000820005.1	RefSeq	GCF_000820005.1-RS_2025_03_30	Scaffold	100594	5177970	12/1/2014	4972	PRJEB7033	SAMEA2752415
Aeromonas popoffii	strain: CIP 105493	70856	PRJEB7034	GCF_000820025.1	RefSeq	GCF_000820025.1-RS_2025_04_24	Scaffold	74094	4762473	12/1/2014	4524	PRJEB7034	SAMEA2752414
Aeromonas rivuli	strain: DSM 22539	648794	PRJEB7035	GCF_000820045.1	RefSeq	GCF_000820045.1-RS_2025_08_20	Scaffold	120760	4534147	12/1/2014	4332	PRJEB7035	SAMEA2752413
Aeromonas salmonicida subsp. salmonicida	strain: CIP 103209	29491	PRJEB7036	GCF_000820065.1	RefSeq	GCF_000820065.1-RS_2025_12_20	Scaffold	89543	4740147	12/1/2014	4573	PRJEB7036	SAMEA2752412
Aeromonas sanarellii	strain: LMG 24682	633415	PRJEB7037	GCF_000820085.1	RefSeq	GCF_000820085.1-RS_2026_01_19	Scaffold	82330	4186903	12/1/2014	3947	PRJEB7037	SAMEA2752411
Aeromonas schubertii	strain: CECT 4240	652	PRJEB7038	GCF_000820105.1	RefSeq	GCF_000820105.1-RS_2026_02_10	Scaffold	108810	4126255	1/30/2015	3998	PRJEB7038	SAMEA2752410
Aeromonas simiae	strain: CIP 107798	218936	PRJEB7039	GCF_000820125.1	RefSeq	GCF_000820125.1-RS_2026_02_15	Scaffold	64204	3987694	12/1/2014	3856	PRJEB7039	SAMEA2752409
Aeromonas sobria	strain: CECT 4245	646	PRJEB7040	GCF_000820145.1	RefSeq	GCF_000820145.1-RS_2025_11_22	Scaffold	171779	4683669	12/1/2014	4345	PRJEB7040	SAMEA2752408
Aeromonas taiwanensis	strain: LMG 24683	633417	PRJEB7041	GCF_000820165.1	RefSeq	GCF_000820165.1-RS_2025_12_11	Scaffold	85210	4250891	12/1/2014	3988	PRJEB7041	SAMEA2752407
Aeromonas tecta	strain: CECT 7082	324617	PRJEB7042	GCF_000820185.1	RefSeq	GCF_000820185.1-RS_2025_12_13	Scaffold	201578	4755221	1/30/2015	4444	PRJEB7042	SAMEA2752406
Aeromonas enteropelogenes	strain: CECT 4255T	29489	PRJEB7043	GCF_000820205.1	RefSeq	GCF_000820205.1-RS_2026_01_15	Scaffold	224321	4337245	12/1/2014	4007	PRJEB7043	SAMEA2752405
Aeromonas veronii bv. veronii	strain: CECT 4257	197701	PRJEB7044	GCF_000820225.1	RefSeq	GCF_000820225.1-RS_2025_03_21	Scaffold	148348	4516420	12/1/2014	4212	PRJEB7044	SAMEA2752404
Aeromonas caviae	strain: CECT 4221	648	PRJEB7046	GCF_000820265.1	RefSeq	GCF_000820265.1-RS_2025_08_20	Scaffold	30787	4579196	1/30/2015	4447	PRJEB7046	SAMEA2752402
Aeromonas veronii	strain: CIP 107763	654	PRJEB7047	GCF_000820285.1	RefSeq	GCF_000820285.1-RS_2025_12_22	Contig	188049	4430813	12/1/2014	4137	PRJEB7047	SAMEA2752401
Aeromonas dhakensis	strain: CIP 107500	196024	PRJEB7048	GCF_000820305.1	RefSeq	GCF_000820305.1-RS_2026_02_10	Scaffold	165885	4711881	12/1/2014	4406	PRJEB7048	SAMEA2752400
Aeromonas hydrophila subsp. ranae	strain: CIP 107985	208958	PRJEB7049	GCF_000820325.1	RefSeq	GCF_000820325.1-RS_2025_08_20	Scaffold	88476	4682559	12/1/2014	4445	PRJEB7049	SAMEA2752399
Aeromonas veronii	strain: CECT 4486	654	PRJEB7050	GCF_000820365.1	RefSeq	GCF_000820365.1-RS_2025_12_22	Scaffold	90706	4410797	12/1/2014	4115	PRJEB7050	SAMEA2752398
Aeromonas schubertii	strain: ATCC 43700	652	ASM148139v1	GCF_001481395.1	RefSeq	GCF_001481395.1-RS_2025_12_12	Scaffold	144100	4140228	12/22/2015	3971	PRJNA3043	SAMN04299626
Aeromonas salmonicida subsp. salmonicida	strain: ATCC 33658	29491	ASM164330v1	GCF_001643305.1	RefSeq	GCF_001643305.1-RS_2025_12_20	Contig	89474	4728143	5/13/2016	4539	PRJNA3102	SAMN04449999
Aeromonas veronii bv. veronii	strain: CCM 4359	197701	ASM190853v1	GCF_001908535.1	RefSeq	GCF_001908535.1-RS_2025_08_16	Contig	245067	4511265	12/19/2016	4236	PRJNA3453	SAMN05860752
Aeromonas hydrophila	strain: ATCC 7966	644	ASM199968v1	GCF_001999685.1	RefSeq	GCF_001999685.1-RS_2025_05_17	Scaffold	132049	4673830	2/13/2017	4419	PRJNA3414	SAMN05721785
Aeromonas salmonicida subsp. pectinolytica 34mel	strain: 34mel	1324960	ASM273522v1	GCF_002735225.1	RefSeq	GCF_002735225.1-RS_2025_04_03	Complete (	5012649	5012649	10/27/2017	4645	PRJNA2992	SAMN04208118
Aeromonas cavernicola	strain: DSM 24474	1006623	ASM279530v1	GCF_002795305.1	RefSeq	GCF_002795305.1-RS_2025_03_28	Contig	36274	3887629	11/25/2017	3757	PRJNA4172	SAMN07981412
Aeromonas lusitana	strain: MDC 2473	931529	MDC2473	GCF_002812985.1	RefSeq	GCF_002812985.1-RS_2025_04_22	Scaffold	156055	4537799	11/16/2017	4171	PRJNA4172	SAMN07981238
Aeromonas jandaei	strain: FDAARGOS_986	650	ASM1612719v1	GCF_016127195.1	RefSeq	GCF_016127195.1-RS_2025_12_13	Complete (	4551079	4551079	12/20/2020	4261	PRJNA2312	SAMN16357155
Aeromonas veronii bv. veronii	strain: ATCC 35624	197701	ASM1835990v1	GCF_018359905.1	RefSeq	GCF_018359905.1-RS_2025_04_06	Contig	373301	4508886	5/14/2021	4189	PRJNA6859	SAMN17101839
Aeromonas sobria	strain: CIP 74.33	646	ASM1835994v1	GCF_018359945.1	RefSeq	GCF_018359945.1-RS_2025_04_26	Contig	238489	4689240	5/14/2021	4361	PRJNA6859	SAMN17101838
Aeromonas media	strain: ATCC 33907	651	ASM1835998v1	GCF_018359985.1	RefSeq	GCF_018359985.1-RS_2025_05_22	Contig	60371	4469144	5/14/2021	4253	PRJNA6859	SAMN17101837
Aeromonas caviae	strain: CECT 838	648	ASM1836000v1	GCF_018360005.1	RefSeq	GCF_018360005.1-RS_2025_03_13	Contig	287223	4475647	5/14/2021	4165	PRJNA6859	SAMN17101836
Aeromonas hydrophila	strain: ATCC 7966	644	ASM1836002v1	GCF_018360025.1	RefSeq	GCF_018360025.1-RS_2025_04_26	Contig	896486	4680708	5/14/2021	4323	PRJNA6859	SAMN17101835
Aeromonas enteropelogenes	strain: FDAARGOS_1510	29489	ASM2009729v1	GCF_020097295.1	RefSeq	GCF_020097295.1-RS_2025_12_12	Complete (	4392400	4392400	9/23/2021	4046	PRJNA2312	SAMN21218866
Aeromonas enteropelogenes	strain: FDAARGOS_1509	29489	ASM2009731v1	GCF_020097315.1	RefSeq	GCF_020097315.1-RS_2026_01_30	Complete (	4540592	4540592	9/23/2021	4240	PRJNA2312	SAMN21218865
Aeromonas jandaei	strain: FDAARGOS_1523	650	ASM2034153v1	GCF_020341535.1	RefSeq	GCF_020341535.1-RS_2025_12_02	Chromosor	4545460	4545560	10/7/2021	4259	PRJNA2312	SAMN21402690
Aeromonas caviae subsp. aquatica	strain: V15	3418572	ASM2567450v1	GCF_025674505.1	RefSeq	GCF_025674505.1-RS_2025_08_03	Contig	272761	4443347	10/12/2022	4158	PRJNA8878	SAMN31202178
Aeromonas hydrophila	strain: ATCC 7966	644	ASM3028606v1	GCF_030286065.1	RefSeq	GCF_030286065.1-RS_2025_06_19	Scaffold	120255	4739633	6/18/2023	4434	PRJNA9805	SAMN35638130
Aeromonas jandaei	strain: DSM 7311	650	ASM3789069v1	GCF_037890695.1	RefSeq	GCF_037890695.1-RS_2025_04_05	Complete (	4551172	4551172	4/5/2024	4266	PRJNA1087	SAMN40453449
Aeromonas salmonicida subsp. achromogenes	strain: JCM 7875	113288	ASM3952371v1	GCF_039523715.1	RefSeq	GCF_039523715.1-RS_2025_05_10	Scaffold	40020	4461734	2/23/2024	4336	PRJDB1051	SAMD00645021
Aeromonas media	strain: CCM 3653	651	ASM4243358v1	GCF_042433585.1	RefSeq	GCF_042433585.1-RS_2025_09_29	Contig	59086	4479849	9/28/2024	4255	PRJNA5957	SAMN43281582
Aeromonas media	strain: CCM 3653	651	ASM4264774v1	GCF_042647745.1	RefSeq	GCF_042647745.1-RS_2025_12_15	Contig	4717863	4881877	10/5/2024	4536	PRJNA5957	SAMN43282171
Aeromonas bestiarum	strain: CCUG 41859	105751	ASM4265672v1	GCF_042656725.1	RefSeq	GCF_042656725.1-RS_2025_03_02	Scaffold	234012	4692613	10/6/2024	4295	PRJNA5957	SAMN43282751
Aeromonas eucrenophila	strain: CCUG 30340	649	ASM4265676v1	GCF_042656765.1	RefSeq	GCF_042656765.1-RS_2025_03_02	Scaffold	440942	4543675	10/6/2024	4206	PRJNA5957	SAMN43282750
Aeromonas mytilicola subsp. aquatica	strain: A-8	3366579	ASM4433638v1	GCF_044336385.1	RefSeq	GCF_044336385.1-RS_2024_11_05	Complete (	4681979	4681979	11/6/2024	4297	PRJNA1168	SAMN44019497
Aeromonas media	strain: DSM 4881	651	ASM4501480v1	GCF_045014805.1	RefSeq	GCF_045014805.1-RS_2024_11_19	Complete (	4699671	4706660	11/20/2024	4432	PRJNA6157	SAMN14466697
Aeromonas ichthyocola	strain: A-5	3367746	ASM4608798v1	GCF_046087985.1	RefSeq	GCF_046087985.1-RS_2025_12_15	Contig	762425	4676890	12/14/2024	4525	PRJNA1170	SAMN44091370
Aeromonas rivipollensis	strain: DSM 24593	948519	57467	GCF_046226845.1	RefSeq	GCF_046226845.1-RS_2024_12_20	Complete (	4949764	5254087	1/26/2025	4935	PRJNA1068	SAMN39589610
Aeromonas oralensis	strain: AB005	3415010	ASM4966557v1	GCF_049665575.1	RefSeq	GCF_049665575.2-RS_2025_06_11	Complete (	4784854	4784854	8/4/2025	4484	PRJNA1247	SAMN47828375
Aeromonas salmonicida	strain: NCTC12959	645	52121_E02	GCF_900445115.1	RefSeq	GCF_900445115.1-RS_2025_04_04	Contig	2849290	4934557	6/29/2018	4751	PRJEB6403	SAMEA4530645
Aeromonas hydrophila	strain: NCTC8049	644	49243_E02	GCF_900445175.1	RefSeq	GCF_900445175.1-RS_2025_08_16	Contig	4744529	4752356	8/8/2018	4368	PRJEB6403	SAMEA4076704
Aeromonas caviae	strain: NCTC12244	648	51438_H01	GCF_900476005.1	RefSeq	GCF_900476005.1-RS_2025_12_14	Complete (	4586140	4586140	6/17/2018	4258	PRJEB6403	SAMEA4475690
Aeromonas encheleia	strain: NCTC12917	73010	51438_G01	GCF_900637545.1	RefSeq	GCF_900637545.1-RS_2025_12_13	Complete (	4542521	4542521	12/20/2018	4236	PRJEB6403	SAMEA4475689
Aeromonas salmonicida	isolate: NCIMB1102	645	NCIMB1102	GCF_963425675.1	RefSeq	GCF_963425675.1-RS_2024_10_03	Chromosor	115076	4512221	10/10/2024	4247	PRJEB6584	SAMEA114323047
Aeromonas bestiarum	isolate: CIP74.30T, strain: CIP74.30T	105751	CIP74.30T	GCF_965138935.1	RefSeq	GCF_965138935.1-RS_2026_02_14	Scaffold	234276	4697648	2/13/2025	4355	PRJEB8543	SAMEA117660721

Table S2: The complete metadate of Aeromonas spp. complete genomes

Organism Scientific Name	Organism Qualifier	Taxonomy id	Assembly Name	Assembly Accession	Source	Annotation	Level	Contig N50	Size	Submission Dat	Gene Coun	BioProject	BioSample
Aeromonas hydrophila subsp. hydrophila ATCC 7966	strain: ATCC 7966	380703	ASM1480v1	GCF_000014805.1	RefSeq	GCF_000014805.1-RS_2025_12_11	Complete G	4744448	4744448	11/6/2006	4357	PRJNA1669	SAMN02604052
Aeromonas salmonicida subsp. salmonicida A449	strain: A449	382245	ASM19639v1	GCF_000196395.1	RefSeq	GCF_000196395.1-RS_2025_04_08	Complete G	4702402	5040536	4/6/2007	4820	PRJNA1672	SAMN02603664
Aeromonas veronii B565	strain: B565	998088	ASM20411v1	GCF_000204115.1	RefSeq	GCF_000204115.1-RS_2025_03_20	Complete G	4551783	4551783	4/13/2011	4204	PRJNA6367	SAMN02603940
Aeromonas media WS	strain: WS	1208104	ASM28721v3	GCF_000287215.2	RefSeq	GCF_000287215.2-RS_2026_01_15	Complete G	4777154	4788430	4/22/2014	4476	PRJNA1701	SAMN02472129
Aeromonas hydrophila ML09-119	strain: ML09-119	1288394	ASM40155v1	GCF_000401555.1	RefSeq	GCF_000401555.1-RS_2025_12_15	Complete G	5024500	5024500	5/24/2013	4661	PRJNA1881	SAMN02603614
Aeromonas veronii Hm21	strain: Hm21	1347420	ASM46451v2	GCF_000464515.2	RefSeq	GCF_000464515.2-RS_2025_03_15	Complete G	4710355	4766880	7/31/2020	4427	PRJNA2058	SAMN02471703
Aeromonas hydrophila 4AK4	strain: 4AK4	1354302	ASM51218v1	GCF_000512185.1	RefSeq	NCBI Prokaryotic Genome Annotation Pipeline (PGAP)	Complete G	4527993	4527993	12/30/2013	4180	PRJNA2105	SAMN02641590
Aeromonas hydrophila YL17	strain: YL17	1448139	ASM61207v2	GCF_000612075.2	RefSeq	NCBI Prokaryotic Genome Annotation Pipeline (PGAP)	Complete G	4796281	4796281	1/11/2016	4397	PRJNA2344	SAMN02713488
Aeromonas hydrophila subsp. hydrophila AL09-71	isolate: 4, strain: AL09-71	1321367	ASM63317v1	GCF_000633175.1	RefSeq	GCF_000633175.1-RS_2025_04_23	Complete G	5023861	5023861	4/18/2014	4669	PRJNA2270	SAMN02726090
Aeromonas hydrophila pc104A	strain: PC-104A	1418107	ASM63595v1	GCF_000635955.1	RefSeq	GCF_000635955.1-RS_2025_12_16	Complete G	5023829	5023829	4/22/2014	4668	PRJNA2270	SAMN02728499
Aeromonas caviae	strain: FDAARGOS_72	648	ASM78377v2	GCF_000783775.2	RefSeq	GCF_000783775.2-RS_2026_01_29	Complete G	4518310	4518310	1/29/2018	4175	PRJNA2312	SAMN02934523
Aeromonas hydrophila J-1	strain: J-1	1419584	ASM81950v1	GCF_000819505.1	RefSeq	GCF_000819505.1-RS_2026_01_31	Complete G	5000814	5000814	1/16/2015	4629	PRJNA2272	SAMN03283200
Aeromonas hydrophila	isolate: Goldfish, strain: AL06-06	644	ASM94091v1	GCF_000940915.1	RefSeq	GCF_000940915.1-RS_2025_12_19	Complete G	4884823	4901389	3/4/2015	4593	PRJNA2708	SAMN03366652
Aeromonas caviae	strain: 8LM	648	ASM95970v2	GCF_000959705.2	RefSeq	NCBI Prokaryotic Genome Annotation Pipeline (PGAP)	Complete G	4551368	4551368	11/2/2017	4261	PRJNA2773	SAMN03389335
Aeromonas hydrophila	strain: AH10	644	ASM96364v1	GCF_000963645.1	RefSeq	GCF_000963645.1-RS_2026_01_31	Complete G	4908265	4908265	3/26/2015	4547	PRJNA2785	SAMN03421355
Aeromonas hydrophila NJ-35	strain: NJ-35	1416915	ASM101964v1	GCF_001019645.1	RefSeq	GCF_001019645.1-RS_2025_04_13	Complete G	5279644	5279644	6/3/2015	4919	PRJNA2262	SAMN03744001
Aeromonas schubertii	strain: WL1483	652	ASM144733v1	GCF_001447335.1	RefSeq	GCF_001447335.1-RS_2026_03_03	Complete G	4400034	4400034	11/23/2015	4323	PRJNA2971	SAMN04110141
Aeromonas hydrophila	strain: JBN2301	644	ASM145536v1	GCF_001455365.1	RefSeq	GCF_001455365.1-RS_2026_03_03	Complete G	5127362	5145887	12/2/2015	4813	PRJNA3021	SAMN04266592
Aeromonas hydrophila	strain: D4	644	ASM151877v1	GCF_001518775.1	RefSeq	GCF_001518775.1-RS_2026_03_03	Complete G	5100520	5275132	1/20/2016	4927	PRJNA3086	SAMN04404355
Aeromonas veronii	strain: TH0426	654	ASM159324v1	GCF_001593245.1	RefSeq	GCF_001593245.1-RS_2025_12_29	Complete G	4923009	4923009	3/16/2016	4639	PRJNA2939	SAMN04012505
Aeromonas veronii	strain: AVNIH1	654	ASM163432v1	GCF_001634325.1	RefSeq	GCF_001634325.1-RS_2024_03_18	Complete G	4756751	4955058	4/29/2016	4619	PRJNA2796	SAMN03455926
Aeromonas veronii	strain: CB51	654	ASM163434v1	GCF_001634345.1	RefSeq	NCBI Prokaryotic Genome Annotation Pipeline (PGAP)	Complete G	4584103	4584103	4/29/2016	4248	PRJNA3196	SAMN04901561
Aeromonas salmonicida subsp. salmonicida	strain: J223	29491	ASM164327v2	GCF_001643275.2	RefSeq	GCF_001643275.2-RS_2025_03_27	Complete G	4702618	4897684	2/5/2020	4678	PRJNA3102	SAMN04449844
Aeromonas salmonicida subsp. salmonicida	strain: J227	29491	ASM164335v2	GCF_001643355.2	RefSeq	GCF_001643355.2-RS_2025_04_08	Complete G	4698084	4894354	12/15/2022	4670	PRJNA3102	SAMN04450000
Aeromonas hydrophila	strain: GYK1	644	ASM168353v1	GCF_001683535.1	RefSeq	GCF_001683535.1-RS_2026_03_05	Complete G	4951765	4951765	7/11/2016	4547	PRJNA3237	SAMN05188441
Aeromonas hydrophila	strain: AHNIH1	644	ASM168712v1	GCF_001687125.1	RefSeq	GCF_001687125.1-RS_2026_03_05	Complete G	4906118	5049466	7/18/2016	4732	PRJNA2736	SAMN03294822
Aeromonas hydrophila	strain: MX16A	644	ASM189596v1	GCF_001895965.1	RefSeq	GCF_001895965.1-RS_2025_04_30	Complete G	4783504	4783504	12/9/2016	4416	PRJNA3535	SAMN06016471
Aeromonas salmonicida	strain: O23A	645	ASM218033v1	GCF_002180335.1	RefSeq	GCF_002180335.1-RS_2026_02_16	Complete G	4811606	4935342	6/8/2017	4603	PRJNA3838	SAMN06812489
Aeromonas salmonicida	strain: S121	645	ASM221424v1	GCF_002214245.1	RefSeq	GCF_002214245.1-RS_2026_02_16	Complete G	4723196	5069859	7/3/2017	4861	PRJNA3918	SAMN07276469
Aeromonas salmonicida	strain: S68	645	ASM221426v1	GCF_002214265.1	RefSeq	GCF_002214265.1-RS_2026_02_16	Complete G	4730032	4880825	7/3/2017	4664	PRJNA3918	SAMN07276873
Aeromonas salmonicida	strain: S44	645	ASM221430v1	GCF_002214305.1	RefSeq	GCF_002214305.1-RS_2026_02_16	Complete G	4715196	5082912	7/3/2017	4860	PRJNA3918	SAMN07276874
Aeromonas dhakensis	strain: KN-Mc-6U21	196024	ASM228593v1	GCF_002285935.1	RefSeq	GCF_002285935.1-RS_2025_07_23	Complete G	4868053	4868053	9/5/2017	4504	PRJNA4008	SAMN07577462
Aeromonas salmonicida subsp. masoucida	strain: RFAS1	197700	ASM231306v1	GCF_002313065.1	RefSeq	GCF_002313065.1-RS_2026_02_16	Complete G	4783004	4917535	9/19/2017	4723	PRJNA3420	SAMN05735119
Aeromonas sp. CU5	strain: CU5	2033033	ASM258674v1	GCF_002586745.1	RefSeq	GCF_002586745.1-RS_2024_07_22	Complete G	4615599	4615599	10/18/2017	4207	PRJNA4009	SAMN07585966
Aeromonas sp. CA23	strain: CA23	2033032	ASM258706v1	GCF_002587065.1	RefSeq	GCF_002587065.1-RS_2025_08_18	Complete G	5099209	5099209	10/18/2017	4739	PRJNA4009	SAMN07585949
Aeromonas salmonicida subsp. pectinolytica 34mel	strain: 34mel	1324960	ASM273522v1	GCF_002735225.1	RefSeq	GCF_002735225.1-RS_2025_04_03	Complete G	5012649	5012649	10/27/2017	4645	PRJNA2992	SAMN04208118
Aeromonas salmonicida	strain: A527	645	ASM276413v1	GCF_002764135.1	RefSeq	GCF_002764135.1-RS_2025_03_21	Complete G	4806250	4806250	11/13/2017	4478	PRJNA2643	SAMN07410266
Aeromonas veronii	strain: Aeromonas veronii X11	654	ASM280392v1	GCF_002803925.1	RefSeq	NCBI Prokaryotic Genome Annotation Pipeline (PGAP)	Complete G	4283286	4283286	12/4/2017	3897	PRJNA4190	SAMN08042683
Aeromonas veronii	strain: X12	654	ASM280394v1	GCF_002803945.1	RefSeq	GCF_002803945.1-RS_2025_12_21	Complete G	4773186	4773186	12/4/2017	4433	PRJNA4190	SAMN08042684
Aeromonas hydrophila subsp. hydrophila	strain: WCHAH045096	196023	ASM285069v3	GCF_002850695.3	RefSeq	GCF_002850695.3-RS_2025_12_27	Complete G	5022876	5365058	8/16/2019	5020	PRJNA4153	SAMN07821593
Aeromonas veronii	strain: FC951	654	ASM286688v2	GCF_002866885.2	RefSeq	GCF_002866885.2-RS_2025_04_16	Complete G	4666657	4863185	10/16/2018	4656	PRJNA4281	SAMN08281233
Aeromonas sp. ASNIH5	strain: ASNIH5	1758179	ASM290286v1	GCF_002902865.1	RefSeq	GCF_002902865.1-RS_2025_08_23	Complete G	4759223	4783196	1/29/2018	4494	PRJNA4308	SAMN04286971
Aeromonas sp. ASNIH3	strain: ASNIH3	1636608	ASM290340v1	GCF_002903405.1	RefSeq	GCF_002903405.1-RS_2025_08_24	Complete G	4797236	5033911	1/29/2018	4796	PRJNA4308	SAMN03455983
Aeromonas sp. ASNIH7	strain: ASNIH7	1920107	ASM290348v1	GCF_002903485.1	RefSeq	GCF_002903485.1-RS_2025_08_24	Complete G	4817060	4868657	1/29/2018	4542	PRJNA4308	SAMN06040385
Aeromonas sp. ASNIH1	strain: ASNIH1	1636606	ASM293428v1	GCF_002934285.1	RefSeq	GCF_002934285.1-RS_2025_08_24	Complete G	4984360	5079402	2/6/2018	4885	PRJNA4308	SAMN03455980
Aeromonas sp. ASNIH4	strain: ASNIH4	1636609	ASM293442v1	GCF_002934425.1	RefSeq	GCF_002934425.1-RS_2025_08_24	Complete G	5216518	5480196	2/6/2018	5181	PRJNA4308	SAMN03455984
Aeromonas sp. ASNIH2	strain: ASNIH2	1636607	ASM293479v1	GCF_002934795.1	RefSeq	GCF_002934795.1-RS_2025_08_24	Complete G	4801408	4864532	2/4/2018	4645	PRJNA4308	SAMN03455981
Aeromonas hydrophila	strain: KN-Mc-1R2	644	ASM301477v1	GCF_003014775.1	RefSeq	GCF_003014775.1-RS_2025_12_14	Complete G	4911246	4911246	3/24/2018	4608	PRJNA4384	SAMN08715271
Aeromonas rivipollensis	strain: KN-Mc-11N1	948519	ASM301516v1	GCF_003015165.1	RefSeq	GCF_003015165.1-RS_2025_04_02	Complete G	4508901	4508901	3/26/2018	4186	PRJNA4385	SAMN08721782
Aeromonas caviae	strain: R25-6	648	ASM329485v2	GCF_003294855.2	RefSeq	GCF_003294855.2-RS_2026_01_30	Complete G	4702913	4713384	7/16/2020	4440	PRJNA4284	SAMN08290453
Aeromonas caviae	strain: T25-39	648	ASM329489v2	GCF_003294895.2	RefSeq	NCBI Prokaryotic Genome Annotation Pipeline (PGAP)	Complete G	4650143	4974737	7/16/2020	4721	PRJNA4284	SAMN08290452
Aeromonas hydrophila	strain: ZYAH72	644	ASM349122v1	GCF_003491225.1	RefSeq	GCF_003491225.1-RS_2025_07_08	Complete G	5159182	5159182	9/7/2018	4789	PRJNA3393	SAMN05590109
Aeromonas hydrophila	strain: ZYAH75	644	ASM349124v1	GCF_003491245.1	RefSeq	GCF_003491245.1-RS_2025_04_03	Complete G	4955171	4955171	9/7/2018	4573	PRJNA3393	SAMN05589911
Aeromonas veronii	strain: 17ISAe	654	ASM349136v1	GCF_003491365.1	RefSeq	GCF_003491365.1-RS_2025_03_20	Complete G	4652208	4657278	9/7/2018	4345	PRJNA4388	SAMN08731619
Aeromonas salmonicida subsp. salmonicida 01-B526	strain: 01-B526	1076135	ASM369267v1	GCF_003692675.1	RefSeq	GCF_003692675.1-RS_2026_02_18	Complete G	4736282	4736282	10/29/2018	4501	PRJNA2643	SAMN02469939
Aeromonas veronii	strain: MS-18-37	654	ASM372217v1	GCF_003722175.1	RefSeq	GCF_003722175.1-RS_2025_03_20	Complete G	4683931	4683931	11/13/2018	4292	PRJNA5042	SAMN10389897
Aeromonas caviae	strain: GSH8M-1	648	ASM392585v2	GCF_003925855.2	RefSeq	GCF_003925855.2-RS_2026_02_12	Complete G	4611279	4826820	2/14/2019	4565	PRJDB6962	SAMD00144878
Aeromonas hydrophila	strain: WCX23	644	ASM399946v1	GCF_003999465.1	RefSeq	NCBI Prokaryotic Genome Annotation Pipeline (PGAP)	Complete G	4139625	4304746	1/8/2019	4090	PRJNA4317	SAMN08427316
Aeromonas hydrophila	strain: GSH8-2	644	ASM429643v1	GCF_004296435.1	RefSeq	GCF_004296435.1-RS_2026_02_12	Complete G	4962478	5001549	2/14/2019	4611	PRJDB6962	SAMD00144877
Aeromonas hydrophila	strain: WCX23	644	ASM468430v1	GCF_004684305.1	RefSeq	GCF_004684305.1-RS_2025_07_23	Complete G	5145899	5311033	4/7/2019	4999	PRJNA5300	SAMN11293745
Aeromonas hydrophila	strain: 23-C-23	644	ASM468432v1	GCF_004684325.1	RefSeq	GCF_004684325.1-RS_2025_04_13	Complete G	5117112	5282246	4/7/2019	4965	PRJNA5300	SAMN11293757
Aeromonas schubertii	strain: LF1708	652	ASM491948v1	GCF_004919485.1	RefSeq	GCF_004919485.1-RS_2025_12_04	Complete G	4350713	4350713	4/28/2019	4219	PRJNA5271	SAMN11129962
Aeromonas caviae	strain: WCW1-2	648	ASM717929v1	GCF_007179295.1	RefSeq	GCF_007179295.1-RS_2026_01_30	Complete G	4684096	4684096	7/19/2019	4396	PRJNA5401	SAMN11521871
Aeromonas salmonicida subsp. salmonicida	strain: SHY16-3432	29491	ASM837073v1	GCF_008370735.1	RefSeq	GCF_008370735.1-RS_2025_03_22	Complete G	4739953	5008222	9/12/2019	4802	PRJNA2643	SAMN11172869
Aeromonas veronii	strain: FDAARGOS_632	654	ASM869370v1	GCF_008693705.1	RefSeq	GCF_008693705.1-RS_2025_12_09	Complete G	4559061	4561870	9/25/2019	4199	PRJNA2312	SAMN11056347
Aeromonas veronii	strain: HX3	654	ASM975574v1	GCF_009755745.1	RefSeq	GCF_009755745.1-RS_2025_12_28	Complete G	4604603	4762818	12/16/2019	4434	PRJNA5450	SAMN11868773
Aeromonas hydrophila	strain: HX-3	644	ASM979145v1	GCF_009791455.1	RefSeq	GCF_009791455.1-RS_2025_12_14	Complete G	4941513	4941513	12/23/2019	4541	PRJNA5958	SAMN13567714
Aeromonas veronii	strain: A8-AHP	654	ASM983302v1	GCF_009833025.1	RefSeq	NCBI Prokaryotic Genome Annotation Pipeline (PGAP)	Complete G	4744657	4768216	1/7/2020	4419	PRJNA5878	SAMN13220457
Aeromonas veronii	strain: AVNIH1	654	ASM983406v1	GCF_009834065.1	RefSeq	GCF_009834065.1-RS_2025_03_22	Complete G	4807642	4807642	1/8/2020	4469	PRJNA5968	SAMN13640848
Aeromonas salmonicida subsp. salmonicida	strain: J409	29491	ASM985811v1	GCF_009858115.1	RefSeq	GCF_009858115.1-RS_2025_12_24	Complete G	4632948	4732829	1/13/2020	4550	PRJNA5944	SAMN13518349
Aeromonas salmonicida subsp. salmonicida	strain: J410	29491	ASM985813v1	GCF_009858135.1	RefSeq	GCF_009858135.1-RS_2025_12_24	Complete G	4617865	4690150	1/13/2020	4586	PRJNA5944	SAMN13518350
Aeromonas media	strain: MC64	651	ASM993113v1	GCF_009931135.1	RefSeq	GCF_009931135.1-RS_2025_03_17	Complete G	4710838	5018368	1/27/2020	4694	PRJNA6019	SAMN13876930
Aeromonas veronii	strain: ZfB1	654	ASM1104549v1	GCF_011045495.1	RefSeq	GCF_011045495.1-RS_2025_03_22	Complete G	4711383	4711383	3/2/2020	4378	PRJNA5131	SAMN10696333
Aeromonas hydrophila	strain: LHW39	644	ASM1160242v1	GCF_011602425.1	RefSeq	GCF_011602425.1-RS_2025_04_19	Complete G	5099855	5099855	3/21/2020	4730	PRJNA6118	SAMN14351661
Aeromonas hydrophila	strain: NEB724	644	ASM1227359v1	GCF_012273595.1	RefSeq	NCBI Prokaryotic Genome Annotation Pipeline (PGAP)	Complete G	4573207	4573207	4/12/2020	4238	PRJNA6228	SAMN14533640
Aeromonas salmonicida	strain: SRW-OG1	645	ASM1293158v1	GCF_012931585.1	RefSeq	GCF_012931585.1-RS_2026_03_08	Complete G	4621725	4621725	4/29/2020	4256	PRJNA6224	SAMN14515089
Aeromonas media	strain: T0.1-19	651	ASM1308548v1	GCF_013085485.1	RefSeq	GCF_013085485.1-RS_2025_07_14	Complete G	4917765	4920550	5/14/2020	4600	PRJNA5295	SAMN11269565
Aeromonas sp. 2692-1	strain: 2692-1	2560029	ASM1308550v1	GCF_013085505.1	RefSeq	GCF_013085505.1-RS_2024_07_21	Complete G	5018075	5028423	5/14/2020	4711	PRJNA5289	SAMN11250631
Aeromonas sp. 1805	strain: 1805	2560028	ASM1308552v1	GCF_013085525.1	RefSeq	GCF_013085525.1-RS_2024_07_21	Complete G	4834781	4834781	5/14/2020	4514	PRJNA5289	SAMN11250630
Aeromonas media	strain: R1-18	651	ASM1308572v1	GCF_013085725.1	RefSeq	GCF_013085725.1-RS_2025_07_14	Complete G	4743402	4743402	5/14/2020	4486	PRJNA5295	SAMN11269566
Aeromonas media	strain: T5-8	651	ASM1308574v1	GCF_013085745.1	RefSeq	GCF_013085745.1-RS_2025_07_14	Complete G	4791767	4791767	5/14/2020	4485	PRJNA5295	SAMN11269567
Aeromonas media	strain: R25-3	651	ASM1308576v1	GCF_013085765.1	RefSeq	GCF_013085765.1-RS_2025_07_14	Complete G	4683582	4879157	5/14/2020	4564	PRJNA5295	SAMN11269568

Aeromonas media	strain: R50-22	651	ASM1308578v1	GCF_013085785.1	RefSeq	GCF_013085785.1-RS_2025_07_14	Complete G	4748464	5147209	5/14/2020	4791	PRJNA5295	SAMN11269569
Aeromonas veronii	strain: JC529	654	ASM1341582v1	GCF_013415825.1	RefSeq	GCF_013415825.1-RS_2025_12_29	Complete G	4834659	4834659	7/15/2020	4501	PRJNA6426	SAMN15395818
Aeromonas caviae	strain: NY4617	648	ASM1348796v1	GCF_013487965.1	RefSeq	GCF_013487965.1-RS_2025_05_22	Complete G	4520672	4520672	7/23/2020	4258	PRJNA6016	SAMN13871641
Aeromonas caviae	strain: 1507-17068	648	ASM1348798v1	GCF_013487985.1	RefSeq	GCF_013487985.1-RS_2026_01_29	Complete G	4527876	4527876	7/23/2020	4230	PRJNA6018	SAMN13875147
Aeromonas caviae	strain: 1605-27183	648	ASM1348800v1	GCF_013488005.1	RefSeq	GCF_013488005.1-RS_2026_01_30	Complete G	4861894	4861894	7/23/2020	4581	PRJNA6018	SAMN13875308
Aeromonas media	strain: R1-26	651	ASM1410986v1	GCF_014109865.1	RefSeq	GCF_014109865.1-RS_2026_02_17	Complete G	4665790	4665790	8/7/2020	4373	PRJNA5633	SAMN12671375
Aeromonas caviae	strain: WP3-S18-ESBL-02	648	ASM1415845v1	GCF_014158455.1	RefSeq	GCF_014158455.1-RS_2026_01_30	Complete G	4860108	4860108	7/21/2020	4585	PRJDB6962	SAMD00194421
Aeromonas media	strain: WP7-W18-ESBL-02	651	ASM1416142v1	GCF_014161425.1	RefSeq	GCF_014161425.1-RS_2026_02_13	Complete G	4758243	4758243	7/21/2020	4443	PRJDB6962	SAMD00194563
Aeromonas hydrophila	strain: WP7-S18-ESBL-06	644	ASM1416195v1	GCF_014161955.1	RefSeq	GCF_014161955.1-RS_2026_02_07	Complete G	4940097	4940097	7/21/2020	4547	PRJDB6962	SAMD00194576
Aeromonas caviae	strain: WP8-S17-ESBL-03	648	ASM1416201v1	GCF_014162015.1	RefSeq	GCF_014162015.1-RS_2026_01_29	Complete G	4352153	4352153	7/21/2020	4062	PRJDB6962	SAMD00194595
Aeromonas sp. WP2-W18-CRE-05	strain: WP2-W18-CRE-05	2675707	ASM1416861v1	GCF_014168615.1	RefSeq	GCF_014168615.1-RS_2025_08_25	Complete G	4906952	5069260	7/21/2020	4833	PRJDB6962	SAMD00194348
Aeromonas caviae	strain: WP2-W18-ESBL-01	648	ASM1416863v1	GCF_014168635.1	RefSeq	GCF_014168635.1-RS_2026_02_13	Complete G	4844566	4979818	7/21/2020	4681	PRJDB6962	SAMD00194350
Aeromonas veronii	strain: WP2-S18-CRE-03	654	ASM1416871v1	GCF_014168715.1	RefSeq	NCBI Prokaryotic Genome Annotation Pipeline (PGAP)	Complete G	4834107	4938487	7/21/2020	4705	PRJDB6962	SAMD00194363
Aeromonas veronii	strain: WP3-W19-ESBL-03	654	ASM1416899v1	GCF_014168995.1	RefSeq	GCF_014168995.1-RS_2025_03_17	Complete G	4941307	4980290	7/21/2020	4639	PRJDB6962	SAMD00194437
Aeromonas caviae	strain: WP5-W18-ESBL-02	648	ASM1416923v1	GCF_014169235.1	RefSeq	GCF_014169235.1-RS_2026_01_30	Complete G	4751792	4899778	7/21/2020	4675	PRJDB6962	SAMD00194514
Aeromonas caviae	strain: WP8-S18-CRE-01	648	ASM1416967v1	GCF_014169675.1	RefSeq	GCF_014169675.1-RS_2026_01_30	Complete G	4611279	4826820	7/21/2020	4564	PRJDB6962	SAMD00194620
Aeromonas hydrophila	strain: WP8-S18-ESBL-02	644	ASM1416971v1	GCF_014169715.1	RefSeq	GCF_014169715.1-RS_2026_02_13	Complete G	4962478	5001549	7/21/2020	4611	PRJDB6962	SAMD00194624
Aeromonas caviae	strain: WP8-S18-ESBL-04	648	ASM1416973v1	GCF_014169735.1	RefSeq	GCF_014169735.1-RS_2025_04_03	Complete G	4507652	4565788	7/21/2020	4251	PRJDB6962	SAMD00194626
Aeromonas veronii	strain: WP8-S18-ESBL-11	654	ASM1416979v1	GCF_014169795.1	RefSeq	NCBI Prokaryotic Genome Annotation Pipeline (PGAP)	Complete G	4861422	4907425	7/21/2020	4585	PRJDB6962	SAMD00194633
Aeromonas veronii	strain: WP8-W19-CRE-03	654	ASM1416983v1	GCF_014169835.1	RefSeq	GCF_014169835.1-RS_2025_12_30	Complete G	4555339	4792142	7/21/2020	4466	PRJDB6962	SAMD00194639
Aeromonas veronii	strain: WP9-W18-ESBL-04	654	ASM1416987v1	GCF_014169875.1	RefSeq	GCF_014169875.1-RS_2025_03_17	Complete G	4781224	4927279	7/21/2020	4587	PRJDB6962	SAMD00194675
Aeromonas jandaei	strain: 3348	650	ASM1421748v1	GCF_014217485.1	RefSeq	NCBI Prokaryotic Genome Annotation Pipeline (PGAP)	Complete G	4565418	4565418	8/17/2020	4345	PRJNA5289	SAMN12097417
Aeromonas jandaei	strain: 3299	650	ASM1421750v1	GCF_014217505.1	RefSeq	NCBI Prokaryotic Genome Annotation Pipeline (PGAP)	Complete G	4575997	4575997	8/17/2020	4294	PRJNA5289	SAMN12097416
Aeromonas hydrophila	strain: KAM330	644	ASM1467958v1	GCF_014679585.1	RefSeq	GCF_014679585.1-RS_2026_02_07	Complete G	5092593	5445750	8/29/2020	5146	PRJDB1042	SAMD00242552
Aeromonas salmonicida subsp. masoucida	strain: BR19001YR	197700	ASM1487273v1	GCF_014872735.1	RefSeq	GCF_014872735.1-RS_2025_12_24	Complete G	4780847	4982192	10/8/2020	4788	PRJNA6098	SAMN14260301
Aeromonas caviae	strain: FDAARGOS_72	648	ASM1488389v1	GCF_014883895.1	RefSeq	NCBI Prokaryotic Genome Annotation Pipeline (PGAP)	Complete G	4527600	4527600	10/13/2020	4229	PRJNA6667	SAMN02934523
Aeromonas simiae	strain: A6	218936	ASM1489269v1	GCF_014892695.1	RefSeq	GCF_014892695.1-RS_2025_11_23	Complete G	3974097	3974097	10/9/2019	3785	PRJNA5433	SAMN11664857
Aeromonas caviae	strain: KAM329	648	ASM1550854v2	GCF_015508545.1	RefSeq	GCF_015508545.1-RS_2025_03_23	Complete G	4970632	5171984	12/9/2020	4919	PRJDB1042	SAMD00254948
Aeromonas allosaccharophila	strain: FDAARGOS_933	656	ASM1602661v1	GCF_016026615.1	RefSeq	GCF_016026615.1-RS_2025_12_13	Complete G	4624771	4630407	12/14/2020	4257	PRJNA2312	SAMN13450463
Aeromonas hydrophila	strain: FDAARGOS_916	644	ASM1602687v1	GCF_016026875.1	RefSeq	GCF_016026875.1-RS_2026_02_07	Complete G	4733702	4733702	12/14/2020	4399	PRJNA2312	SAMN13450446
Aeromonas caviae	strain: Aero21	648	ASM1612681v2	GCF_016126815.2	RefSeq	GCF_016126815.2-RS_2024_03_23	Complete G	5123400	5367472	1/25/2021	5481	PRJNA5128	SAMN17014572
Aeromonas jandaei	strain: FDAARGOS_986	650	ASM1612719v1	GCF_016127195.1	RefSeq	GCF_016127195.1-RS_2025_12_13	Complete G	4551079	4551079	12/20/2020	4261	PRJNA2312	SAMN16357155
Aeromonas hydrophila	strain: Aeromonas hydrophila	644	ASM1659229v1	GCF_016592295.1	RefSeq	NCBI Prokaryotic Genome Annotation Pipeline (PGAP)	Complete G	4962727	4962727	12/19/2020	4550	PRJDB1089	SAMD00261012
Aeromonas caviae	strain: Aero52	648	ASM1659881v1	GCF_016598815.1	RefSeq	GCF_016598815.1-RS_2026_01_29	Complete G	4386545	4532165	1/10/2021	4313	PRJNA5128	SAMN17150322
Aeromonas media	strain: E31	651	ASM1665369v1	GCF_016653695.1	RefSeq	GCF_016653695.1-RS_2026_01_29	Complete G	4923283	5311816	1/17/2021	4975	PRJNA6906	SAMN17256260
Aeromonas caviae	isolate: Aero19	648	ASM1672886v1	GCF_016728865.1	RefSeq	GCF_016728865.1-RS_2025_03_23	Complete G	4371624	4482183	1/20/2021	4275	PRJNA5128	SAMN16998432
Aeromonas caviae	strain: KAM376	648	ASM1686142v1	GCF_016861425.1	RefSeq	GCF_016861425.1-RS_2025_03_23	Complete G	4664715	4928144	1/20/2021	4705	PRJDB1042	SAMD00270046
Aeromonas jandaei	strain: JUNP479	650	ASM1686534v1	GCF_016865345.1	RefSeq	GCF_016865345.1-RS_2025_04_01	Complete G	4534922	4541146	2/2/2021	4236	PRJDB1111	SAMD00276024
Aeromonas hydrophila	strain: Brac6	644	ASM1731019v1	GCF_017310195.1	RefSeq	GCF_017310195.1-RS_2025_04_08	Complete G	4765114	4765114	3/9/2021	4377	PRJNA5911	SAMN13353934
Aeromonas hydrophila	strain: OnP3.1	644	ASM1731021v1	GCF_017310215.1	RefSeq	GCF_017310215.1-RS_2025_04_08	Complete G	4766062	4766062	3/9/2021	4376	PRJNA5950	SAMN13545457
Aeromonas dhakensis	strain: 1706-28330	196024	ASM1731023v1	GCF_017310235.1	RefSeq	GCF_017310235.1-RS_2025_04_08	Complete G	4933619	4933619	3/9/2021	4577	PRJNA6382	SAMN15184665
Aeromonas veronii	strain: 1708-29120	654	ASM1731027v1	GCF_017310275.1	RefSeq	GCF_017310275.1-RS_2025_03_23	Complete G	4500467	4500467	3/9/2021	4136	PRJNA6382	SAMN15184730
Aeromonas jandaei	strain: 4956	650	ASM1880226v1	GCF_018802265.1	RefSeq	NCBI Prokaryotic Genome Annotation Pipeline (PGAP)	Complete G	4535455	4653229	6/9/2021	4422	PRJNA5289	SAMN13926354
Aeromonas hydrophila	strain: 4960	644	ASM1880230v1	GCF_018802305.1	RefSeq	GCF_018802305.1-RS_2025_04_13	Complete G	4827247	4827247	6/9/2021	4516	PRJNA5289	SAMN13926351
Aeromonas jandaei	strain: 4608	650	ASM1880232v1	GCF_018802325.1	RefSeq	GCF_018802325.1-RS_2025_04_03	Complete G	4507629	4507629	6/9/2021	4230	PRJNA5289	SAMN13926353
Aeromonas jandaei	strain: 3036	650	ASM1880236v1	GCF_018802365.1	RefSeq	GCF_018802365.1-RS_2025_04_03	Complete G	4592550	4592550	6/9/2021	4339	PRJNA5289	SAMN13926352
Aeromonas hydrophila	strain: 3019	644	ASM1880238v1	GCF_018802385.1	RefSeq	GCF_018802385.1-RS_2025_04_12	Complete G	5130685	5130685	6/9/2021	4855	PRJNA5289	SAMN13926349
Aeromonas sp. FDAARGOS 1402	strain: FDAARGOS 1402	2778051	ASM1904692v1	GCF_019046925.1	RefSeq	GCF_019046925.1-RS_2024_07_26	Complete G	4594505	4922539	6/28/2021	4670	PRJNA2312	SAMN16357544
Aeromonas sp. FDAARGOS 1417	strain: FDAARGOS 1417	2778066	ASM1904698v1	GCF_019046985.1	RefSeq	GCF_019046985.1-RS_2024_07_26	Complete G	4679959	4793224	6/28/2021	4487	PRJNA2312	SAMN16357559
Aeromonas sp. FDAARGOS 1419	strain: FDAARGOS 1419	2778068	ASM1904704v1	GCF_019047045.1	RefSeq	GCF_019047045.1-RS_2024_07_26	Complete G	4588770	4588770	6/28/2021	4202	PRJNA2312	SAMN16357561
Aeromonas sp. FDAARGOS 1414	strain: FDAARGOS 1414	2778063	ASM1904706v1	GCF_019047065.1	RefSeq	GCF_019047065.1-RS_2024_07_26	Complete G	4493834	4493834	6/28/2021	4103	PRJNA2312	SAMN16357556
Aeromonas sp. FDAARGOS 1404	strain: FDAARGOS 1404	2778053	ASM1904708v1	GCF_019047085.1	RefSeq	GCF_019047085.1-RS_2024_07_26	Complete G	4738672	4738672	6/28/2021	4377	PRJNA2312	SAMN16357546
Aeromonas sp. FDAARGOS 1411	strain: FDAARGOS 1411	2778060	ASM1904712v1	GCF_019047125.1	RefSeq	GCF_019047125.1-RS_2024_07_26	Complete G	4539453	4539453	6/28/2021	4164	PRJNA2312	SAMN16357553
Aeromonas sp. FDAARGOS 1403	strain: FDAARGOS 1403	2778052	ASM1904732v1	GCF_019047325.1	RefSeq	GCF_019047325.1-RS_2024_07_26	Complete G	4819779	4819779	6/28/2021	4416	PRJNA2312	SAMN16357545
Aeromonas sp. FDAARGOS 1416	strain: FDAARGOS 1416	2778065	ASM1904808v1	GCF_019048085.1	RefSeq	GCF_019048085.1-RS_2024_07_26	Complete G	4518831	4518831	6/28/2021	4145	PRJNA2312	SAMN16357558
Aeromonas sp. FDAARGOS 1405	strain: FDAARGOS 1405	2778054	ASM1904826v1	GCF_019048265.1	RefSeq	GCF_019048265.1-RS_2024_07_26	Complete G	4520881	4614193	6/28/2021	4226	PRJNA2312	SAMN16357547
Aeromonas sp. FDAARGOS 1415	strain: FDAARGOS 1415	2778064	ASM1904836v1	GCF_019048365.1	RefSeq	GCF_019048365.1-RS_2024_07_26	Complete G	4342599	4346429	6/28/2021	4020	PRJNA2312	SAMN16357557
Aeromonas sp. FDAARGOS 1406	strain: FDAARGOS 1406	2778055	ASM1904856v1	GCF_019048565.1	RefSeq	GCF_019048565.1-RS_2024_07_26	Complete G	4645825	4645825	6/28/2021	4381	PRJNA2312	SAMN16357548
Aeromonas sp. FDAARGOS 1418	strain: FDAARGOS 1418	2778067	ASM1904858v1	GCF_019048585.1	RefSeq	GCF_019048585.1-RS_2024_07_26	Complete G	4454045	4454045	6/28/2021	4171	PRJNA2312	SAMN16357560
Aeromonas sp. FDAARGOS 1408	strain: FDAARGOS 1408	2778057	ASM1904866v1	GCF_019048665.1	RefSeq	GCF_019048665.1-RS_2024_07_26	Complete G	4844434	4844434	6/28/2021	4509	PRJNA2312	SAMN16357550
Aeromonas sp. FDAARGOS 1409	strain: FDAARGOS 1409	2778058	ASM1904870v1	GCF_019048705.1	RefSeq	GCF_019048705.1-RS_2024_07_26	Complete G	4377952	4377952	6/28/2021	4044	PRJNA2312	SAMN16357551
Aeromonas sp. FDAARGOS 1407	strain: FDAARGOS 1407	2778056	ASM1904872v1	GCF_019048725.1	RefSeq	GCF_019048725.1-RS_2024_07_26	Complete G	4694595	4701520	6/28/2021	4419	PRJNA2312	SAMN16357549
Aeromonas sp. FDAARGOS 1410	strain: FDAARGOS 1410	2778059	ASM1904874v1	GCF_019048745.1	RefSeq	GCF_019048745.1-RS_2024_07_26	Complete G	4495463	4510294	6/28/2021	4206	PRJNA2312	SAMN16357552
Aeromonas sanarellii	strain: NS1	633415	ASM1933442v1	GCF_019334425.1	RefSeq	NCBI Prokaryotic Genome Annotation Pipeline (PGAP)	Complete G	5206375	5206375	7/25/2021	5020	PRJNA7460	SAMN20179300
Aeromonas salmonicida subsp. masoucida	strain: RZ6S-1	197700	ASM1944380v1	GCF_019443805.1	RefSeq	GCF_019443805.1-RS_2025_09_07	Complete G	4766402	5191207	8/3/2021	4986	PRJNA6109	SAMN14325646
Aeromonas salmonicida subsp. masoucida	strain: RZ6S-1	197700	ASM1944382v1	GCF_019443825.1	RefSeq	GCF_019443825.1-RS_2025_09_07	Complete G	4764728	5063123	8/3/2021	4896	PRJNA6118	SAMN14351711
Aeromonas media	strain: T5-1	651	ASM1945536v1	GCF_019455365.1	RefSeq	NCBI Prokaryotic Genome Annotation Pipeline (PGAP)	Complete G	4935883	5036592	8/5/2021	4844	PRJNA6609	SAMN15963531
Aeromonas enteropelogenes	strain: FDAARGOS_1459	29489	ASM1993082v1	GCF_019930825.1	RefSeq	GCF_019930825.1-RS_2026_01_29	Complete G	4416959	4416959	9/12/2021	4079	PRJNA2312	SAMN20888876
Aeromonas caviae	strain: KAM345	648	ASM1997277v2	GCF_019972775.1	RefSeq	GCF_019972775.1-RS_2026_01_30	Complete G	4698414	4852288	8/5/2021	4617	PRJDB1180	SAMD00294561
Aeromonas caviae	strain: KAM339	648	ASM1997383v2	GCF_019973835.1	RefSeq	GCF_019973835.1-RS_2026_01_30	Complete G	4680542	5009948	8/5/2021	4771	PRJDB1180	SAMD00294556
Aeromonas enteropelogenes	strain: FDAARGOS_1536	29489	ASM2009717v1	GCF_020097175.1	RefSeq	GCF_020097175.1-RS_2026_01_29	Complete G	4329288	4329288	9/23/2021	3999	PRJNA2312	SAMN21402703
Aeromonas enteropelogenes	strain: FDAARGOS_1510	29489	ASM2009729v1	GCF_020097295.1	RefSeq	GCF_020097295.1-RS_2025_12_12	Complete G	4392400	4392400	9/23/2021	4046	PRJNA2312	SAMN21218866
Aeromonas enteropelogenes	strain: FDAARGOS_1509	29489	ASM2009731v1	GCF_020097315.1	RefSeq	GCF_020097315.1-RS_2026_01_30	Complete G	4540592	4540592	9/23/2021	4240	PRJNA2312	SAMN21218865
Aeromonas rivuli	strain: 20-VB00005	648794	ASM2014957v1	GCF_020149575.1	RefSeq	GCF_020149575.1-RS_2025_11_22	Complete G	4357928	4357928	9/27/2021	4028	PRJNA7471	SAMN20256631
Aeromonas hydrophila	strain: CSUSB2	644	ASM2016225v1	GCF_020162255.1	RefSeq	GCF_020162255.1-RS_2025_12_03	Complete G	4846911	4923206	9/28/2021	4550	PRJNA7650	SAMN21540782
Aeromonas veronii	strain: SW3814	654	ASM2017276v2	GCF_020172765.2	RefSeq	GCF_020172765.2-RS_2025_04_14	Complete G	4684058	4844293	1/25/2022	4496	PRJNA7629	SAMN21418941
Aeromonas caviae	strain: K433	648	ASM2018157v1	GCF_020181575.1	RefSeq	GCF_020181575.1-RS_2025_12_28	Complete G	4682417	4682417	9/30/2021	4389	PRJNA7651	SAMN21545965
Aeromonas dhakensis	strain: 71453	196024	ASM2040530v1	GCF_020405305.1	RefSeq	GCF_020405305.1-RS_2025_12_20	Complete G	4907981	4907981	10/10/2021	4483	PRJNA7594	SAMN21168051
Aeromonas caviae	strain: 71442	648	ASM2040532v1	GCF_020405325.1	RefSeq	GCF_020405325.1-RS_2025_12_14	Complete G	4444683	4444683	10/10/2021	4129	PRJNA7594	SAMN21168050
Aeromonas dhakensis	strain: 71431	196024	ASM2040534v1	GCF_020405345.1	RefSeq	GCF_020405345.1-RS_2025_08_19	Complete G	4771925	4771925	10/10/2021	4379	PRJNA7594	SAMN21168049
Aeromonas hydrophila	strain: 71339	644	ASM2040536v1	GCF_020405365.1	RefSeq	GCF_020405365.1-RS_2024_11_19	Complete G	4793727	4793727	10/10/2021	4406	PRJNA7594	SAMN21168048
Aeromonas hydrophila	strain: 71317	644	ASM2040538v1	GCF_020405385.1	RefSeq	GCF_020405385.1-RS_2025_12_06	Complete G	4695915	4695915	10/10/2021	4294	PRJNA7594	SAMN21168046
Aeromonas media	strain: TR3_1	651	ASM2042312v1	GCF_020423125.1	RefSeq	GCF_020423125.1-RS_2025_12_03	Complete G	4521851	4531033	10/12/2021	4206	PRJNA7306	SAMN19237318
Aeromonas veronii	strain: 32286	654	ASM2054444v1	GCF_020544445.1	RefSeq	GCF_020544445.1-RS_2025_12_29	Complete G	5016477	5016477	10/25/2021	4716	PRJNA6838	SAMN17038035

Aeromonas veronii	strain: 71506	654	ASM2064095v1	GCF_020640955.1	RefSeq	GCF_020640955.1-RS_2025_12_29	Complete G	4661511	4692576	10/27/2021	4284	PRJNA7594	SAMN21168054
Aeromonas caviae	strain: 71485	648	ASM2064097v1	GCF_020640975.1	RefSeq	GCF_020640975.1-RS_2026_01_30	Complete G	4600517	4607215	10/27/2021	4324	PRJNA7594	SAMN21168053
Aeromonas salmonicida subsp. salmonicida	strain: 890054	29491	ASM2068302v1	GCF_020683025.1	RefSeq	GCF_020683025.1-RS_2025_04_08	Complete G	4731770	5044668	10/31/2021	4854	PRJNA7733	SAMN22480857
Aeromonas veronii	strain: A29V	654	ASM2158691v1	GCF_021586915.1	RefSeq	GCF_021586915.1-RS_2025_03_23	Complete G	4526256	4535069	1/25/2022	4181	PRJNA7498	SAMN20667377
Aeromonas caviae	strain: SCLZS52	648	ASM2160998v1	GCF_021609985.1	RefSeq	GCF_021609985.1-RS_2026_01_30	Complete G	4718963	4936888	1/26/2022	4719	PRJNA7976	SAMN25038853
Aeromonas hydrophila	strain: NUITM-VA1	644	ASM2165435v1	GCF_021654355.1	RefSeq	GCF_021654355.1-RS_2026_02_07	Complete G	5078148	5091506	11/27/2021	4730	PRJDB1042	SAMD00424814
Aeromonas caviae	strain: NUITM-VA2	648	ASM2165437v1	GCF_021654375.1	RefSeq	GCF_021654375.1-RS_2025_03_13	Complete G	5035951	5035951	11/27/2021	4749	PRJDB1042	SAMD00424815
Aeromonas caviae	strain: 211703	648	ASM2234394v1	GCF_022343945.1	RefSeq	GCF_022343945.1-RS_2025_04_14	Complete G	4783384	4783384	2/20/2022	4551	PRJNA8063	SAMN25872065
Aeromonas hydrophila	strain: LP0103	644	ASM2255719v1	GCF_022557195.1	RefSeq	GCF_022557195.1-RS_2025_04_11	Complete G	5023649	5023649	3/13/2022	4599	PRJNA8103	SAMN26244239
Aeromonas hydrophila	strain: AC185	644	ASM2263117v1	GCF_022631175.1	RefSeq	GCF_022631175.1-RS_2025_08_31	Complete G	4963702	4963702	3/20/2022	4584	PRJNA8123	SAMN26377314
Aeromonas hydrophila	strain: AC133	644	ASM2263119v1	GCF_022631195.1	RefSeq	GCF_022631195.1-RS_2025_08_31	Complete G	5037528	5037528	3/20/2022	4647	PRJNA8123	SAMN26377313
Aeromonas hydrophila	strain: Aer_Brac66	644	ASM2270081v1	GCF_022700815.1	RefSeq	GCF_022700815.1-RS_2025_04_10	Complete G	4752336	4752336	3/28/2022	4369	PRJNA5779	SAMN13041436
Aeromonas hydrophila	strain: Aer_Pi25.1HTAS	644	ASM2270083v1	GCF_022700835.1	RefSeq	GCF_022700835.1-RS_2025_04_10	Complete G	4814081	4814081	3/28/2022	4469	PRJNA5779	SAMN13041437
Aeromonas hydrophila	strain: Aer_Brac14A	644	ASM2270085v1	GCF_022700855.1	RefSeq	GCF_022700855.1-RS_2025_04_10	Complete G	4758202	4758202	3/28/2022	4381	PRJNA5791	SAMN13104751
Aeromonas dhakensis	strain: Aer_OnIF1	196024	ASM2270309v1	GCF_022703095.1	RefSeq	GCF_022703095.1-RS_2025_04_10	Complete G	4830202	4830202	3/28/2022	4461	PRJNA5775	SAMN13030278
Aeromonas hydrophila	strain: A008N2	644	ASM2275954v1	GCF_022759545.1	RefSeq	GCF_022759545.1-RS_2025_04_11	Complete G	4783511	4783511	3/30/2022	4416	PRJNA8173	SAMN26806291
Aeromonas hydrophila	strain: PartN-Ahydrophila-RM8376	644	ASM2286992v1	GCF_022869925.1	RefSeq	GCF_022869925.1-RS_2025_04_26	Complete G	4733720	4733720	4/11/2022	4396	PRJNA6052	SAMN14078805
Aeromonas veronii	strain: HD6448	654	ASM2292115v1	GCF_022921155.1	RefSeq	GCF_022921155.1-RS_2025_05_11	Complete G	5069244	5198147	4/12/2022	4948	PRJNA7460	SAMN20179696
Aeromonas hydrophila	strain: Ah2111	644	ASM2298283v1	GCF_022982835.1	RefSeq	GCF_022982835.1-RS_2025_05_07	Complete G	5035848	5323258	4/14/2022	4991	PRJNA8236	SAMN27350375
Aeromonas veronii	strain: AV040	654	ASM2310074v1	GCF_023100745.1	RefSeq	GCF_023100745.1-RS_2025_05_12	Complete G	4501581	4501581	4/25/2022	4137	PRJNA8276	SAMN27615187
Aeromonas hydrophila	strain: Ah27	644	ASM2382308v1	GCF_023823085.1	RefSeq	GCF_023823085.1-RS_2025_06_25	Complete G	5102912	5102912	6/21/2022	4736	PRJNA7672	SAMN21893820
Aeromonas dhakensis	strain: b2-100	196024	ASM2392020v1	GCF_023920205.1	RefSeq	GCF_023920205.1-RS_2025_07_04	Complete G	5021892	5021892	6/27/2022	4700	PRJNA7972	SAMN24968113
Aeromonas veronii	strain: 183026	654	ASM2392140v1	GCF_023921405.1	RefSeq	GCF_023921405.1-RS_2025_07_04	Complete G	4565950	4565950	6/27/2022	4233	PRJNA7173	SAMN18498901
Aeromonas caviae	strain: 21006	648	ASM2392152v1	GCF_023921525.1	RefSeq	GCF_023921525.1-RS_2025_07_04	Complete G	4573307	4573307	6/27/2022	4279	PRJNA7174	SAMN18499161
Aeromonas encheleia	strain: SOD01	73010	ASM2411237v1	GCF_024112375.1	RefSeq	GCF_024112375.1-RS_2025_08_14	Complete G	4450115	4450115	7/1/2022	4099	PRJNA8522	SAMN29267038
Aeromonas caviae	strain: FAHZZU2447	648	ASM2421875v1	GCF_024218755.1	RefSeq	GCF_024218755.1-RS_2025_07_20	Complete G	4540521	4792334	7/12/2022	4517	PRJNA8538	SAMN29408258
Aeromonas caviae	strain: KAM471	648	ASM2434769v1	GCF_024347695.1	RefSeq	GCF_024347695.1-RS_2025_12_28	Complete G	4617306	4687290	7/6/2022	4430	PRJDB1314	SAMD00446292
Aeromonas caviae	strain: KAM497	648	ASM2434771v1	GCF_024347715.1	RefSeq	GCF_024347715.1-RS_2025_12_28	Complete G	4622257	4880148	7/6/2022	4568	PRJDB1314	SAMD00446293
Aeromonas caviae	strain: KAM643	648	ASM2434777v1	GCF_024347775.1	RefSeq	GCF_024347775.1-RS_2025_12_28	Complete G	4750427	4856972	7/6/2022	4660	PRJDB1314	SAMD00446296
Aeromonas salmonicida	strain: FN1	645	ASM2450825v1	GCF_024508255.1	RefSeq	GCF_024508255.1-RS_2025_04_09	Complete G	5099029	5099029	8/3/2022	4779	PRJNA8629	SAMN30005339
Aeromonas veronii	strain: GD21SC2322TT	654	ASM2458315v1	GCF_024583155.1	RefSeq	GCF_024583155.1-RS_2025_08_22	Complete G	4699437	4704365	8/7/2022	4359	PRJNA8639	SAMN30068462
Aeromonas hydrophila	strain: GD21SC2284TT	644	ASM2458317v1	GCF_024583175.1	RefSeq	GCF_024583175.1-RS_2025_09_07	Complete G	4487140	4496339	8/7/2022	4093	PRJNA8639	SAMN30068475
Aeromonas veronii	strain: HD6454	654	ASM2497183v1	GCF_024971835.1	RefSeq	GCF_024971835.1-RS_2025_09_20	Complete G	5029035	5104818	9/6/2022	4825	PRJNA7450	SAMN20130520
Aeromonas veronii	strain: jsyz553301	654	ASM2566543v1	GCF_025665435.1	RefSeq	GCF_025665435.1-RS_2025_12_29	Complete G	4716245	4723680	10/12/2022	4419	PRJNA8435	SAMN28748616
Aeromonas caviae	strain: KAM479	648	ASM2600005v1	GCF_026000055.1	RefSeq	GCF_026000055.1-RS_2025_12_29	Complete G	4944632	4992385	10/11/2022	4728	PRJDB1314	SAMD00535935
Aeromonas caviae	strain: SVJ23	648	ASM2601321v2	GCF_026013215.2	RefSeq	GCF_026013215.2-RS_2025_09_22	Complete G	4611279	4611279	4/14/2023	4321	PRJNA8930	SAMN31409143
Aeromonas veronii	strain: C4	654	ASM2601602v1	GCF_026016025.1	RefSeq	GCF_026016025.1-RS_2025_12_30	Complete G	4926096	4926096	11/7/2022	4679	PRJNA8955	SAMN31516430
Aeromonas caviae	strain: BC01	648	ASM2665130v1	GCF_026651305.1	RefSeq	GCF_026651305.1-RS_2025_03_25	Complete G	4583445	4602615	12/5/2022	4321	PRJNA4734	SAMN30100875
Aeromonas caviae	strain: BC02	648	ASM2665132v1	GCF_026651325.1	RefSeq	GCF_026651325.1-RS_2025_03_25	Complete G	4592740	4611910	12/5/2022	4335	PRJNA4734	SAMN30100912
Aeromonas dhakensis	strain: BC03	196024	ASM2665134v1	GCF_026651345.1	RefSeq	GCF_026651345.1-RS_2025_01_19	Complete G	4791622	4791622	12/5/2022	4388	PRJNA4734	SAMN30100940
Aeromonas dhakensis	strain: BC04	196024	ASM2665136v1	GCF_026651365.1	RefSeq	GCF_026651365.1-RS_2025_01_19	Complete G	4936381	4936381	12/5/2022	4554	PRJNA4734	SAMN30100966
Aeromonas dhakensis	strain: BC05	196024	ASM2665138v1	GCF_026651385.1	RefSeq	GCF_026651385.1-RS_2025_01_19	Complete G	4772811	4772811	12/5/2022	4388	PRJNA4734	SAMN30101213
Aeromonas caviae	strain: BC11	648	ASM2665140v1	GCF_026651405.1	RefSeq	GCF_026651405.1-RS_2025_03_25	Complete G	4580677	4599369	12/5/2022	4312	PRJNA4734	SAMN30101264
Aeromonas hydrophila	strain: BC13	644	ASM2665144v1	GCF_026651445.1	RefSeq	GCF_026651445.1-RS_2025_01_19	Complete G	4723898	4723898	12/5/2022	4329	PRJNA4734	SAMN30118181
Aeromonas dhakensis	strain: BC15	196024	ASM2665146v1	GCF_026651465.1	RefSeq	GCF_026651465.1-RS_2025_01_19	Complete G	4907083	4907083	12/5/2022	4559	PRJNA4734	SAMN30118293
Aeromonas jandaei	strain: BC17	650	ASM2665148v1	GCF_026651485.1	RefSeq	GCF_026651485.1-RS_2025_01_19	Complete G	4534625	4534625	12/5/2022	4226	PRJNA4734	SAMN30118332
Aeromonas hydrophila	strain: BC16	644	ASM2665150v1	GCF_026651505.1	RefSeq	GCF_026651505.1-RS_2025_01_19	Complete G	4721140	4721140	12/5/2022	4326	PRJNA4734	SAMN30118295
Aeromonas dhakensis	strain: BC18	196024	ASM2665152v1	GCF_026651525.1	RefSeq	GCF_026651525.1-RS_2025_01_19	Complete G	4627193	4628465	12/5/2022	4255	PRJNA4734	SAMN30120839
Aeromonas hydrophila	strain: BC19	644	ASM2665154v1	GCF_026651545.1	RefSeq	GCF_026651545.1-RS_2025_01_19	Complete G	4703131	4703131	12/5/2022	4334	PRJNA4734	SAMN30120841
Aeromonas sp. BC14	strain: BC14	2970966	ASM2665158v1	GCF_026651585.1	RefSeq	NCBI Prokaryotic Genome Annotation Pipeline (PGAP)	Complete G	4704673	4802739	12/5/2022	4387	PRJNA4734	SAMN30118242
Aeromonas salmonicida subsp. salmonicida	strain: J225	29491	ASM2794765v1	GCF_027947655.1	RefSeq	GCF_027947655.1-RS_2026_02_24	Complete G	4695763	4886316	1/25/2023	4666	PRJNA3102	SAMN32781197
Aeromonas salmonicida subsp. salmonicida	strain: J228	29491	ASM2794767v1	GCF_027947675.1	RefSeq	GCF_027947675.1-RS_2026_02_24	Complete G	4702618	4898098	1/25/2023	4685	PRJNA3102	SAMN32781198
Aeromonas salmonicida	strain: AS3	645	ASM2835565v1	GCF_028355655.1	RefSeq	GCF_028355655.1-RS_2026_03_01	Complete G	4866465	4954811	1/31/2023	4649	PRJNA8963	SAMN31541908
Aeromonas salmonicida	strain: ZAS	645	ASM2835581v1	GCF_028355815.1	RefSeq	GCF_028355815.1-RS_2026_03_01	Complete G	4885367	4885367	1/31/2023	4552	PRJNA8963	SAMN31541907
Aeromonas salmonicida	strain: YK	645	ASM2835599v1	GCF_028355995.1	RefSeq	GCF_028355995.1-RS_2026_03_01	Complete G	4761021	4767385	1/31/2023	4543	PRJNA8963	SAMN31541906
Aeromonas salmonicida	strain: BG	645	ASM2835617v1	GCF_028356175.1	RefSeq	GCF_028356175.1-RS_2026_03_01	Complete G	4779864	4881786	1/31/2023	4682	PRJNA8963	SAMN31541905
Aeromonas salmonicida	strain: AS1	645	ASM2835637v1	GCF_028356375.1	RefSeq	GCF_028356375.1-RS_2026_03_01	Complete G	4712253	4787431	1/31/2023	4581	PRJNA8963	SAMN31541903
Aeromonas salmonicida	strain: AS2	645	ASM2835857v1	GCF_028358575.1	RefSeq	GCF_028358575.1-RS_2026_03_01	Complete G	4717177	4746826	1/31/2023	4517	PRJNA8963	SAMN31541904
Aeromonas hydrophila	strain: 516	644	ASM2862179v1	GCF_028621795.1	RefSeq	GCF_028621795.1-RS_2025_03_25	Complete G	5017959	5017959	2/14/2023	4634	PRJNA9321	SAMN33179879
Aeromonas hydrophila subsp. hydrophila	strain: PSKL.DP	196023	ASM2873627v2	GCF_028736275.2	RefSeq	GCF_028736275.2-RS_2026_01_14	Complete G	4844288	4844288	4/7/2023	4447	PRJNA9339	SAMN33261633
Aeromonas dhakensis	strain: PSKL.DM	196024	ASM2873629v1	GCF_028736295.1	RefSeq	GCF_028736295.1-RS_2026_01_14	Complete G	4820799	4820799	2/19/2023	4402	PRJNA9339	SAMN33261634
Aeromonas bestiarum	strain: YK48	105751	ASM2875115v1	GCF_028751155.1	RefSeq	GCF_028751155.1-RS_2025_03_27	Complete G	4751295	4751295	2/21/2023	4395	PRJNA4965	SAMN10245535
Aeromonas caviae	strain: CYEY0630	648	ASM2888487v1	GCF_028884875.1	RefSeq	GCF_028884875.1-RS_2025_04_14	Complete G	4488138	4691094	2/28/2023	4372	PRJNA9356	SAMN33323122
Aeromonas hydrophila	strain: K522	644	ASM2899409v1	GCF_028994095.1	RefSeq	GCF_028994095.1-RS_2025_04_13	Complete G	5170966	5400912	3/5/2023	5077	PRJNA9374	SAMN33408175
Aeromonas allosaccharophila	strain: K520	656	ASM2902498v1	GCF_029024985.1	RefSeq	GCF_029024985.1-RS_2025_04_02	Complete G	4962292	5493184	3/8/2023	5184	PRJNA9374	SAMN33419673
Aeromonas media	strain: K521	651	ASM2902500v1	GCF_029025005.1	RefSeq	GCF_029025005.1-RS_2025_03_31	Complete G	4603836	4666237	3/8/2023	4330	PRJNA9374	SAMN33419675
Aeromonas caviae	strain: K524	648	ASM2902576v1	GCF_029025765.1	RefSeq	GCF_029025765.1-RS_2025_04_16	Complete G	5022617	5255014	3/8/2023	4976	PRJNA9374	SAMN33453545
Aeromonas hydrophila	strain: K533	644	ASM2902578v1	GCF_029025785.1	RefSeq	GCF_029025785.1-RS_2025_04_11	Complete G	5298984	5633455	3/8/2023	5339	PRJNA9374	SAMN33453547
Aeromonas hydrophila	strain: 516	644	ASM2932091v1	GCF_029320915.1	RefSeq	GCF_029320915.1-RS_2025_04_12	Complete G	5017619	5047072	3/21/2023	4676	PRJNA9399	SAMN33550237
Aeromonas salmonicida	strain: ASscau0313	645	ASM2954192v1	GCF_029541925.1	RefSeq	GCF_029541925.1-RS_2025_04_02	Complete G	4668994	4668994	4/3/2023	4282	PRJNA7998	SAMN25210568
Aeromonas caviae	strain: AC1520	648	ASM2959047v1	GCF_029590475.1	RefSeq	GCF_029590475.1-RS_2025_04_14	Complete G	4484517	4737988	4/5/2023	4473	PRJNA9478	SAMN33867246
Aeromonas veronii	strain: b1-3	654	ASM2963393v1	GCF_029633935.1	RefSeq	GCF_029633935.1-RS_2025_04_10	Complete G	4718637	4727191	4/10/2023	4317	PRJNA7967	SAMN24908263
Aeromonas dhakensis	strain: DAMIAN 12594	196024	ASM2977300v1	GCF_029773005.1	RefSeq	GCF_029773005.1-RS_2025_04_16	Complete G	4697715	4697715	4/17/2023	4338	PRJNA9483	SAMN33901112
Aeromonas caviae	strain: FIONA 16800	648	ASM2977302v1	GCF_029773025.1	RefSeq	GCF_029773025.1-RS_2025_04_16	Complete G	4790585	5317260	4/17/2023	5133	PRJNA9483	SAMN33901114
Aeromonas hydrophila	strain: YURI 17	644	ASM2977304v1	GCF_029773045.1	RefSeq	GCF_029773045.1-RS_2025_04_16	Complete G	5228216	5325528	4/17/2023	5017	PRJNA9483	SAMN33901113
Aeromonas veronii	strain: ANYA 14430	654	ASM2977306v1	GCF_029773065.1	RefSeq	GCF_029773065.1-RS_2025_04_16	Complete G	4575915	4578595	4/17/2023	4359	PRJNA9483	SAMN33901090
Aeromonas veronii	strain: ANYA 16549	654	ASM2977308v1	GCF_029773085.1	RefSeq	GCF_029773085.1-RS_2025_04_16	Complete G	4565387	4577382	4/17/2023	4311	PRJNA9483	SAMN33901093
Aeromonas dhakensis	strain: DAMIAN 19342	196024	ASM2977310v1	GCF_029773105.1	RefSeq	GCF_029773105.1-RS_2025_04_16	Complete G	4996425	5008282	4/17/2023	4621	PRJNA9483	SAMN33901111
Aeromonas veronii	strain: ANYA 19953	654	ASM2977322v1	GCF_029773225.1	RefSeq	GCF_029773225.1-RS_2025_04_16	Complete G	4510909	4510909	4/17/2023	4213	PRJNA9483	SAMN33901104
Aeromonas veronii	strain: LOID 15995	654	ASM2977324v1	GCF_029773245.1	RefSeq	GCF_029773245.1-RS_2025_04_16	Complete G	4612963	4629322	4/17/2023	4359	PRJNA9483	SAMN33901085
Aeromonas veronii	strain: ANYA 18661	654	ASM2977326v1	GCF_029773265.1	RefSeq	GCF_029773265.1-RS_2025_04_16	Complete G	4608668	4685416	4/17/2023	4437	PRJNA9483	SAMN33901100
Aeromonas dhakensis	strain: BOND 14504	196024	ASM2977328v1	GCF_029773285.1	RefSeq	GCF_029773285.1-RS_2025_04_16	Complete G	4848532	4848532	4/17/2023	4480	PRJNA9483	SAMN33901109
Aeromonas dhakensis	strain: BOND 19077	196024	ASM2977330v1	GCF_029773305.1	RefSeq	GCF_029773305.1-RS_2025_04_16	Complete G	4770244	4770244	4/17/2023	4376	PRJNA9483	SAMN33901110
Aeromonas veronii	strain: ANYA 19170	654	ASM2977332v1	GCF_029773325.1	RefSeq	GCF_029773325.1-RS_2025_04_16	Complete G	4601947	4601947	4/17/2023	4345	PRJNA9483	SAMN33901103

Aeromonas veronii	strain: ANYA 18644	654	ASM2977334v1	GCF_029773345.1	RefSeq	GCF_029773345.1-RS_2025_04_16	Complete G	4533601	4535906	4/17/2023	4266	PRJNA9483	SAMN33901099
Aeromonas veronii	strain: ANYA 16702	654	ASM2977336v1	GCF_029773365.1	RefSeq	GCF_029773365.1-RS_2025_04_16	Complete G	4447248	4447248	4/17/2023	4127	PRJNA9483	SAMN33901094
Aeromonas veronii	strain: ANYA 14386	654	ASM2977338v1	GCF_029773385.1	RefSeq	GCF_029773385.1-RS_2025_04_16	Complete G	4583999	4583999	4/17/2023	4323	PRJNA9483	SAMN33901089
Aeromonas veronii	strain: ANYA 15110	654	ASM2977340v1	GCF_029773405.1	RefSeq	GCF_029773405.1-RS_2025_04_16	Complete G	4536836	4536836	4/17/2023	4242	PRJNA9483	SAMN33901091
Aeromonas veronii	strain: ANYA 18263	654	ASM2977342v1	GCF_029773425.1	RefSeq	GCF_029773425.1-RS_2025_04_16	Complete G	4693462	4696697	4/17/2023	4439	PRJNA9483	SAMN33901097
Aeromonas veronii	strain: ANYA 18462	654	ASM2977344v1	GCF_029773445.1	RefSeq	GCF_029773445.1-RS_2025_04_16	Complete G	4529225	4590379	4/17/2023	4278	PRJNA9483	SAMN33901098
Aeromonas veronii	strain: ANYA 18196	654	ASM2977346v1	GCF_029773465.1	RefSeq	GCF_029773465.1-RS_2025_04_16	Complete G	4480764	4480764	4/17/2023	4113	PRJNA9483	SAMN33901095
Aeromonas veronii	strain: ANYA 15694	654	ASM2977348v1	GCF_029773485.1	RefSeq	GCF_029773485.1-RS_2025_04_16	Complete G	4755234	4755234	4/17/2023	4598	PRJNA9483	SAMN33901092
Aeromonas veronii	strain: ANYA 18197	654	ASM2977350v1	GCF_029773505.1	RefSeq	GCF_029773505.1-RS_2025_04_16	Complete G	4529087	4532047	4/17/2023	4198	PRJNA9483	SAMN33901096
Aeromonas veronii	strain: ANYA 14022	654	ASM2977352v1	GCF_029773525.1	RefSeq	GCF_029773525.1-RS_2025_04_16	Complete G	4624384	4634898	4/17/2023	4321	PRJNA9483	SAMN33901088
Aeromonas veronii	strain: LOID 16013	654	ASM2977354v1	GCF_029773545.1	RefSeq	GCF_029773545.1-RS_2025_04_16	Complete G	4613013	4629037	4/17/2023	4354	PRJNA9483	SAMN33901086
Aeromonas veronii	strain: ANYA 13997	654	ASM2977356v1	GCF_029773565.1	RefSeq	GCF_029773565.1-RS_2025_04_16	Complete G	4593380	4593380	4/17/2023	4351	PRJNA9483	SAMN33901087
Aeromonas salmonicida	strain: JNG	645	ASM2985427v1	GCF_029854275.1	RefSeq	GCF_029854275.1-RS_2025_04_22	Complete G	4782790	4916220	4/23/2023	4722	PRJNA9556	SAMN34178809
Aeromonas caviae	strain: W3	648	ASM2996180v1	GCF_029961805.1	RefSeq	GCF_029961805.1-RS_2025_05_12	Complete G	4422374	4479870	5/10/2023	4183	PRJNA8114	SAMN26332081
Aeromonas hydrophila	strain: NN-MR659	644	ASM2996232v1	GCF_029962325.1	RefSeq	GCF_029962325.1-RS_2025_05_13	Complete G	4809855	4809855	5/10/2023	4473	PRJNA8601	SAMN34441011
Aeromonas salmonicida	strain: 57	645	ASM3001159v1	GCF_030011595.1	RefSeq	GCF_030011595.1-RS_2025_05_16	Complete G	4810763	5067177	5/14/2023	4724	PRJNA9667	SAMN34556053
Aeromonas salmonicida	strain: 29	645	ASM3001161v1	GCF_030011615.1	RefSeq	GCF_030011615.1-RS_2025_05_16	Complete G	4634397	4634397	5/14/2023	4268	PRJNA9667	SAMN34556052
Aeromonas veronii	strain: AV066	654	ASM3017845v1	GCF_030178455.1	RefSeq	GCF_030178455.1-RS_2025_06_05	Complete G	4702214	4711390	6/6/2023	4359	PRJNA9771	SAMN35453523
Aeromonas caviae	strain: WZDHY22015	648	ASM3050567v1	GCF_030505675.1	RefSeq	GCF_030505675.1-RS_2025_07_18	Complete G	4419528	4419528	7/19/2023	4088	PRJNA9942	SAMN36419034
Aeromonas caviae	strain: OT55	648	ASM3051698v1	GCF_030516985.1	RefSeq	GCF_030516985.1-RS_2025_07_21	Complete G	4480815	4480815	7/21/2023	4166	PRJNA8508	SAMN29204403
Aeromonas veronii	strain: AS1	654	ASM3060963v1	GCF_030609635.1	RefSeq	GCF_030609635.1-RS_2025_08_08	Complete G	4510740	4510740	8/3/2023	4110	PRJNA9109	SAMN32156161
Aeromonas veronii	strain: HS1906	654	ASM3090852v1	GCF_030908525.1	RefSeq	GCF_030908525.1-RS_2025_08_26	Complete G	4663510	4701639	8/27/2023	4346	PRJNA1006	SAMN37015374
Aeromonas salmonicida subsp. masoucida	strain: wx3-1	197700	ASM3123363v1	GCF_031233635.1	RefSeq	GCF_031233635.1-RS_2025_09_07	Complete G	4764791	4764791	9/7/2023	4377	PRJNA8630	SAMN30009789
Aeromonas caviae	strain: LZSFT54	648	ASM3134493v1	GCF_031344935.1	RefSeq	GCF_031344935.1-RS_2025_09_11	Complete G	4675189	4675189	9/12/2023	4355	PRJNA1011	SAMN37209936
Aeromonas allosaccharophila	strain: BIM B-1809	656	ASM3284986v1	GCF_032849865.1	RefSeq	GCF_032849865.1-RS_2025_12_02	Complete G	4709699	4709699	10/19/2023	4348	PRJNA1026	SAMN37740393
Aeromonas veronii	strain: BIM B-1812	654	ASM3285312v1	GCF_032853125.1	RefSeq	GCF_032853125.1-RS_2025_12_29	Complete G	4954599	5074729	10/19/2023	4748	PRJNA1026	SAMN37740348
Aeromonas dhakensis	strain: RYU-Ah62	196024	ASM3309654v1	GCF_033096545.1	RefSeq	GCF_033096545.1-RS_2024_11_06	Complete G	4885226	4885226	6/21/2023	4521	PRJDB1599	SAMD00613431
Aeromonas media	strain: QST31	651	ASM3317013v1	GCF_033170135.1	RefSeq	GCF_033170135.1-RS_2025_12_04	Complete G	4605026	4605026	10/31/2023	4264	PRJNA1029	SAMN37879127
Aeromonas sp. XH	strain: XH	3081770	ASM3334847v1	GCF_033348475.1	RefSeq	GCF_033348475.1-RS_2025_01_22	Complete G	4336255	4336255	11/6/2023	4028	PRJNA1029	SAMN37866838
Aeromonas sp. CD	strain: CD	3080830	ASM3334937v1	GCF_033349375.1	RefSeq	GCF_033349375.1-RS_2025_01_22	Complete G	4888410	4888410	11/6/2023	4579	PRJNA1029	SAMN37868431
Aeromonas veronii	strain: FS12001	654	ASM3405613v1	GCF_034056135.1	RefSeq	GCF_034056135.1-RS_2025_12_21	Complete G	4711745	4711745	12/4/2023	4272	PRJNA1042	SAMN38324581
Aeromonas dhakensis	strain: 202108B1	196024	ASM3414356v1	GCF_034143565.1	RefSeq	GCF_034143565.1-RS_2025_12_21	Complete G	5017480	5043286	12/6/2023	4673	PRJNA1009	SAMN37176155
Aeromonas caviae	strain: HAMBI_1972	648	ASM3442441v1	GCF_034424415.1	RefSeq	GCF_034424415.1-RS_2026_01_23	Complete G	4578158	4578158	12/17/2023	4240	PRJNA1047	SAMN38562710
Aeromonas allosaccharophila	strain: M01	656	ASM3456799v1	GCF_034567995.1	RefSeq	GCF_034567995.1-RS_2025_12_25	Complete G	5082847	5253402	12/25/2023	5001	PRJNA1051	SAMN38809672
Aeromonas hydrophila	strain: MaAh001	644	ASM3531986v1	GCF_035319865.1	RefSeq	GCF_035319865.1-RS_2026_01_09	Complete G	4893488	4893488	1/9/2024	4519	PRJNA1054	SAMN38937125
Aeromonas dhakensis	strain: 202108C2	196024	ASM3565837v1	GCF_035658375.1	RefSeq	GCF_035658375.1-RS_2026_01_14	Complete G	5002987	5002987	1/15/2024	4619	PRJNA1060	SAMN39246591
Aeromonas hydrophila	strain: MAH-4	644	ASM3634763v1	GCF_036347635.1	RefSeq	GCF_036347635.1-RS_2026_02_02	Complete G	5110450	5437990	2/3/2024	5142	PRJNA1068	SAMN39596696
Aeromonas dhakensis	strain: VA54	196024	ASM3707638v1	GCF_037076385.1	RefSeq	GCF_037076385.1-RS_2025_04_05	Complete G	4850593	4850593	4/5/2024	4477	PRJDB1192	SAMD00580203
Aeromonas enteropelogenes	strain: VA111	29489	ASM3707639v1	GCF_037076395.1	RefSeq	GCF_037076395.1-RS_2025_04_05	Complete G	4618130	4618130	4/5/2024	4256	PRJDB1192	SAMD00580206
Aeromonas enteropelogenes	strain: VA118	29489	ASM3707640v1	GCF_037076405.1	RefSeq	GCF_037076405.1-RS_2025_04_05	Complete G	4618130	4618130	4/5/2024	4256	PRJDB1192	SAMD00580207
Aeromonas dhakensis	strain: VA84	196024	ASM3707641v1	GCF_037076415.1	RefSeq	GCF_037076415.1-RS_2025_04_05	Complete G	4850532	4850532	4/5/2024	4476	PRJDB1192	SAMD00580205
Aeromonas dhakensis	strain: VA6	196024	ASM3710120v1	GCF_037101205.1	RefSeq	GCF_037101205.1-RS_2025_04_05	Complete G	4963294	4968709	4/5/2024	4577	PRJDB1192	SAMD00580201
Aeromonas veronii	strain: VA7	654	ASM3710121v1	GCF_037101215.1	RefSeq	GCF_037101215.1-RS_2025_04_05	Complete G	4546421	4703296	4/5/2024	4439	PRJDB1192	SAMD00580202
Aeromonas veronii	strain: VA60	654	ASM3710122v1	GCF_037101225.1	RefSeq	GCF_037101225.1-RS_2025_04_05	Complete G	4659599	4664386	4/5/2024	4330	PRJDB1192	SAMD00580204
Aeromonas hydrophila	strain: AH230115	644	ASM3776536v1	GCF_037765365.1	RefSeq	GCF_037765365.1-RS_2025_04_10	Complete G	5257035	5267491	4/5/2024	4937	PRJNA1087	SAMN40424197
Aeromonas rivipollensis	strain: BE1	948519	ASM3781113v1	GCF_037811135.1	RefSeq	GCF_037811135.1-RS_2025_03_31	Complete G	4552582	4552582	4/5/2024	4179	PRJNA1089	SAMN40541351
Aeromonas hydrophila	strain: BE2	644	ASM3781589v1	GCF_037815895.1	RefSeq	GCF_037815895.1-RS_2025_04_05	Complete G	4860021	4860021	4/6/2024	4471	PRJNA1089	SAMN40548045
Aeromonas caviae	strain: BE3	648	ASM3781674v1	GCF_037816745.1	RefSeq	GCF_037816745.1-RS_2025_04_05	Complete G	4519888	4573557	4/6/2024	4266	PRJNA1089	SAMN40548085
Aeromonas salmonicida	strain: BE4	645	ASM3781773v1	GCF_037817735.1	RefSeq	GCF_037817735.1-RS_2025_04_05	Complete G	4694577	4732502	4/5/2024	4425	PRJNA1089	SAMN40548087
Aeromonas popoffii	strain: BE5	70856	ASM3781870v1	GCF_037818705.1	RefSeq	GCF_037818705.1-RS_2025_04_05	Complete G	4889561	4889561	4/5/2024	4553	PRJNA1089	SAMN40548428
Aeromonas jandaei	strain: DSM 7311	650	ASM3789069v1	GCF_037890695.1	RefSeq	GCF_037890695.1-RS_2025_04_05	Complete G	4551172	4551172	4/5/2024	4266	PRJNA1087	SAMN40453449
Aeromonas caviae	strain: A20-9	648	ASM3844220v1	GCF_038442205.1	RefSeq	GCF_038442205.1-RS_2025_04_24	Complete G	4460961	4502497	4/25/2024	4203	PRJNA1050	SAMN38754032
Aeromonas hydrophila	strain: SHSK47	644	ASM3971898v1	GCF_039718985.1	RefSeq	GCF_039718985.1-RS_2025_05_22	Complete G	4899146	4899146	5/23/2024	4616	PRJNA9044	SAMN31842810
Aeromonas veronii	strain: Ichip_C_3-7	654	ASM4021510v1	GCF_040215105.1	RefSeq	GCF_040215105.1-RS_2025_06_27	Complete G	4576499	4576499	6/20/2024	4232	PRJNA1119	SAMN41651207
Aeromonas sp. 19NY04SH05-1	strain: 19NY04SH05-1	2920537	ASM4058189v1	GCF_040581895.1	RefSeq	GCF_040581895.1-RS_2024_07_10	Complete G	4634729	4645297	7/11/2024	4307	PRJNA8000	SAMN26198619
Aeromonas caviae	strain: 103	648	UNIL_MWF_103_2024	GCF_041075265.1	RefSeq	GCF_041075265.1-RS_2024_08_07	Complete G	4566420	4566420	8/7/2024	4307	PRJNA9914	SAMN42447754
Aeromonas veronii	strain: Aer12	654	ASM4246567v1	GCF_042465675.1	RefSeq	GCF_042465675.1-RS_2024_10_02	Complete G	4719428	4719428	10/10/2024	4447	PRJNA1140	SAMN42828749
Aeromonas bestiarum	strain: S1-4	105751	ASM4274875v1	GCF_042748755.1	RefSeq	GCF_042748755.1-RS_2024_10_09	Complete G	4817499	4817499	10/10/2024	4398	PRJNA1164	SAMN43895938
Aeromonas dhakensis	strain: CWH5	196024	ASM4309937v1	GCF_043099375.1	RefSeq	GCF_043099375.1-RS_2024_10_15	Complete G	4916354	4916354	10/17/2024	4532	PRJNA1168	SAMN44055949
Aeromonas salmonicida	strain: XN-7	645	ASM4309938v1	GCF_043099385.1	RefSeq	GCF_043099385.1-RS_2024_10_15	Complete G	4660183	4660183	10/17/2024	4251	PRJNA1164	SAMN43881920
Aeromonas hydrophila	strain: CCL1	644	ASM4355312v1	GCF_043553125.1	RefSeq	GCF_043553125.1-RS_2024_10_23	Complete G	5137921	5137921	10/24/2024	4742	PRJNA8073	SAMN25981621
Aeromonas mytilicola subsp. aquatica	strain: A-8	3366579	ASM4433638v1	GCF_044336385.1	RefSeq	GCF_044336385.1-RS_2024_11_05	Complete G	4681979	4681979	11/6/2024	4297	PRJNA1168	SAMN44019497
Aeromonas media	strain: FiBL-4043	651	ASM4501223v1	GCF_045012235.1	RefSeq	GCF_045012235.1-RS_2024_11_19	Complete G	4604924	4604924	11/20/2024	4244	PRJNA6157	SAMN14466695
Aeromonas media	strain: FiBL-2097	651	ASM4501224v1	GCF_045012245.1	RefSeq	GCF_045012245.1-RS_2024_11_19	Complete G	4474566	4474566	11/20/2024	4133	PRJNA6157	SAMN14466694
Aeromonas media	strain: DSM 4881	651	ASM4501480v1	GCF_045014805.1	RefSeq	GCF_045014805.1-RS_2024_11_19	Complete G	4699671	4706660	11/20/2024	4432	PRJNA6157	SAMN14466697
Aeromonas media	strain: FiBL-4359	651	ASM4501481v1	GCF_045014815.1	RefSeq	GCF_045014815.1-RS_2024_11_19	Complete G	4653629	4653629	11/20/2024	4356	PRJNA6157	SAMN14466696
Aeromonas media	strain: FiBL-03	651	ASM4501538v1	GCF_045015385.1	RefSeq	GCF_045015385.1-RS_2024_11_19	Complete G	4667379	4667379	11/20/2024	4274	PRJNA6157	SAMN14466692
Aeromonas media	strain: FiBL-04	651	ASM4501539v1	GCF_045015395.1	RefSeq	GCF_045015395.1-RS_2024_11_19	Complete G	4667378	4667378	11/20/2024	4274	PRJNA6157	SAMN14466693
Aeromonas media	strain: FiBL-02	651	ASM4501540v1	GCF_045015405.1	RefSeq	GCF_045015405.1-RS_2024_11_19	Complete G	4667377	4667377	11/20/2024	4274	PRJNA6157	SAMN14466691
Aeromonas media	strain: FiBL-01	651	ASM4501605v1	GCF_045016055.1	RefSeq	GCF_045016055.1-RS_2024_11_19	Complete G	4667467	4667467	11/20/2024	4285	PRJNA6157	SAMN14466690
Aeromonas veronii	strain: CARB25	654	CARB25	GCF_045277165.1	RefSeq	GCF_045277165.1-RS_2024_11_26	Complete G	4808255	4808255	11/26/2024	4405	PRJNA9043	SAMN32209602
Aeromonas caviae	strain: K523	648	ASM4557140v1	GCF_045571405.1	RefSeq	GCF_045571405.1-RS_2024_12_04	Complete G	4942832	5058295	12/6/2024	4810	PRJNA9410	SAMN33587932
Aeromonas salmonicida	strain: GMT3	645	ASM4610502v1	GCF_046105025.1	RefSeq	GCF_046105025.1-RS_2024_12_17	Complete G	4689837	4689837	12/17/2024	4330	PRJNA1079	SAMN40044484
Aeromonas rivipollensis	strain: DSM 24593	948519	57467	GCF_046226845.1	RefSeq	GCF_046226845.1-RS_2024_12_20	Complete G	4949764	5254087	1/26/2025	4935	PRJNA1068	SAMN39589610
Aeromonas sp. DSM 116730	strain: DSM 116730	3115851	1771	GCF_046581335.1	RefSeq	GCF_046581335.1-RS_2025_01_11	Complete G	4507521	4507521	1/11/2025	4156	PRJNA1068	SAMN39589592
Aeromonas caviae	strain: QZ63	648	ASM4676420v1	GCF_046764205.1	RefSeq	GCF_046764205.1-RS_2025_01_20	Complete G	4867652	4867652	1/20/2025	4604	PRJNA1197	SAMN45813436
Aeromonas salmonicida	strain: JK	645	BMC_JK_Hybrid_WGS	GCF_047276505.1	RefSeq	GCF_047276505.1-RS_2025_01_28	Complete G	4895694	4992654	1/29/2025	4659	PRJNA1210	SAMN46247850
Aeromonas hydrophila	strain: BJ018	644	ASM4777797v1	GCF_047777975.1	RefSeq	GCF_047777975.1-RS_2025_02_16	Complete G	4845396	5059249	2/16/2025	4716	PRJNA1204	SAMN46014603
Aeromonas hydrophila	strain: BJ014	644	ASM4777798v1	GCF_047777985.1	RefSeq	GCF_047777985.1-RS_2025_02_16	Complete G	4851759	5065612	2/16/2025	4728	PRJNA1204	SAMN46014601
Aeromonas caviae	strain: BJ042	648	ASM4777799v1	GCF_047777995.1	RefSeq	GCF_047777995.1-RS_2025_02_16	Complete G	4573255	4573255	2/16/2025	4271	PRJNA1204	SAMN46014604
Aeromonas caviae	strain: BJ093	648	ASM4777801v1	GCF_047778015.1	RefSeq	GCF_047778015.1-RS_2025_02_16	Complete G	4530343	4530343	2/16/2025	4225	PRJNA1204	SAMN46014606
Aeromonas veronii	strain: D3	654	ASM4933185v1	GCF_049331855.1	RefSeq	GCF_049331855.1-RS_2025_04_08	Complete G	4580322	4585250	4/9/2025	4213	PRJNA1236	SAMN47400285
Aeromonas oralensis	strain: AB005	3415010	ASM4966557v1	GCF_049665575.1	RefSeq	GCF_049665575.2-RS_2025_06_11	Complete G	4784854	4784854	8/4/2025	4484	PRJNA1247	SAMN47828375
Aeromonas sp. INTO2	strain: INTO2	3416714	ASM4986060v1	GCF_049860605.1	RefSeq	GCF_049860605.1-RS_2025_04_24	Complete G	4814298	4918020	5/13/2025	4717	PRJNA6361	SAMN47890802

Aeromonas sp. FL131	strain: FL131	3416715	ASM4986061v1	GCF_049860615.1	RefSeq	GCF_049860615.1-RS_2025_04_24	Complete (	5212928	5496357	5/13/2025	5161	PRJNA6361	SAMN47890801
Aeromonas hydrophila	strain: 14B-LG1	644	ASM5003967v1	GCF_050039675.1	RefSeq	GCF_050039675.1-RS_2025_05_01	Complete (	4910032	4910032	5/13/2025	4576	PRJNA1253	SAMN48087642
Aeromonas veronii	strain: Y6	654	ASM5050027v1	GCF_050500275.1	RefSeq	GCF_050500275.1-RS_2025_05_21	Complete (	4704502	4704502	5/22/2025	4367	PRJNA1262	SAMN48452389
Aeromonas caviae	strain: AECA21	648	ASM5051751v1	GCF_050517515.1	RefSeq	GCF_050517515.1-RS_2025_05_23	Complete (	4589869	4593698	5/23/2025	4278	PRJNA8373	SAMN35788868
Aeromonas hydrophila	strain: LP-2	644	LP-2	GCF_050887685.1	RefSeq	GCF_050887685.1-RS_2025_06_12	Complete (	5073006	5073006	6/13/2025	4685	PRJNA1075	SAMN40478023
Aeromonas sp. NJAU223	strain: NJAU223	3115650	ASM5112555v1	GCF_051125555.1	RefSeq	GCF_051125555.1-RS_2025_06_28	Complete (	4533439	4533439	6/29/2025	4178	PRJNA1067	SAMN39552742
Aeromonas salmonicida subsp. masoucida	strain: AD112405	197700	ASM5117501v1	GCF_051175015.1	RefSeq	GCF_051175015.1-RS_2025_07_02	Complete (	4756235	5153913	7/3/2025	4985	PRJNA1275	SAMN49014900
Aeromonas veronii	strain: AV1212	654	ASM5198660v1	GCF_051986605.1	RefSeq	GCF_051986605.1-RS_2025_08_14	Complete (	4666509	4666509	8/14/2025	4290		SAMN50249584
Aeromonas veronii	strain: AV0110	654	ASM5198758v1	GCF_051987585.1	RefSeq	GCF_051987585.1-RS_2025_08_14	Complete (	4666516	4666516	8/14/2025	4276		SAMN50250878
Aeromonas hydrophila	strain: BIDMC_4854	644	ASM5205008v1	GCF_052050085.1	RefSeq	GCF_052050085.1-RS_2025_08_15	Complete (	5291521	5358510	8/16/2025	5062		SAMN41152594
Aeromonas hydrophila	strain: AH-12	644	ASM5205883v1	GCF_052058835.1	RefSeq	GCF_052058835.1-RS_2025_08_16	Complete (	5049431	5057044	8/16/2025	4671		SAMN41005122
Aeromonas hydrophila	strain: AH-7	644	ASM5205884v1	GCF_052058845.1	RefSeq	GCF_052058845.1-RS_2025_08_16	Complete (	5093775	5097438	8/16/2025	4711		SAMN41005123
Aeromonas hydrophila	strain: AH-32	644	ASM5205922v1	GCF_052059225.1	RefSeq	GCF_052059225.1-RS_2025_08_16	Complete (	5017889	5027192	8/16/2025	4645		SAMN41005121
Aeromonas hydrophila	strain: AH-34	644	ASM5205923v1	GCF_052059235.1	RefSeq	GCF_052059235.1-RS_2025_08_16	Complete (	5061999	5071302	8/16/2025	4693		SAMN41005120
Aeromonas hydrophila	strain: AH-40	644	ASM5205932v1	GCF_052059325.1	RefSeq	GCF_052059325.1-RS_2025_08_16	Complete (	4830117	4839291	8/16/2025	4469		SAMN41005119
Aeromonas dhakensis	strain: AD-9	196024	ASM5205933v1	GCF_052059335.1	RefSeq	GCF_052059335.1-RS_2025_08_16	Complete (	4935522	5032669	8/16/2025	4668		SAMN41005118
Aeromonas dhakensis	strain: AD-VN2	196024	ASM5205938v1	GCF_052059385.1	RefSeq	GCF_052059385.1-RS_2025_08_16	Complete (	4938934	4962458	8/16/2025	4570		SAMN41005117
Aeromonas dhakensis	strain: AD-VN1	196024	ASM5205939v1	GCF_052059395.1	RefSeq	GCF_052059395.1-RS_2025_08_16	Complete (	4946310	4969812	8/16/2025	4580		SAMN41005116
Aeromonas dhakensis	strain: AD-1	196024	ASM5205944v1	GCF_052059445.1	RefSeq	GCF_052059445.1-RS_2025_08_16	Complete (	4948174	4971682	8/16/2025	4600		SAMN41005115
Aeromonas dhakensis	strain: AD-27	196024	ASM5205945v1	GCF_052059455.1	RefSeq	GCF_052059455.1-RS_2025_08_16	Complete (	4917222	4940724	8/16/2025	4568		SAMN41005114
Aeromonas dhakensis	strain: AD-69	196024	ASM5205952v1	GCF_052059525.1	RefSeq	GCF_052059525.1-RS_2025_08_16	Complete (	4894198	4894198	8/16/2025	4538		SAMN41005113
Aeromonas dhakensis	strain: AD-92HK1	196024	ASM5205953v1	GCF_052059535.1	RefSeq	GCF_052059535.1-RS_2025_08_16	Complete (	4826777	4826777	8/16/2025	4420		SAMN41005112
Aeromonas hydrophila	strain: 3	644	ASM5206326v1	GCF_052063265.1	RefSeq	GCF_052063265.1-RS_2025_08_16	Complete (	5293136	5360125	8/17/2025	5066		SAMN41152713
Aeromonas hydrophila	strain: 4	644	ASM5206327v1	GCF_052063275.1	RefSeq	GCF_052063275.1-RS_2025_08_16	Complete (	5291348	5357887	8/17/2025	5067		SAMN41152722
Aeromonas hydrophila	strain: 2	644	ASM5206328v1	GCF_052063285.1	RefSeq	GCF_052063285.1-RS_2025_08_16	Complete (	5290960	5357868	8/17/2025	5063		SAMN41152634
Aeromonas hydrophila	strain: x230168	644	ASM5256908v1	GCF_052569085.1	RefSeq	GCF_052569085.1-RS_2025_09_12	Complete (	5062321	5106426	9/13/2025	4785		SAMN49790981
Aeromonas veronii	strain: BL61C	654	ASM5295449v1	GCF_052954495.1	RefSeq	GCF_052954495.1-RS_2025_11_17	Complete (	4667365	4667365	11/17/2025	4378		SAMN37105968
Aeromonas piscicola	strain: BIM B-1806	600645	Api_1806_v1	GCF_053714435.1	RefSeq	GCF_053714435.1-RS_2025_11_26	Complete (	5137694	5137694	11/26/2025	4776	PRJNA1366	SAMN53308736
Aeromonas veronii	strain: 21-AL00009	654	ASM5405444v1	GCF_054054445.1	RefSeq	GCF_054054445.1-RS_2025_12_18	Complete (	4798614	4798614	12/17/2025	4525	PRJNA1367	SAMN53354116
Aeromonas veronii	strain: 23-AL00023	654	ASM5405448v1	GCF_054054485.1	RefSeq	GCF_054054485.1-RS_2025_12_18	Complete (	4593802	4593802	12/17/2025	4245	PRJNA1367	SAMN53354117
Aeromonas salmonicida	strain: SGQ-1	645	ASM5433253v1	GCF_054332535.1	RefSeq	GCF_054332535.1-RS_2026_01_05	Complete (	4874023	4874023	1/4/2026	4470	PRJNA1394	SAMN54328447
Aeromonas dhakensis	strain: A24.2	196024	ASM5434387v1	GCF_054343875.1	RefSeq	GCF_054343875.1-RS_2026_01_05	Complete (	4932020	4955522	1/4/2026	4594	PRJNA1392	SAMN54259227
Aeromonas dhakensis	strain: A4.4T	196024	ASM5434395v1	GCF_054343955.1	RefSeq	GCF_054343955.1-RS_2026_01_05	Complete (	4927244	4950768	1/4/2026	4557	PRJNA1392	SAMN54260784
Aeromonas schubertii	strain: ZQ210705	652	ASM5449551v1	GCF_054495515.1	RefSeq	GCF_054495515.1-RS_2026_01_15	Complete (	4272094	4309674	1/14/2026	4107	PRJNA1399	SAMN54502846
Aeromonas veronii	strain: MRG1	654	ASM5485076v1	GCF_054850765.1	RefSeq	GCF_054850765.1-RS_2026_01_31	Complete (	4673298	4687941	1/30/2026	4282	PRJNA1401	SAMN54577973
Aeromonas salmonicida	strain: AB001	645	ASM5489006v1	GCF_054890065.1	RefSeq	GCF_054890065.1-RS_2026_02_04	Complete (	4742014	4742014	2/2/2026	4418	PRJNA1414	SAMN54917770
Aeromonas hydrophila	strain: I-3	644	ASM5538852v1	GCF_055388525.1	RefSeq	GCF_055388525.1-RS_2026_02_24	Complete (	4857591	4857591	2/24/2026	4472		SAMD00995074
Aeromonas veronii	strain: WL-3	654	ASM5555739v1	GCF_055557395.1	RefSeq	GCF_055557395.1-RS_2026_03_02	Complete (	4923780	4923780	2/28/2026	4604	PRJNA1417	SAMN55022346
Aeromonas dhakensis	strain: NaF-IIUM P1L	196024	ASM5566568v1	GCF_055665685.1	RefSeq	GCF_055665685.1-RS_2026_03_04	Complete (	4879335	4879335	3/4/2026	4513	PRJNA1023	SAMN55936301
Aeromonas salmonicida	strain: Ag001	645	ASM5570211v1	GCF_055702115.1	RefSeq	GCF_055702115.1-RS_2026_03_08	Complete (	4648003	4885720	3/7/2026	4658	PRJNA1429	SAMN56071524
Aeromonas caviae	strain: WW33	648	ASM5575511v1	GCF_055755115.1	RefSeq	GCF_055755115.1-RS_2026_03_10	Complete (	5035954	5035954	3/10/2026	4750	PRJNA1346	SAMN52829531
Aeromonas veronii	strain: R16	654	ASM5611439v1	GCF_056114395.1	RefSeq	GCF_056114395.1-RS_2026_03_20	Complete (	4980353	5188393	3/19/2026	4858	PRJNA1419	SAMN55192490
Aeromonas caviae	strain: R10	648	ASM5611444v1	GCF_056114445.1	RefSeq	GCF_056114445.1-RS_2026_03_20	Complete (	4951975	5116051	3/19/2026	4844	PRJNA1419	SAMN55192487
Aeromonas veronii	strain: R8	654	ASM5611446v1	GCF_056114465.1	RefSeq	GCF_056114465.1-RS_2026_03_20	Complete (	4972554	5204591	3/19/2026	4877	PRJNA1419	SAMN55192486
Aeromonas caviae	strain: R7	648	ASM5611448v1	GCF_056114485.1	RefSeq	GCF_056114485.1-RS_2026_03_20	Complete (	4554405	4811275	3/19/2026	4550	PRJNA1419	SAMN55192485
Aeromonas caviae	strain: R4	648	ASM5611450v1	GCF_056114505.1	RefSeq	GCF_056114505.1-RS_2026_03_20	Complete (	5010794	5173330	3/19/2026	4948	PRJNA1419	SAMN55192484
Aeromonas hydrophila	strain: XS-91-4-1	644	ASM5613562v1	GCF_056135625.1	RefSeq	GCF_056135625.1-RS_2026_03_20	Complete (	5056216	5059856	3/20/2026	4663	PRJNA1435	SAMN56431996
Aeromonas caviae	strain: NCTC12244	648	51438_H01	GCF_900476005.1	RefSeq	GCF_900476005.1-RS_2025_12_14	Complete (	4586140	4586140	6/17/2018	4258	PRJEB6403	SAMEA4475690
Aeromonas encheleia	strain: NCTC12917	73010	51438_G01	GCF_900637545.1	RefSeq	GCF_900637545.1-RS_2025_12_13	Complete (	4542521	4542521	12/20/2018	4236	PRJEB6403	SAMEA4475689
Aeromonas australiensis	isolate: Isolate4	1114880	AM_assembly	GCF_907163065.1	RefSeq	NCBI Prokaryotic Genome Annotation Pipeline (PGAP)	Complete (	4562508	4562508	5/17/2021	4222	PRJEB4417	SAMEA8533644

Table S3: The dRep results

genome	completene	contaminat	strain heter	length	N50	centrality	verdict
A aquatica AE235	76.52	0.69	0	4582304	67531	0	excluded
A australiensis CECT 8023	94.17	0	0	4110035	95095	0	
A bestiarum CCUG 41859	90.15	0	0	4692613	237197	0.984911	
A bivalvium CECT 7113	83.1	0	0	4302589	183019	0	excluded
A caviae NCTC12244	99.97	0	0	4586140	4586140	0.989167	
A dhakensis CIP 107500	95.38	0	0	4711881	165885	0.973408	
A diversa CDC 2478 85	90.85	0	0	4026421	64780	0.999844	
A encheleia NCTC12917	99.91	0	0	4542521	4542521	0.999784	
A enteropelogenes FDAARGOS 1509	100	0	0	4540592	4540592	0.98246	
A eucrenophila CCUG 30340	97.81	0	0	4543675	451600	0.999777	
A finlandensis 4287D	75.94	0	0	4717507	31035	0	excluded
A fluvialis LMG 24681	82.53	0	0	3904228	108949	0	excluded
A hydrophila sub hydrophila ATCC 7966 ATCC 7966	99.97	0	0	4744448	4744448	0.983422	
A jandaei FDAARGOS 986	100	0.29	0	4551079	4551079	0.98927	
A lusitana MDC 2473	91.61	0	0	4537799	161739	0	
A media CCM 3653 GCF 042647745.1	99.97	1.17	0	4881877	4717863	0.999082	
A popoffii CIP 105493	81.42	0.58	0	4762473	113504	0	excluded
A rivipollensis DSM 24593	99.97	0	0	4949764	4949764	0.962615	
A rivuli DSM 22539	90.05	0	0	4534147	155151	0	
A salmonicida NCIMB1102	100	0	0	4495934	4495934	0.990353	
A sanarellii LMG 24682	82.9	0	0	4186903	82664	0	excluded
A schubertii CECT 4240	94.07	0	0	4126255	108810	0.999936	
A sobria CIP 74.33	95.09	0	0	4689240	238489	0.999956	
A taiwanensis LMG 24683	92.01	1.72	0	4230588	75335	0.999427	
A tecta CECT 7082	85.81	0	0	4755221	238229	0	excluded
A veronii CECT 4486	92.32	0	0	4410797	147024	0.964169	
A mytilicola sub aquatica A 8	99.97	0.58	0	4681979	4681979	0.967915	Added
A ichthyocola A 5	93.96	0.63	0	4676890	762425	0.964244	Added
A oralensis AB005	98.89	0	0	4784854	4784854	0.963277	Added
